# Smoking as a risk factor for lung cancer in women and men: a systematic review and meta-analysis

**DOI:** 10.1136/bmjopen-2018-021611

**Published:** 2018-10-03

**Authors:** Linda M O’Keeffe, Gemma Taylor, Rachel R Huxley, Paul Mitchell, Mark Woodward, Sanne A E Peters

**Affiliations:** 1 MRC Integrative Epidemiology Unit at the University of Bristol, Bristol Medical School, University of Bristol, Bristol, UK; 2 Population Health Sciences, Bristol Medical School, University of Bristol, Bristol, UK; 3 UK Centre for Tobacco and Alcohol Studies, School of Experimental Psychology, University of Bristol, Bristol, UK; 4 Department of Psychology, University of Bath, Bath, UK; 5 College of Science, Health and Engineering, La Trobe University, Melbourne, Australia; 6 The George Institute for Global Health, University of New South Wales, Sydney, New South Wales, Australia; 7 Olivia Newton-John Cancer and Wellness Centre, Austin Health and Olivia Newton-John Cancer Research Institute, Heidelberg, Victoria, Australia; 8 The George Institute for Global Health, University of Oxford, Oxford, UK; 9 Department of Epidemiology, John Hopkins University, Baltimore, Maryland, USA

**Keywords:** smoking, systematic review, lung cancer, sex-specific

## Abstract

**Objectives:**

To investigate the sex-specific association between smoking and lung cancer.

**Design:**

Systematic review and meta-analysis.

**Data sources:**

We searched PubMed and EMBASE from 1 January 1999 to 15 April 2016 for cohort studies. Cohort studies before 1 January 1999 were retrieved from a previous meta-analysis. Individual participant data from three sources were also available to supplement analyses of published literature.

**Eligibility criteria for selecting studies:**

Cohort studies reporting the sex-specific relative risk (RR) of lung cancer associated with smoking.

**Results:**

Data from 29 studies representing 99 cohort studies, 7 million individuals and >50 000 incident lung cancer cases were included. The sex-specific RRs and their ratio comparing women with men were pooled using random-effects meta-analysis with inverse-variance weighting. The pooled multiple-adjusted lung cancer RR was 6.99 (95% Confidence Interval (CI) 5.09 to 9.59) in women and 7.33 (95% CI 4.90 to 10.96) in men. The pooled ratio of the RRs was 0.92 (95% CI 0.72 to 1.16; I^2^=89%; p<0.001), with no evidence of publication bias or differences across major pre-defined participant and study subtypes. The women-to-men ratio of RRs was 0.99 (95% CI 0.65 to 1.52), 1.11 (95% CI 0.75 to 1.64) and 0.94 (95% CI 0.69 to 1.30), for light, moderate and heavy smoking, respectively.

**Conclusions:**

Smoking yields similar risks of lung cancer in women compared with men. However, these data may underestimate the true risks of lung cancer among women, as the smoking epidemic has not yet reached full maturity in women. Continued efforts to measure the sex-specific association of smoking and lung cancer are required.

**Strengths and limitations of this study**

- Evidence on the sex-specific association of smoking and lung cancer was meta-analysed in over 7 million participants across 99 cohort studies.
- Several subgroup analyses were performed to examine the robustness of findings across different population subgroups.
- However, the smoking epidemic is not yet fully mature in women and risks of lung cancer in women may still be underestimated.
- Detailed data on smoking behaviour and data on specific subtypes of lung cancer were not available.

## Introduction

Lung cancer is the leading cause of cancer death worldwide with 1.7 million global deaths attributed to cigarette smoking in 2015.^1^ Tobacco use is the leading cause of lung cancer; 55% of lung cancer deaths in women and over 70% of lung cancer deaths in men are due to smoking.^1^ These global estimates, however, mask major differences in smoking prevalence in men and women across populations, with rates below 5% for women in most Asian and African countries to 40% and above for men in many parts of Asia and Eastern Europe.^2^ In addition, smoking behaviour varies significantly by sex. For example, compared with women, men smoke more cigars and pipes,^3^ take puffs of longer duration and leave shorter butts,^4^ which each could potentially predispose them to greater risks of smoking-related lung cancer. Substantial physiological differences between the sexes may also result in sex differences in the effects of smoking, particularly for women. For example, compared with men, women have a smaller lung size and different airway behaviour,^5^ which may increase their susceptibility to lung cancer at lower levels of smoking. A recent meta-analysis showed that cigarette smoking confers a greater coronary hazard in women compared with men, which suggests the possibility that this may also be true for the risk of smoking-related lung cancer.^6^

A study of 50-year trends in smoking-related mortality in the USA found that the relative risks of smoking-related lung cancer mortality were higher in men than women.^7^ However, this sex difference was only apparent in the oldest cohorts with the longest follow-up, possibly reflecting greater cumulative tobacco exposure in men than in women. In contrast, a recent study in Korea, a population where smoking patterns continue to differ between the sexes, suggested that sex differences in the impact of smoking on lung cancer risk exist and differ by histological subtype.^8^ Analyses of a large UK primary care database showed that moderate and heavy smoking more strongly increase the risks of lung cancer in women than in men.^9^

Two recent meta-analyses examined the sex-specific association between smoking and lung cancer. In the most recent of these, men were found to have a greater risk of lung cancer associated with smoking compared with women.^10^ However, virtually all data were from historical case–control studies, which have several limitations, and the three included prospective studies provided contradictory results. While a previous meta-analysis by Lee *et al* 11 included 287 cohort and case–control studies and provided sex-specific estimates, single-sex cohorts were also included, sex differences in the smoking-related risk of lung cancer were not formally compared within studies, and only studies published up to 1999 were included.

To resolve this uncertainty, we performed a systematic review and meta-analysis of prospective cohort studies published to date on the sex-specific association of smoking with the risk of fatal and non-fatal lung cancer. Our systematic review builds on these previous meta-analyses by adding literature from 1999 onwards and restricting the analyses to cohort studies, which are less prone to bias than case–control studies. In addition, we perform several predefined subgroup analyses which have not been performed in meta-analyses of cohort studies included in previous reviews and supplement our findings with results from three sources of individual participant data (IPD), not published previously. An important a priori consideration is the substantial sex difference in the maturity of the smoking epidemic with men being at a more advanced stage than women in most parts of the world.^2^ This would be expected to translate into lower relative risk (RR) estimates for lung cancer in women than in men. Hence, the null hypothesis that smoking confers the same lung cancer hazard in both women and men, would be met if the ratio of the RRs (RRRs) for lung cancer (women:men) was less than unity (reflecting a greater hazard in men than women). However, if the RRRs were found to be unity (or higher) then this would suggest a greater hazard associated with tobacco exposure in women than in men.

## Methods

### Search strategy

This review was conducted using a predefined protocol and in accordance to the Meta-analysis Of Observational Studies in Epidemiology guidelines (online supplementary eappendix 1). We systematically searched PubMed and EMBASE for studies published between 1 January 1999 and 15 April 2016 that reported on the relationship between smoking and lung cancer in men and women from a general population. The computer-based searches combined medical subject headings and free-text terms related to ‘tobacco/smoking’, ‘cancer’, ‘sex’ and ‘cohort studies’. The full search criteria are available in online supplementary eappendix 2. Articles published before 1 January 1999 were retrieved from a previous systematic review.^11^ The reference lists of all relevant original research and review articles were scanned to capture missed studies. Two authors (LMOK and GT) independently conducted the screening of studies and any disagreement was mediated by a third author (SAEP).

10.1136/bmjopen-2018-021611.supp1 Supplementary file 1

### Data extraction

Data were extracted, in duplicate, from studies deemed to meet the eligibility criteria. These included details on general study characteristics (study name, duration of follow-up, year of publication), information about the studied population (prevalence of smoking, mean age, number of men and women, incidence of lung cancer, whether lung cancer was fatal or non-fatal and level of adjustment for covariates). We extracted sex-specific adjusted measures of RR and 95% confidence intervals (CIs).

### Study selection

Observational cohort studies were included if they reported sex-specific RRs or equivalent, on the relationship between smoking and lung cancer. Studies were excluded if the variability around the point estimate was not reported, if they had not been adjusted for at least age, or if the study was performed in a population selected on the basis of prior lung cancer or another major underlying chronic disease. In the case of duplicate reports from the same study, the report with the longest follow-up or the highest number of cases was included. IPD from studies available to the authors were also used; the Asia Pacific Cohort Studies Collaboration (APCSC), the National Health and Nutrition Examination Survey III (NHANES III) and the Scottish Heart Health Extended Cohort Study (SHHEC). The Newcastle-Ottawa Scale assessment (NOS) was used to assess the methodological quality of all included studies, on a 9-point scale (online supplementary eappendix 3 and etable 1).^12^

### Meta-analysis

The primary analysis was a comparison of the sex-specific RR of lung cancer (fatal or non-fatal) in current smokers versus non-smokers (defined either as former or never smokers). For each study, we obtained the natural log of the sex-specific RRs and calculated the differences. The differences were pooled across studies using random-effects meta-analysis which allows the RR of lung cancer to vary from study to study, weighted by the inverse of the variances of the log RRs and then back-transformed to obtain the pooled women-to-men RRRs. The SE of the log RRR was calculated as the square root of the sum of the variance of the two sex-specific log RRs for each study. Pooled RRRs were computed separately for studies with only age-adjusted estimates and then for those studies with multiple-adjusted estimates. The set of multiple adjustments made was allowed to vary by study, but had to include at least one other risk factor in addition to age. The I² statistic was used to estimate the percentage of variability across studies due to between-study heterogeneity. The presence of publication bias was graphically examined using contour funnel plots, plotting the natural log of the RRR against its SE and tested using Begg’s test. Predefined subgroup analyses were conducted to obtain the adjusted RRRs by study region (Asia or non-Asia and Asia, Europe, USA, and Australia and New Zealand (ANZ)), year of study baseline (pre-1985 or post-1985), study endpoint (fatal only or fatal and non-fatal combined), number of cigarettes smoked per day (>0 to 10, 10–20, >20), study quality ≤6 vs >6 points) and follow-up time (≤10 vs >10 years). Random-effects meta-analyses were used for all subgroup analyses and differences between subgroups were examined using meta-regression. To include the largest number of studies available, we combined the age-adjusted and multiple-adjusted estimates, taking the maximum adjustment set available. In secondary analyses, we obtained the sex-specific RRs and RRRs comparing former smokers to never smokers and performed the same set of subgroup analyses. All analyses were performed using Stata V.12.0.

### Patient and public involvement

There were no patients or applicable public involved in this review.

## Results

**Figure 1:**
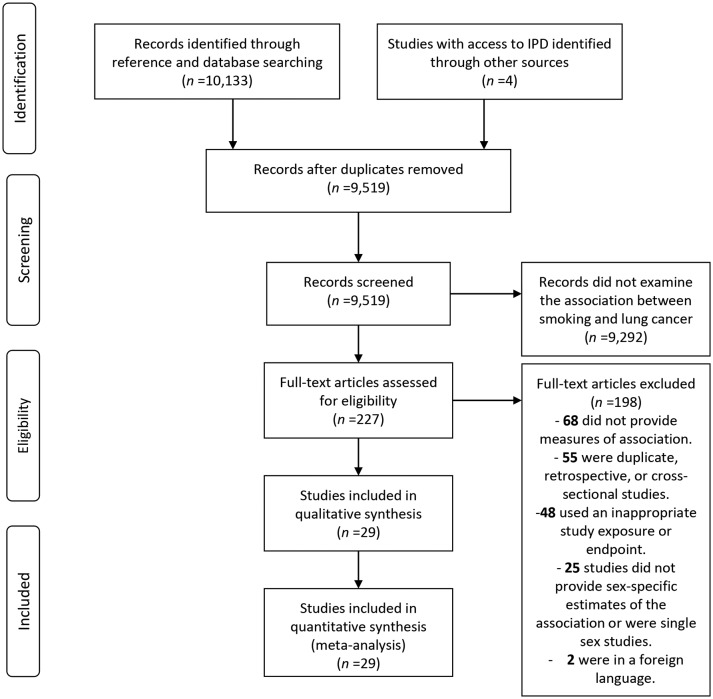
Flow chart of study selection. IPD, individual participant data.

Of the 9519 unique records that were identified through the systematic search, 227 qualified for full-text evaluation (figure 1). Of these, 25 separate studies provided information about sex differences in the association between smoking and lung cancer. This database was extended with IPD from APCSC (separately for Asia and ANZ), NHANES III and SHHEC leading to a total of 29 individual estimates, representing a total of 99 cohort studies available for meta-analysis.

The characteristics of the included studies are described in table 1. Overall, data were available from 99 cohorts, including 7 113 303 individuals (46% women)—not accounting for two cohorts that used Census data—and at least 51 161 incident cases of lung cancer (31% women). Forty-six cohorts were from Asia (61% of the individuals), 6 were from the USA (28%), 37 were from Europe (10%) and 10 were from ANZ (1%). Of 29 studies, 4 studies had a quality score of 5 out of 9, 9 studies had a score of 6, 12 studies had a score of 7 and 4 studies with a score of 8 (online supplementary etable 1).

**Table 1:** Characteristics of included studies

Study name*	Location	Age range, (year)	Baseline year	Follow-up, (years)	NOS score (points)	N (%) women	N (%) lung cancer in women	Fatal/non-fatal	Current smoker, % W/M		Former smoker, % W/M		Maximum available adjustment
APCSC—ANZ^31^ (8 cohorts)	ANZ	20–107	1961–1993	10	6	87 130 (52)	501 (28)	F	7	11	11	18	Age, education, BMI
APCSC—Asia^31^ (33 cohorts)	Asia	20–104	1989–1996	7	6	476 755 (35)	1275 (19)	F	2	59	<1	7	Age, education, BMI
ARIC^32^ (1 cohort)	USA	45–64	1987–1989	20	8	14 610 (54)	470 (37)	F/NF	25	44	22	28	Age, race
China Kadoorie Biobank^33 34^ (1 cohort)	China	35–74	2004–2008	7	7	512 891 (59)	1953 (37)	F	3	61	1	15	Age, area, education, alcohol
China National Hypertension Survey^35^ (1 cohort)	China	15+	1991	8	7	155 131 (51)	NR	F	14	63	NR	NR	Age, education, region, HT, overweight/obesity, alcohol, PA, DM
Copenhagen Cohort Studies^36^ (3 cohorts)	Denmark	20+	1964–1992	14	6	30 874 (44)	867 (23)	F/NF	NR	NR	NR	NR	Age
CPS I (1 cohort)	USA	55+	1959	6	5	518 982 (65)	1293 (21)	F	15	40	4	17	Age, education, race
CPS II^37^ (1 cohort)	USA	55+	1982	6	5	746 485 (61)	4957 (36)	F	18	24	21	43	Age, education, race
EHS^38 39^ (1 cohort)	China	65+	1998–2000	11	7	65 510 (66)	1096 (27)	F	4	10	8	20	Age, education, alcohol, depression, health status, social security assistance, housing type, expenditure
EPIC^40^ (23 cohorts)	Europe	30–70	1992–2000	11	8	441 211 (70)	2995 (49)	F/NF	21	27	25	38	Age, education, BMI, alcohol, PA, energy intake, diet
JPHC,^41^ JACC, TPCS^37 42 43^ (3 cohorts)	Japan	40–79	1983–1994	10	6	296 836 (53)	NR	F	8	54	2	25	Age
Korean Cancer Prevention Study^44^ (1 cohort)	Korea	30–95	1993–1995	9	6	1 212 906 (32)	4238 (14)	F	5	57	3	23	Age
Korean National Health Insurance Service^8^ (1 cohort)	Korea	20+	1998–1999	10	7	1 355 891 (31)	6491 (15)	F/NF	1	56	1	13	Age, BMI, alcohol, PA
Malmo Preventive Project^45^ (1 cohort)	Sweden	27–61	1974–1992	24	5	33 346 (33)	436 (21)	F	36	49	19	27	Age, FEV, SES, marital status
Migrant Study^46^ (1 cohort)	Norway	33–72	1964–1965	21	7	26 126 (55)	435 (23)	F	26	46	8	28	Age
New Zealand Census 1981^47^ (1 cohort)	New Zealand	25+	1981	5	7	NR	4188 (28)	F/NF	NR	NR	NR	NR	Age, race
New Zealand Census 1996^47^ (1 cohort)	New Zealand	25+	1996	5	7	NR	4467 (44)	F/NF	NR	NR	NR	NR	Age, race
NHANES III (1 cohort)	USA	18–90	1988–1994	13	7	20 006 (53)	320 (36)	F	21	30	17	32	Age, education, BMI
NHIS^48^ (1 cohort)	USA	25+	1997–2004	7	7	202 248 (56)	1223 (44)	F	21	26	20	29	Age, education, BMI, alcohol
NIH-AARP^7 49^ (1 cohort)	USA	50–71	1995–1996	11	7	452 131 (41)	9381 (37)	F/NF	17	13	39	61	Age, education, alcohol, ethnicity
Norwegian Counties Study^50–52^ (1 cohort)	Norway	20–49	1974–1978	23	6	48 682 (48)	686 (35)	F	43	50	11	18	Age, SBP, TC, TG, PA, BMI, height, sickness leave, disability pension, family history of CHD
Renfrew/Paisley Study^53^ (1 cohort)	Scotland	45–64	1972–1976	20	6	15 393 (54)	NR	F	47	59	7	25	Age
Reykjavik Study^54^ (1 cohort)	Iceland	32–60	1967	27	7	22 946 (50)	472 (42)	F/NF	39	30	15	24	Age
Shanghai Health Study^55^ (2 cohorts)	China	40–74	1997–2006	10	7	1 34 335 (54)	839 (49)	F	3	70	NR	NR	Age, education, BMI, alcohol, PA, income
SHHEC (1 cohort)	Scotland	25–75	1984–1987	23	6	17 731 (51)	558 (38)	F	40	51	21	27	Age, education, BMI
Singapore Chinese Health Study^56^ (1 cohort)	Singapore	45–74	1993–1998	12	8	61 320 (55)	905 (31)	F/NF	6	36	3	21	Age, dialect, year of recruitment, education, alcohol, PA
Swedish Smoking Habit Survey^57 58^ (1 cohort)	Sweden	18–69	1963	33	8	41 544 (60)	442 (45)	F	18	27	5	23	Age, area
Swiss National Cohort^59^ (4 cohorts)	Switzerland	14–99	1977–1993	19	5	35 703 (53)	426 (29)	F	27	43	13	24	Age, survey, education, alcohol, PA, civil status, nationality, diet
Wen *et al* 60 (2 cohorts)	Taiwan	35+	1982–1992	20	5	86 580 (39)	247 (2)	F	1	41	<1	11	Age

*Note that where several studies are cited for a single cohort, data may be extracted from multiple studies if all data required is not available in the most up-to-date and relevant study.

ANZ, Australia and New Zealand; ARIC, Atherosclerosis Risk in Communities study; APCSC, Asia Pacific Cohort Studies Collaboration; BMI, body mass index; CHD, coronary heart disease; CPS, Cancer Prevention Study; DM, diabetes mellitus; EHS, Elderly Health Services; EPIC, European Prospective Investigation into Cancer; F, fatal; FEV, forced expiratory volume; JACC, Japan Collaborative Cohort Study; JPHC, Japan Public Health CentreStudy; NF, non-fatal; NHANES III, National Health And Nutrition Examination Survey III; NHIS, National Health Interview Survey; NIH-AARP, National Institutes of Health American Association of Retired Persons Diet and Health Study; NOS, Newcastle-Ottawa Scale; NR, not reported; PA, physical activity; SBP, systolic blood pressure; SES, socioeconomic status; SHHEC, Scottish Heart Health Extended Cohort Study; TC, total cholesterol; TG, triglycerides; TPCS, Three-Prefecture Cohort Study.

Eighteen studies reported on the prevalence of smoking, which varied widely by study, region and sex. The prevalence of smoking ranged from 1% to 47% in women and from 1% to 70% in men. In all but two studies, the prevalence of smoking was higher in men than women, especially in Asia where typically less than 10% of women were smokers compared with over 50% of men. Smoking cessation rates were also higher among men (7%–61%) than women (<1%–39%).

### Risk of lung cancer in current smokers versus non-smokers

**Figure 2:**
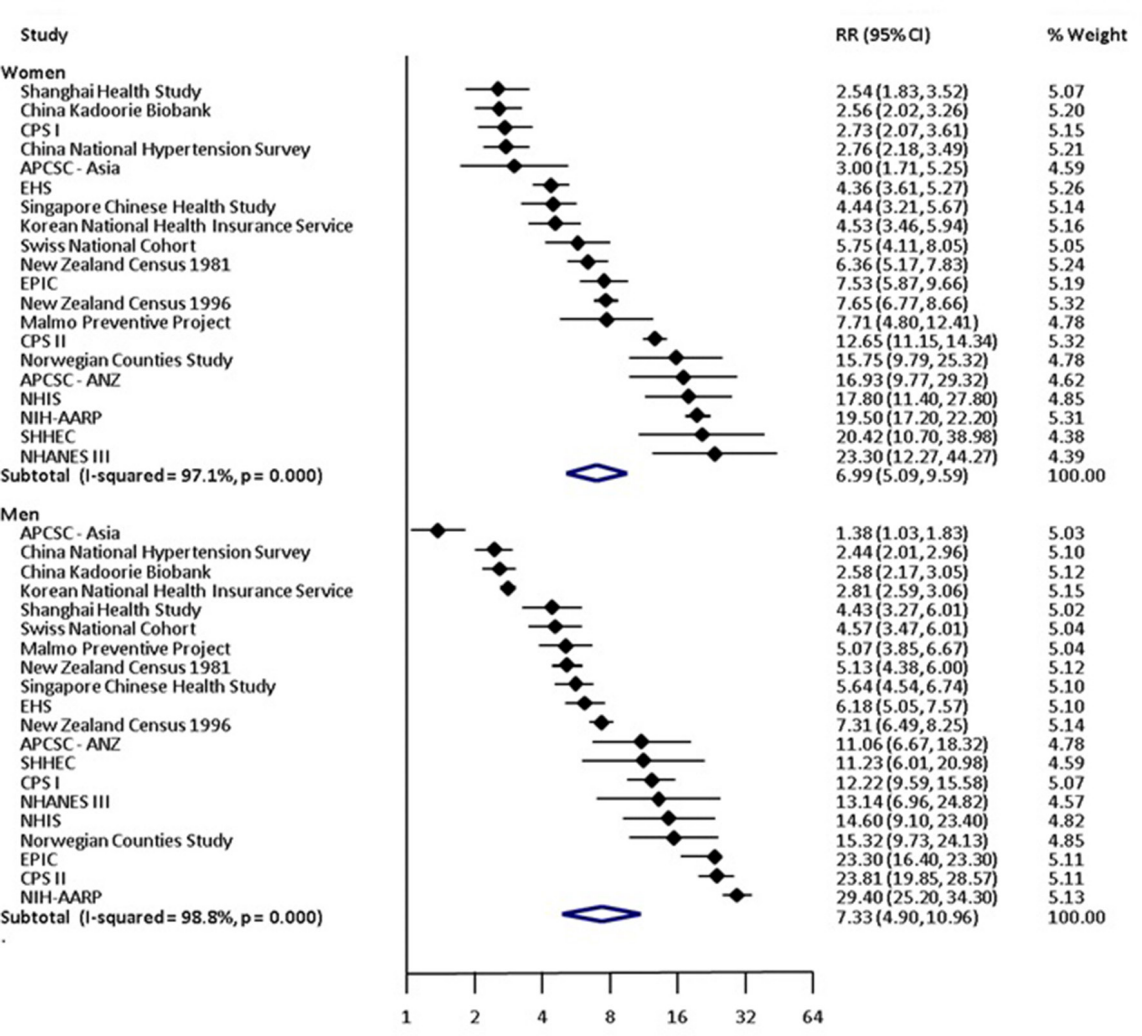
Multiple-adjusted relative risk (RR) for incident lung cancer in women and men, comparing current smokers to non-smokers. Multiple-adjusted includes anything that adjusted for more than just age. These covariates are listed in table 1. Figures may contain less than 29 studies because we report age-adjusted and multiple-adjusted results separately. Some studies only contributed age-adjusted results whereas others only provided multiple-adjusted results. However, the count of unique studies that contributed to at least one of these analyses is 29. APCSC, Asia Pacific Cohort Studies Collaboration; ARIC, Atherosclerosis Risk in Communities study; CPS, Cancer Prevention Study; EHS, Elderly Health Services; EPIC, European Prospective Investigation into Cancer; JACC, Japan Collaborative Cohort Study; JPHC, Japan Public Health Centre Study; NHANES III, National Health And Nutrition Examination Survey III; NHIS, National Health Interview Survey; NIH-AARP, National Institutes of Health American Association of Retrired Persons Diet and Health Study; SHHEC, Scottish Heart Health Extended Cohort Study; TPCS, Three-Prefecture Cohort Study.

**Figure 3:**
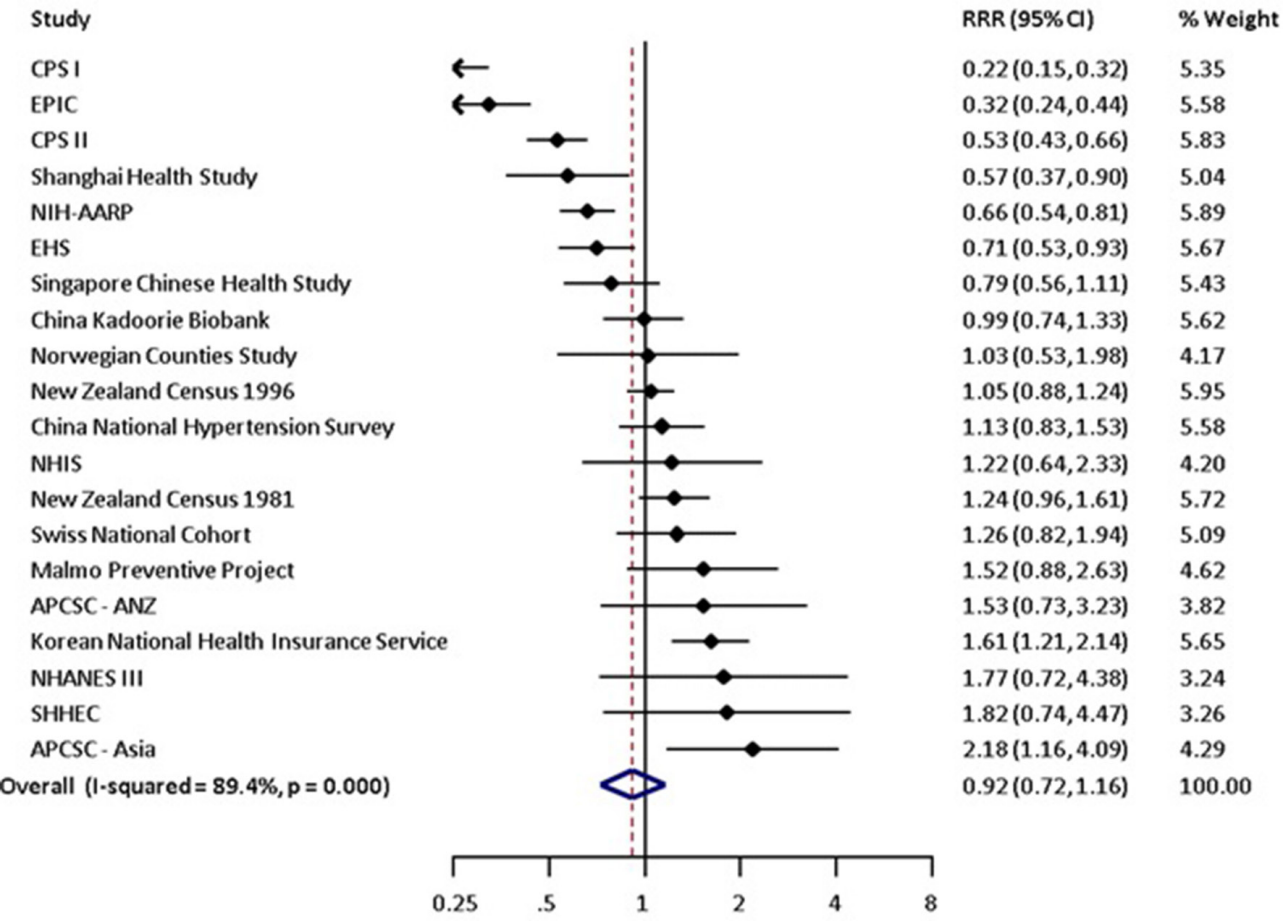
Multiple-adjusted women-to-men ratio of relative risks (RRR) for incident lung cancer, comparing current smokers to non-smokers. Multiple-adjusted includes anything that adjusted for more than just age. These covariates are listed in table 1. Figures may contain less than 29 studies because we report age-adjusted and multiple-adjusted results separately. Some studies only contributed age-adjusted results whereas others only provided multiple-adjusted results. However, the count of unique studies that contributed to at least one of these analyses is 29. APCSC, Asia Pacific Cohort Studies Collaboration; ARIC, Atherosclerosis Risk in Communities study; CPS, Cancer Prevention Study; EHS, Elderly Health Services; EPIC, European Prospective Investigation into Cancer; JACC, Japan Collaborative Cohort Study; JPHC, Japan Public Health Centre Study; NHANES III, National Health And Nutrition Examination Survey III; NHIS, National Health Interview Survey; NIH-AARP, National Institutes of Health American Association of Retired Persons Diet and Health Study; SHHEC, Scottish Heart Health Extended Cohort Study; TPCS, Three-Prefecture Cohort Study.

Compared with non-smoking, current smoking was associated with an age-adjusted RR of lung cancer of 7.48 (95% CI 5.29 to 10.60) in women and 8.78 (95% CI 6.13 to 12.57) in men (table 2 and online supplementary efigure 1). The pooled age-adjusted women-to-men RRR was 0.81 (95% CI 0.62 to 1.04), with substantial between-study heterogeneity (I^2^=86%; p<0.001) (table 2 and online supplementary efigure 2). The multiple-adjusted RR of lung cancer associated with current smoking was 6.99 (95% CI 5.09 to 9.59) in women and 7.33 (95% CI 4.90 to 10.96) in men (table 2 and figure 2). The corresponding RRR was 0.92 (95% CI 0.72 to 1.16) and between-study heterogeneity was substantial (I^2^=89%; p<0.001) (table 2 and figure 3). There was no evidence of publication bias based on the Begg’s test (p=0.75) (online supplementary efigure 3).

**Table 2:** Sex-specific pooled relative risks (RR) and ratio of relative risks (RRR) for lung cancer associated with smoking

	RR in women	RR in men	RRR
Age adjusted			
Former versus never	2.82 (2.25 to 3.54)	3.01 (2.23 to 4.08)	0.88 (0.69 to 1.14)
Current versus not	7.48 (5.29 to 10.60)	8.78 (6.13 to 12.57)	0.81 (0.62 to 1.04)
Multiple adjusted			
Former versus never	3.14 (2.45 to 4.03)	3.13 (2.06 to 4.76)	0.89 (0.69 to 1.13)
Current versus not	6.99 (5.09 to 9.59)	7.33 (4.90 to 10.96)	0.92 (0.72 to 1.16)
Maximum adjusted			
Former versus never	2.92 (2.35 to 3.63)	3.08 (2.31 to 4.11)	0.86 (0.71 to 1.05)
Current versus not	7.32 (5.58 to 9.61)	8.05 (5.90 to 10.98)	0.89 (0.73 to 1.08)
Cigarettes per day among current smokers versus never (maximum available adjusted)			
10 or less	5.30 (3.52 to 7.97)	4.97 (2.74 to 9.03)	0.99 (0.65 to 1.52)
10 to 19	10.67 (7.43 to 15.33)	8.93 (4.90 to 16.28)	1.11 (0.75 to 1.64)
20 or more	17.09 (12.11 to 24.11)	14.61 (8.33 to 25.59)	0.94 (0.69 to 1.30)

Multiple adjusted includes anything that adjusted for more than just age. Maximum available adjustment refers to the most adjustments provided in the study. For some studies, this would have been age adjusted whereas other studies adjusted for more factors than age only (ie, multiple adjusted). These covariates are listed in table 1.

The sex difference in the risk of smoking-related lung cancer in our main analysis did not differ in subgroup analyses stratified by the women-to-men ratio of current smokers (p=0.90), women-to-men ratio of lung cancer incidence in the studies (p=0.64), year of study baseline (p=0.66), study endpoint (p=0.21) or study region (p=0.73) (table 3). The sex difference in the risk of smoking-related lung cancer in our main analysis also did not differ by follow-up time (p=0.83) or study quality (p=0.69). The RRR was 0.93 (95% CI 0.72 to 1.20) for studies from Asia and 0.87 (95% CI 0.66 to 1.14) for studies from USA, Europe or ANZ.

**Table 3:** Maximally adjusted pooled women to men ratio of relative risks (RRR) for lung cancer associated with smoking, in subgroup analyses

	N studies	Former versus never	P for interaction*	N studies	Current versus not	P for interaction*
Study region						
Asia	40	1.07 (0.83 to 1.37)		46	0.93 (0.72 to 1.20)	
Non-Asia	49	0.73 (0.58 to 0.93)	0.06	53	0.87 (0.66 to 1.14)	0.73
Study region						
Asia	40	1.07 (0.83 to 1.37)		46	0.93 (0.72 to 1.20)	
USA	5	0.60 (0.42 to 0.84)		6	0.58 (0.37 to 0.91)	
Europe	36	0.81 (0.60 to 1.10)		37	0.99 (0.63 to 1.57)	
ANZ	8	1.41 (0.65 to 3.04)	0.55	10	1.11 (0.97 to 1.28)	0.69
Year of study baseline						
1985 or before	23	0.79 (0.56 to 1.12)		25	0.96 (0.66 to 1.40)	
After 1985	66	0.92 (0.73 to 1.16)	0.43	74	0.85 (0.68 to 1.06)	0.66
Women-to-men smoking prevalence						
>67% lower in women	39	1.16 (0.83 to 1.62)		43	0.96 (0.72 to 1.28)	
33%–67% lower in women	19	0.72 (0.51 to 1.03)		20	0.75 (0.50 to 1.14)	
0%–33% lower in women	28	0.78 (0.59 to 1.03)	0.26	29	0.97 (0.59 to 1.58)	0.90
Women-to-men lung cancer rate						
≥50% lower in women	80	0.85 (0.65 to 1.10)		83	0.85 (0.62 to 1.17)	
0%–50% lower in women	6	0.83 (0.61 to 1.13)	0.79	9	0.94 (0.68 to 1.31)	0.64
Study endpoint						
Fatal lung cancer only	57	0.94 (0.68 to 1.29)		67	0.97 (0.77 to 1.21)	
Fatal and non-fatal lung cancer	32	0.80 (0.64 to 1.01)	0.57	32	0.72 (0.48 to 1.06)	0.21
Duration of follow-up						
≤10 years	48	0.90 (0.60 to 1.35)		53	0.91 (0.68 to 1.24)	
>10 years	41	0.85 (0.71 to 1.05)	0.92	46	0.87 (0.67 to 1.12)	0.83
Study quality						
≤6 points	58	0.85 (0.64 to 1.13)		61	0.84 (0.53 to 1.12)	
>6 points	31	0.89 (0.66 to 1.20)	0.81	38	0.93 (0.72 to 1.20)	0.69

Random-effects meta-analyses were used for all subgroup analyses and differences between subgroups were examined using meta-regression.

*P for interaction assessed using meta-regression.

ANZ, Australia and New Zealand.

The risk of smoking-related lung cancer increased according to the number of cigarettes smoked per day in both sexes (table 2). In women, the RRs were 5.30 (95% CI 3.52 to 7.97), 10.67 (95% CI 7.43 to 15.33) and 17.09 (95% CI 12.11 to 24.11) across subgroups of <10, 10 to 20 and >20 cigarettes per day versus non-smoking, respectively. Corresponding RRs in men were 4.97 (95% CI 2.74 to 9.03), 8.93 (95% CI 4.90 to 16.28) and 14.61 (95% CI 8.33 to 25.59), respectively. The RRRs in these subgroups were 0.99 (95% CI 0.65 to 1.52), 1.11 (95% CI 0.75 to 1.64) and 0.94 (95% CI 0.69 to 1.30), respectively.

### Risk of lung cancer in former smokers versus never smokers

Data from 89 cohorts, including 6 006 725 individuals and 38 244 cases of lung cancer, reported on the risk of lung cancer in former smokers compared with never smokers. The age-adjusted RR of lung cancer associated with former smoking was 2.82 (95% CI 2.25 to 3.54) in women and 3.01 (95% CI 2.23 to 4.08) in men (table 2 and online supplementary efigure 4); the age-adjusted RRR was 0.88 (95% CI 0.69 to 1.14) (I^2^=64%; p<0.001) (table 2 and online supplementary efigure 5). The corresponding multiple-adjusted RRs were 3.14 (95% CI 2.45 to 4.03) in women and 3.13 (95% CI 2.06 to 4.76) in men (table 2 and online supplementary efigure 6). There was no statistical evidence that the effects of smoking cessation on risk of lung cancer differed between the sexes; the multiple-adjusted RRR was 0.89 (95% CI 0.69 to 1.13) (I^2^=69%; p<0.001) (table 2 and online supplementary efigure 7). There was no evidence that the RRR differed across various subgroup analyses (table 3).

## Discussion

In this systematic review and meta-analysis, comprising data from more than 7 million participants, 99 cohort studies and over 50 000 incident cases of lung cancer, there was no evidence for a difference in the risk of smoking-related lung cancer in women compared with men. This was true across a range of subgroup and sensitivity analyses. However, as smoking prevalence and intensity were higher in men compared with women in most studies included in this analysis, there may yet be an unrealised sex difference in the risk of smoking-related lung cancer that will only become fully manifest as the smoking epidemic reaches full maturity in women.^2^

The sevenfold higher RRs of lung cancer associated with smoking found in the present meta-analysis are considerably smaller than the 20-fold increased risks reported in the Million Women’s Study 13 and the British Doctors Study.^14^ Both of these studies had the advantage of capturing smoking-related risks in populations that had smoked for long enough for the effects to become fully manifest, highlighting the importance of taking into consideration the stage of the tobacco epidemic in each sex. The lack of any appreciable sex difference in the RRs of lung cancer is surprising given men’s greater cumulative exposure to smoking, in most populations, compared with women. In addition, men have a greater exposure to other risk factors for lung cancer including occupational carcinogens.^15^ Men also smoke more cigars and pipes,^3^ take longer puffs of longer duration and leave shorter butts compared with women.^4^ Hence, it may be reasonable to surmise that the RR estimates of smoking-related lung cancer in women may eventually exceed those of men, once cumulative exposure to smoking in women is comparable to that in men. In a previous meta-analysis, using similar methodology, we found that smoking conferred a 25% greater RR of coronary heart disease (CHD) in women than in men. Two possible explanations for why a similar pattern is not observed for lung cancer are that, first, the lag-time between smoking and CHD is considerably shorter than for lung cancer,^16^ and second the pathways by which smoking increases risk are different between CHD and lung cancer.

Although not assessed in this analysis, evidence suggests that there are sex differences in the pattern of lung cancer among never smokers, with a higher prevalence of lung cancer among never-smoking women than never-smoking men.^17 18^ A US study among 500000 people found a 30% higher incidence of lung cancer in women never smokers compared with men never smokers.^19^ An Australian study found the proportion of patients with lung cancer who had never smoked was approximately 18% in women and 3% of men.^20^ The reasons for this sex difference are not clear, but women may have increased exposure to environmental tobacco smoke^21^ or other environmental carcinogens such as indoor air pollution^22^ or sex-related differences in the metabolism of environmental carcinogens. The possibility of greater exposure to environmental tobacco smoke and other environmental carcinogens in women compared with men could have resulted in a greater underestimation of the association between smoking and lung cancer in women than men. This, in turn, could have impacted the sex difference in risk of smoking-related lung cancer reported in this study.

Our study has several strengths including restriction to cohort studies which provide more robust evidence of the associations compared with case–control studies. Differences between case–control and cohort studies may also explain why a previous meta-analysis of case–control studies (which included only three cohort studies) showed a higher RR of lung cancer in men compared with women.^10^ Other strengths to our study include an update of findings to include studies published after 1999,^11^ with supplementation of published literature with IPD from three established population databases. We have also performed a range of prespecified sensitivity analyses and several subgroup analyses which were not performed in previous meta-analyses. Our results were consistent across regions and irrespective of the women-to-men smoking ratio, suggesting that underestimation of the association of smoking and lung cancer in women due to sex differences in smoking prevalence and under-reporting of smoking is unlikely. This is especially relevant for parts of Asia where the prevalence of smoking in women is typically <10% and where smoking among women remains relatively socially unacceptable. As the up-take of smoking continues among women in countries where significant sex differences in smoking prevalence exist, the sex-specific risks of lung cancer due to smoking may become further apparent. This is also true for Western countries where differences in prevalence between women and men have reduced substantially over time, with prevalence of smoking in younger cohorts of women and men approaching unity.^23^ The limitations of this study include heterogeneity across studies in study design, study population, verification of smoking status and outcome ascertainment. Assessment of smoking status differed across studies and was generally self-reported, which may have introduced measurement error.^24^ Notably, compared with men, women are more likely to under-report smoking status, and under-reporting is especially prevalent in countries where smoking among women is not culturally acceptable.^25^ The lack of standardisation across studies in how smoking status was obtained, including how smoking dose and duration were measured is also a major limitation. In addition, there was insufficient data available to examine whether there were sex differences in the impact of age at smoking initiation and smoking duration on the risk of lung cancer. The reference group of non-smokers in our analysis of current smoking was composed of former and never smokers which may inflate the risks of smoking-related lung cancer risk among non-smokers. However, we have also examined former smoking compared with never smoking and demonstrated no appreciable sex differences in the risks of smoking-related lung cancer in this group, which provides some evidence that the inclusion of former smokers in the reference category is unlikely to have biased the sex difference in our main analysis. We quantified sex differences in the risk of lung cancer associated with smoking based on RRs rather than absolute risks. This might introduce a statistical artefact, in which the generally higher absolute risk for lung cancer in men, and the same risk difference subsequent to smoking in each sex, would translate to a greater RR in women than men. However, our previous meta-analyses on risk factors for cardiovascular diseases demonstrated that sex differences in RRs are not inevitable,^26^ despite differences in absolute risks. Compared with absolute risks, RRs are more stable across populations with different background risks, which makes them suitable for meta-analyses. In addition, RRs are reported much more commonly than absolute risks. In our review, no studies reported adjusted absolute risks, with standard errors, that allow for meta-analyses. We, therefore, believe that use of RRs in the present analysis is appropriate. In addition, while we have aimed to assess study quality using the widely accepted and used NOS, the value and contribution of quality assessment scales such as this to systematic reviews and meta-analyses continues to be debated.^27–29^ Finally, there are differences between men and women in histological subtypes of lung cancer. Adenocarcinoma is more common in women and squamous cell carcinoma is more common in men.^30^ Smoking is more strongly associated with squamous cell carcinoma than adenocarcinoma.^30^ Few studies reported the sex-specific association of smoking with histological subtypes of cancer, which precluded the examination of sex differences in the association of smoking-related lung cancer subtypes and this remains an important limitation of our review.^30^ Further studies of the smoking-related risks of lung cancer in women and men are required as the smoking epidemic reaches its full maturity in women. Given the later up-take of smoking in women, studies which allow sufficient lag time for lung cancer to develop are essential. In addition, reducing under-reporting of smoking in women, using standardised and robust methods for the ascertainment of smoking status and smoking behaviours and more extensive measurement and adjustment for confounders which differ by sex (such as exposure to environmental tobacco smoke) is also important for future work, as well as examination of histological subtypes of lung cancer which was not possible in this review.

In conclusion, this meta-analysis, summarising all available literature to date, shows that the effect of smoking on risk of lung cancer is similar in women and men. However, these data may yet underestimate the true RR of smoking-related lung cancer in women, given later uptake and lower intensity of smoking in women. Although strides have been made in reducing smoking rates particularly in high-income countries, continuing efforts to measure the effects of smoking on disease outcomes are required, as the smoking epidemic has not yet reached its global peak, particularly among women. In addition, tobacco control programmes that dissuade both sexes from smoking but which also encourage individuals to quit remain a priority.

## Supplementary Material

Reviewer comments

Author's manuscript

## References

[1] Institute for Health Metrics and Evaluation. Global burden of disease 2015. 2015 http://vizhub.healthdata.org/gbd-compare/#

[2] Reitsma MB, Fullman N, Ng M, et al Smoking prevalence and attributable disease burden in 195 countries and territories, 1990-2015: a systematic analysis from the global burden of disease study 2015. Lancet 2017;389:1885–906. 10.1016/S0140-6736(17)30819-X 28390697 PMC5439023

[3] Nelson DE, Davis RM, Chrismon JH, et al Pipe smoking in the United States, 1965-1991: prevalence and attributable mortality. Prev Med 1996;25:91–9. 10.1006/pmed.1996.9999 8860273

[4] Melikian AA, Djordjevic MV, Hosey J, et al Gender differences relative to smoking behavior and emissions of toxins from mainstream cigarette smoke. Nicotine Tob Res 2007;9:377–87. 10.1080/14622200701188836 17365769

[5] Becklake MR, Kauffmann F Gender differences in airway behaviour over the human life span. Thorax 1999;54:1119–38. 10.1136/thx.54.12.1119 10567633 PMC1763756

[6] Huxley RR, Woodward M Cigarette smoking as a risk factor for coronary heart disease in women compared with men: a systematic review and meta-analysis of prospective cohort studies. Lancet 2011;378:1297–305. 10.1016/S0140-6736(11)60781-2 21839503

[7] Thun MJ, Carter BD, Feskanich D, et al 50-year trends in smoking-related mortality in the United States. N Engl J Med 2013;368:351–64. 10.1056/NEJMsa1211127 23343064 PMC3632080

[8] Yun YD, Back JH, Ghang H, et al Hazard ratio of smoking on lung cancer in korea according to histological type and gender. Lung 2016;194:281–9. 10.1007/s00408-015-9836-1 26718701

[9] Powell HA, Iyen-Omofoman B, Hubbard RB, et al The association between smoking quantity and lung cancer in men and women. Chest 2013;143:123–9. 10.1378/chest.12-1068 22797799

[10] Yu Y, Liu H, Zheng S, et al Gender susceptibility for cigarette smoking-attributable lung cancer: a systematic review and meta-analysis. Lung Cancer 2014;85:351–60. 10.1016/j.lungcan.2014.07.004 25064415

[11] Lee PN, Forey BA, Coombs KJ Systematic review with meta-analysis of the epidemiological evidence in the 1900s relating smoking to lung cancer. BMC Cancer 2012;12:385 10.1186/1471-2407-12-385 22943444 PMC3505152

[12] W G, S B, Oc D, et al The Newcastle-Ottawa Scale (NOS) for assessing the quality of nonrandomised studies in meta-analyses. Ottawa: Ottawa Hospital Research Institute, 2012.

[13] Pirie K, Peto R, Reeves GK, et al The 21st century hazards of smoking and benefits of stopping: a prospective study of one million women in the UK. Lancet 2013;381:133–41. 10.1016/S0140-6736(12)61720-6 23107252 PMC3547248

[14] Doll R, Peto R, Boreham J, et al Mortality in relation to smoking: 50 years' observations on male British doctors. BMJ 2004;328:1519 10.1136/bmj.38142.554479.AE 15213107 PMC437139

[15] Olsson AC, Gustavsson P, Zaridze D, et al Lung cancer risk attributable to occupational exposures in a multicenter case-control study in Central and Eastern Europe. J Occup Environ Med 2011;53:1262–7. 10.1097/JOM.0b013e318234e2d2 22068130

[16] Ockene JK, Kuller LH, Svendsen KH, et al The relationship of smoking cessation to coronary heart disease and lung cancer in the Multiple Risk Factor Intervention Trial (MRFIT). Am J Public Health 1990;80:954–8. 10.2105/AJPH.80.8.954 2368857 PMC1404774

[17] Zang EA, Wynder EL Differences in lung cancer risk between men and women: examination of the evidence. J Natl Cancer Inst 1996;88:183–92. 10.1093/jnci/88.3-4.183 8632492

[18] Thun MJ, Hannan LM, Adams-Campbell LL, et al Lung cancer occurrence in never-smokers: an analysis of 13 cohorts and 22 cancer registry studies. PLoS Med 2008;5:e185 10.1371/journal.pmed.0050185 18788891 PMC2531137

[19] Freedman ND, Leitzmann MF, Hollenbeck AR, et al Cigarette smoking and subsequent risk of lung cancer in men and women: analysis of a prospective cohort study. Lancet Oncol 2008;9:649–56. 10.1016/S1470-2045(08)70154-2 18556244 PMC2601691

[20] Mitchell PL, Thursfield VJ, Ball DL, et al Lung cancer in Victoria: are we making progress? Med J Aust 2013;199:674–9. 10.5694/mja13.10331 24237097

[21] Sisti J, Boffetta P What proportion of lung cancer in never-smokers can be attributed to known risk factors? Int J Cancer 2012;131:265–75. 10.1002/ijc.27477 22322343 PMC3359408

[22] Hosgood HD, Boffetta P, Greenland S, et al In-home coal and wood use and lung cancer risk: a pooled analysis of the International Lung Cancer Consortium. Environ Health Perspect 2010;118:1743–7. 10.1289/ehp.1002217 20846923 PMC3002194

[23] Peters SA, Huxley RR, Woodward M Do smoking habits differ between women and men in contemporary Western populations? Evidence from half a million people in the UK Biobank study. BMJ Open 2014;4:e005663 10.1136/bmjopen-2014-005663 PMC4281541 25550291

[24] Patrick DL, Cheadle A, Thompson DC, et al The validity of self-reported smoking: a review and meta-analysis. Am J Public Health 1994;84:1086–93. 10.2105/AJPH.84.7.1086 8017530 PMC1614767

[25] Jung-Choi KH, Khang YH, Cho HJ Hidden female smokers in Asia: a comparison of self-reported with cotinine-verified smoking prevalence rates in representative national data from an Asian population. Tob Control 2012;21:536–42. 10.1136/tobaccocontrol-2011-050012 21972062

[26] Huxley RR, Peters SA, Mishra GD, et al Risk of all-cause mortality and vascular events in women versus men with type 1 diabetes: a systematic review and meta-analysis. Lancet Diabetes Endocrinol 2015;3:198–206. 10.1016/S2213-8587(14)70248-7 25660575

[27] Katikireddi SV, Egan M, Petticrew M How do systematic reviews incorporate risk of bias assessments into the synthesis of evidence? A methodological study. J Epidemiol Community Health 2015;69:189–95. 10.1136/jech-2014-204711 25481532 PMC4316857

[28] Viswanathan M, Berkman ND, Dryden DM, et al Assessing risk of bias and confounding in observational studies of interventions or exposures: further development of the RTI item bank [Internet]. Rockville (MD): Agency for Healthcare Research and Quality (US), 2013. 24006553

[29] Sanderson S, Tatt ID, Higgins JP Tools for assessing quality and susceptibility to bias in observational studies in epidemiology: a systematic review and annotated bibliography. Int J Epidemiol 2007;36:666–76. 10.1093/ije/dym018 17470488

[30] Devesa SS, Bray F, Vizcaino AP, et al International lung cancer trends by histologic type: male:female differences diminishing and adenocarcinoma rates rising. Int J Cancer 2005;117:294–9. 10.1002/ijc.21183 15900604

[31] Huxley R, Jamrozik K, Lam TH, et al Impact of smoking and smoking cessation on lung cancer mortality in the Asia-Pacific region. Am J Epidemiol 2007;165:1280–6. 10.1093/aje/kwm002 17369610

[32] Prizment AE, Yatsuya H, Lutsey PL, et al Smoking behavior and lung cancer in a biracial cohort: the atherosclerosis risk in communities study. Am J Prev Med 2014;46:624–32. 10.1016/j.amepre.2014.01.017 24842739 PMC4030495

[33] Chen Z, Peto R, Zhou M, et al Contrasting male and female trends in tobacco-attributed mortality in China: evidence from successive nationwide prospective cohort studies. Lancet 2015;386:1447–56. 10.1016/S0140-6736(15)00340-2 26466050 PMC4691901

[34] Chen ZM, Peto R, Iona A, et al Emerging tobacco-related cancer risks in China: a nationwide, prospective study of 0.5 million adults. Cancer 2015;121(Suppl 17):3097–106. 10.1002/cncr.29560 26331816 PMC4584499

[35] Gu D, Kelly TN, Wu X, et al Mortality attributable to smoking in China. N Engl J Med 2009;360:150–9. 10.1056/NEJMsa0802902 19129528

[36] Prescott E, Osier M, Rein HO, et al Gender and smoking-related risk of lung cancer. Epidemiology 1998;9:79–83. 10.1097/00001648-199801000-00016 9430273

[37] Marugame T, Sobue T, Satoh H, et al Lung cancer death rates by smoking status: comparison of the three-prefecture cohort study in Japan to the Cancer Prevention Study II in the USA. Cancer Sci 2005;96:120–6. 10.1111/j.1349-7006.2005.00013.x 15723657 PMC11158599

[38] Lam TH, Li ZB, Ho SY, et al Smoking, quitting and mortality in an elderly cohort of 56,000 Hong Kong Chinese. Tob Control 2007;16:182–9. 10.1136/tc.2006.019505 17565138 PMC2598507

[39] Lam TH, Xu L, Schooling CM, et al Smoking and mortality in a prospective cohort study of elderly Chinese in Hong Kong. Addiction 2015;110:502–10. 10.1111/add.12776 25331629

[40] Agudo A, Bonet C, Travier N, et al Impact of cigarette smoking on cancer risk in the European prospective investigation into cancer and nutrition study. J Clin Oncol 2012;30:4550–7. 10.1200/JCO.2011.41.0183 23169508

[41] Sobue T, Yamamoto S, Hara M, et al Cigarette smoking and subsequent risk of lung cancer by histologic type in middle-aged Japanese men and women: the JPHC study. Int J Cancer 2002;99:245–51. 10.1002/ijc.10308 11979440

[42] Akiba S, Hirayama T Cigarette smoking and cancer mortality risk in Japanese men and women–results from reanalysis of the six-prefecture cohort study data. Environ Health Perspect 1990;87:19. 2269225 10.1289/ehp.908719 PMC1567850

[43] Katanoda K, Marugame T, Saika K, et al Population attributable fraction of mortality associated with tobacco smoking in Japan: a pooled analysis of three large-scale cohort studies. J Epidemiol 2008;18:251–64. 10.2188/jea.JE2007429 19075498 PMC4771610

[44] Jee SH, Samet JM, Ohrr H, et al Smoking and cancer risk in Korean men and women. Cancer Causes Control 2004;15:341–8. 10.1023/B:CACO.0000027481.48153.97 15141135

[45] Ekberg-Aronsson M, Nilsson PM, Nilsson JA, et al Mortality risks among heavy-smokers with special reference to women: a long-term follow-up of an urban population. Eur J Epidemiol 2007;22:301–9. 10.1007/s10654-007-9120-7 17534729

[46] Engeland A, Haldorsen T, Andersen A, et al The impact of smoking habits on lung cancer risk: 28 years' observation of 26,000 Norwegian men and women. Cancer Causes Control 1996;7:366–76. 10.1007/BF00052943 8734831

[47] Blakely T, Barendregt JJ, Foster RH, et al The association of active smoking with multiple cancers: national census-cancer registry cohorts with quantitative bias analysis. Cancer Causes Control 2013;24:1243–55. 10.1007/s10552-013-0204-2 23580085

[48] Jha P, Ramasundarahettige C, Landsman V, et al 21st-century hazards of smoking and benefits of cessation in the United States. N Engl J Med 2013;368:341–50. 10.1056/NEJMsa1211128 23343063

[49] Freedman ND, Abnet CC, Caporaso NE, et al Impact of changing US cigarette smoking patterns on incident cancer: risks of 20 smoking-related cancers among the women and men of the NIH-AARP cohort. Int J Epidemiol 2016;45:846–56. 10.1093/ije/dyv175 26411408 PMC5005940

[50] Bjartveit K, Tverdal A Health consequences of sustained smoking cessation. Tob Control 2009;18:197–205. 10.1136/tc.2008.026898 19228666

[51] Bjartveit K, Tverdal A Health consequences of smoking 1-4 cigarettes per day. Tob Control 2005;14:315–20. 10.1136/tc.2005.011932 16183982 PMC1748107

[52] Vollset SE, Tverdal A, Gjessing HK Smoking and deaths between 40 and 70 years of age in women and men. Ann Intern Med 2006;144:381–9. 10.7326/0003-4819-144-6-200603210-00004 16549850

[53] Marang-van de Mheen PJ, Smith GD, Hart CL, et al Are women more sensitive to smoking than men? Findings from the Renfrew and Paisley study. Int J Epidemiol 2001;30:787–92. 10.1093/ije/30.4.787 11511603

[54] Tulinius H, Sigfússon N, Sigvaldason H, et al Risk factors for malignant diseases: a cohort study on a population of 22,946 Icelanders. Cancer Epidemiol Biomarkers Prev 1997;6:863–73. 9367058

[55] Wang YY, Zhang W, Li HL, et al Population attributable risks of cigarette smoking for deaths of all causes, all cancers and other chronic diseases among adults aged 40-74 years in urban Shanghai, China. Chin J Cancer Res 2015;27:59 10.3978/j.issn.1000-9604.2015.02.08 25717227 PMC4329177

[56] Shankar A, Yuan JM, Koh WP, et al Morbidity and mortality in relation to smoking among women and men of Chinese ethnicity: the Singapore Chinese Health Study. Eur J Cancer 2008;44:100–9. 10.1016/j.ejca.2007.10.015 18006298 PMC2259462

[57] Nilsson S, Carstensen JM, Pershagen G Mortality among male and female smokers in Sweden: a 33 year follow up. J Epidemiol Community Health 2001;55:825–30. 10.1136/jech.55.11.825 11604439 PMC1763319

[58] Nordlund LA, Carstensen JM, Pershagen G Are male and female smokers at equal risk of smoking-related cancer: evidence from a Swedish prospective study. Scand J Public Health 1999;27:56–62. 10.1177/14034948990270010301 10847673

[59] Meyer J, Rohrmann S, Bopp M, et al Impact of smoking and excess body weight on overall and site-specific cancer mortality risk. Cancer Epidemiol Biomarkers Prev 2015;24:1516–22. 10.1158/1055-9965.EPI-15-0415 26215293

[60] Wen CP, Tsai SP, Chen CJ, et al The mortality risks of smokers in Taiwan: Part I: cause-specific mortality. Prev Med 2004;39:528–35. 10.1016/j.ypmed.2004.02.010 15313092

