# Supplementary material for: Smoking as a risk factor for lung cancer in women and men: a systematic review and meta-analysis
Source: BMJ Open. 2018 Oct 3;8(10):e021611. doi: 10.1136/bmjopen-2018-021611 (PMC6194454; doi:10.1136/bmjopen-2018-021611)
Supplement: Supplementary file 1 [file bmjopen-2018-021611supp001.pdf]

Supplementary Material

Smoking as a risk factor for lung cancer in women and men: a systematic review and meta-analysis

eAppendix 1: MOOSE checklist

eAppendix 2: Details of search strategy

eAppendix3: Quality assessment checklist

eTable 1: Quality assessment of the included studies

eFigure 1: Age-adjusted relative risk for incident lung cancer in women and men, comparing current smokers to non-smokers

eFigure 2: Age-adjusted women-to-men ratio of relative risks for incident lung cancer, comparing current smokers to non-smokers

eFigure 3: Contour funnel plots with pseudo 95% confidence limits for the ratio of relative risks associated with current and former smoking

eFigure 4: Age-adjusted relative risk for incident lung cancer in women and men, comparing former smokers to never smokers

eFigure 5: Age-adjusted women-to-men ratio of relative risks for incident lung cancer, comparing former smokers to never smokers

eFigure 6: Multiple-adjusted relative risk for incident lung cancer in women and men, comparing former smokers to never smokers

eFigure 7: Multiple-adjusted women-to-men ratio of relative risks for incident lung cancer, comparing former smokers to never smokers

eAppendix 1: MOOSE checklist

Item No	Recommendation	Reported on Page No
Reporting of background should include		
1	Problem definition	4
2	Hypothesis statement	4
3	Description of study outcome(s)	4
4	Type of exposure or intervention used	4
5	Type of study designs used	4
6	Study population	4
Reporting of search strategy should include		
7	Qualifications of searchers (eg, librarians and investigators)	6
8	Search strategy, including time period included in the synthesis and key words	6
9	Effort to include all available studies, including contact with authors	6
10	Databases and registries searched	6
11	Search software used, name and version, including special features used (eg, explosion)	6
12	Use of hand searching (eg, reference lists of obtained articles)	6
13	List of citations located and those excluded, including justification	13
14	Method of addressing articles published in languages other than English	6
15	Method of handling abstracts and unpublished studies	6
16	Description of any contact with authors	6
Reporting of methods should include		
17	Description of relevance or appropriateness of studies assembled for assessing the hypothesis to be tested	6&7
18	Rationale for the selection and coding of data (eg, sound clinical principles or convenience)	6&7
19	Documentation of how data were classified and coded (eg, multiple raters, blinding and interrater reliability)	6&7

20	Assessment of confounding (eg, comparability of cases and controls in studies where appropriate)	6&7
21	Assessment of study quality, including blinding of quality assessors, stratification or regression on possible predictors of study results	6
22	Assessment of heterogeneity	7
23	Description of statistical methods (eg, complete description of fixed or random effects models, justification of whether the chosen models account for predictors of study results, dose-response models, or cumulative meta-analysis) in sufficient detail to be replicated	7
24	Provision of appropriate tables and graphics	13-18
Reporting of results should include		
25	Graphic summarizing individual study estimates and overall estimate	14&15
26	Table giving descriptive information for each study included	12
27	Results of sensitivity testing (eg, subgroup analysis)	18
28	Indication of statistical uncertainty of findings	8&9

Item No	Recommendation	Reported on Page No
Reporting of discussion should include		
29	Quantitative assessment of bias (eg, publication bias)	eTable7
30	Justification for exclusion (eg, exclusion of non-English language citations)	6
31	Assessment of quality of included studies	eTable1
Reporting of conclusions should include		
32	Consideration of alternative explanations for observed results	10
33	Generalization of the conclusions (ie, appropriate for the data presented and within the domain of the literature review)	10
34	Guidelines for future research	11
35	Disclosure of funding source	3

From: Stroup DF, Berlin JA, Morton SC, et al, for the Meta-analysis Of Observational Studies in Epidemiology (MOOSE) Group. Meta-analysis of Observational Studies in Epidemiology. A Proposal for Reporting. *JAMA*. 2000;283(15):2008-2012. doi: 10.1001/jama.283.15.2008.

Transcribed from the original paper within the NEUROSURGERY® Editorial Office, Atlanta, GA, United Sates. August 2012.

Appendix 2: Details of search strategy

Date of search: 15/04/2016

EMBASE (5,545 hits)
(Tobacco.ti. or tobacco.ab. or *smoking/ or smoking.ti. or smoking.ab. or smoker.ti. or smoker.ab.) AND ((cancer.ti. or cancer.ab. or neoplasm.ti. or neoplasm.ab. or neoplasms.ti. or neoplasms.ab. or neoplastic.ti. or neoplastic.ab. or *cancer mortality/) AND (lung.ti or lung.ab. or pulmonary.ti. or pulmonary.ab.) or *carcinoma or *neoplasm or *lung cancer) AND (male.ti. or male.ab. or men.ab. or men.ti. or female.ti. or female.ab. or women.ab. or women.ti or sex.ti. or sex.ab. or gender.ti. or gender.ab. or *gender or *sex or *sex difference) AND (*cohort analysis/ or cohort.ti. or cohort.ab. or follow-up.ti. or follow-up.ab. or *follow up/ or prospective.ti. or prospective.ab. or retrospective.ti. or retrospective.ab. or longitudinal.ti. or longitudinal.ab. or *population research/ or *register or *health survey/ or *risk/ or *attributable risk/ or *population risk/ or risk ratio.ab. or risk.ti. or risk.ab. or *hazard ratio/ or rate ratio.ab.)

HUMANS, SINCE 01/01/1999

Pubmed/Medline (4,588 hits)
(((((("tobacco"[MeSH Terms] OR "tobacco"[ti/ab] OR "smoking"[MeSH Terms] OR "smoker*"[ti/ab] OR "smoking"[ti/ab])) AND ("mortality"[ti/ab] OR death[ti/ab] OR "cancer"[ti/ab] OR "neoplasm"[ti/ab] OR "neoplasms"[ti/ab] OR "neoplastic"[ti/ab] OR "neoplasms"[MeSH Terms] OR "Smoking/mortality*"[MeSH Terms])) AND (("male"[mesh] OR "male"[all fields] OR "men"[mesh] OR "men"[all fields]) AND ("Female"[MeSH] OR "female"[all fields] OR "women"[mesh] OR "women"[all fields]) OR ("sex"[MeSH] OR "sex"[ti/ab] OR "gender"[ti/ab])) AND ("Cohort Studies"[Mesh] OR "Prospective Studies"[Mesh] OR "Longitudinal Studies"[Mesh] OR "cohort"[All Fields] OR "prospective"[All Fields] OR "longitudinal"[All Fields] OR "population-based"[All Fields] OR "registries"[MeSH] OR "relative risk"[all fields] OR "risk ratio"[all fields] OR "hazard ratio"[all fields] OR "rate ratio"[all fields] OR "Health Surveys"[MesH])))) Sort by: Relevance Filters: Publication date from 1999/01/01

Appendix 3: Quality assessment checklist

We used a modified version of the Newcastle Ottawa Scale (NOS) as outlined below. [12] The NOS assigns a maximum of four points for selection, two points for comparability and three points for outcome. Nine points on the NOQAS reflects the highest study quality. The criteria used to allocate these points are starred under each domain below using an asterisk (*).

Selection

S1) Representativeness of the exposed cohort
- a) Truly representative of the general population*
- b) Somewhat representative of the general population
- c) Selected group e.g. patient groups
- d) No description of the derivation of the cohort

S2) Selection of the non-exposed cohort
- a) Drawn from the same community as the exposed cohort *
- b) Drawn from a different source
- c) No description of the derivation of the non-exposed cohort

S3) Ascertainment of exposure
- a) Secure record (validated events) *
- b) Structured interview
- c) Written self-report
- d) No description

S4) Demonstration that outcome of interest was not present at start of study
- a) Yes *
- b) No

Comparability

C1) Comparability of cohorts on the basis of the design or analysis
- a) Study controls for age*
- b) Study does not control for additional factors in baseline model adjusted for age (cohort or year ok)*

Outcome

O1) Assessment of outcome
- a) Independent blind assessment or record linkage of fatal and non-fatal events*
- b) Independent blind assessment or record linkage of fatal events
- c) Self-report
- d) No description

O2) Was follow-up long enough for outcomes to occur
- a) Yes (at least 3 years) *
- b) No

O3) Adequacy of follow up of cohorts
- a) Complete follow up - all subjects accounted for *
- b) Subjects lost to follow up unlikely to introduce bias - > 10% follow up, or description provided of those lost *
- c) Follow up rate < 90% and no description of those lost
- d) No statement

eTable 1: Quality assessment of the 29 included studies

	Selection	Comparability	Ascertainment of outcome	Overall quality score
**APCSC (ANZ)**	2	1	3	6
**APCSC (ASIA)**	2	1	3	6
**ARIC**	4	1	3	8
**China Kadoorie Biobank**	3	1	3	7
**China National Hypertension Survey**	3	1	3	7
**Copenhagen Cohort Studies**	3	1	2	6
**CPS I**	3	1	2	6
**CPS II**	2	1	2	5
**EHS**	3	1	3	7
**EPIC**	4	1	3	8
**JPHC, JACC, TPCS**	2	1	3	6
**Korean Cancer Prevention Study**	3	1	2	6
**Korean National Health Insurance Service**	3	1	3	7
**Malmo Preventive Project**	2	1	2	5
**Migrant study**	3	1	3	7
**New Zealand Census 1981**	3	1	3	7
**New Zealand Census 1996**	3	1	3	7
**NHANES III**	3	1	3	7
**NHIS**	3	1	3	7
**NIH-AARP**	3	1	3	7
**Norwegian Counties Study**	2	1	3	6
**Renfrew/Paisley study**	2	1	3	6
**Reykjavik study**	3	1	3	7
**Shanghai Health Study**	3	1	3	7
**SHHEC**	2	1	3	6
**Singapore Chinese Health Study**	4	1	3	8
**Swedish smoking habit survey**	4	1	3	8
**Swiss National Cohort**	2	1	2	5
**Wen**	2	1	2	5

APCSC, Asia Pacific Cohort Studies Collaboration; ARIC, Atherosclerosis Risk in Communities study; CPS, Cancer Prevention Study; EHS, Elderly Health Services; EPIC, European Prospective Investigation into Cancer; JACC, Japan Collaborative Cohort Study; JPHC, Japan Public Health Centre study; NHANES III, National Health And Nutrition Examination Survey III; NHIS, National Health Interview Survey; NIH-AARP, NIH-AARP Diet and Health Study ; SHHEC, Scottish Heart Health Extended Cohort Study; TPCS, Three-Prefecture Cohort Study.

Study quality assessment was based on the nine-star NOS using pre-defined criteria namely: selection (population representativeness), comparability (Adjustment for confounders), and ascertainment of outcome. The NOS assigns a maximum of four points for selection, two points for comparability and three points for outcome. Nine points on the NOS reflects the highest study quality.

eFigure 1: Age-adjusted relative risk for incident lung cancer in women and men, comparing current smokers to non-smokers

Study	RR (95% CI)	% Weight
**Women**		
Korean Cancer Prevention Study	2.50 (2.00, 3.10)	5.53
APCSC - Asia	2.57 (1.85, 3.56)	5.39
Shanghai Health Study	2.69 (1.96, 3.68)	5.41
CPS I	2.74 (2.07, 3.62)	5.46
Wen	3.36 (1.44, 7.86)	4.23
Korean National Health Insurance Service	3.81 (3.06, 4.74)	5.53
JPHC, JACC, TPCS	3.88 (3.07, 4.90)	5.52
EHS	4.31 (3.58, 5.19)	5.57
Swedish 1963 Smoking Habit Survey	4.87 (3.58, 6.62)	5.42
Renfrew/Paisley Study	8.94 (6.87, 11.64)	5.48
Copenhagen Cohort Studies	12.24 (8.95, 16.75)	5.41
CPS II	12.62 (11.13, 14.31)	5.62
ARIC	13.13 (8.46, 20.41)	5.19
Norwegian Counties Study	16.07 (10.16, 25.43)	5.15
APCSC - ANZ	17.05 (10.86, 26.77)	5.17
Migrant Study	17.28 (10.64, 28.06)	5.10
SHHEC	22.11 (11.68, 41.86)	4.75
Reykjavik Study	22.25 (15.29, 32.39)	5.31
NHANES III	27.02 (14.41, 50.65)	4.78
**Subtotal (I-squared = 96.4%, p = 0.000)**	7.48 (5.29, 10.60)	100.00
.		
**Men**		
APCSC - Asia	2.50 (2.11, 2.96)	5.54
Wen	2.73 (2.08, 3.59)	5.43
Korean National Health Insurance Service	3.05 (2.83, 3.29)	5.59
Korean Cancer Prevention Study	4.60 (4.00, 5.30)	5.56
JPHC, JACC, TPCS	4.79 (3.88, 5.92)	5.50
Shanghai Health Study	5.50 (4.09, 7.40)	5.40
Migrant Study	5.76 (3.85, 8.64)	5.23
EHS	6.10 (5.00, 7.43)	5.51
Swedish 1963 Smoking Habit Survey	9.40 (6.53, 13.60)	5.30
CPS I	12.49 (9.80, 15.92)	5.47
SHHEC	12.91 (6.87, 24.26)	4.78
APCSC - ANZ	13.82 (8.94, 21.38)	5.18
Reykjavik Study	13.98 (9.54, 20.49)	5.27
Norwegian Counties Study	14.33 (8.60, 23.86)	5.04
Renfrew/Paisley Study	14.95 (10.46, 21.37)	5.31
Copenhagen Cohort Studies	16.03 (10.78, 23.84)	5.25
NHANES III	16.07 (8.58, 30.11)	4.79
CPS II	25.30 (21.10, 30.34)	5.53
ARIC	41.41 (18.36, 93.42)	4.35
**Subtotal (I-squared = 97.8%, p = 0.000)**	8.78 (6.13, 12.57)	100.00
.		

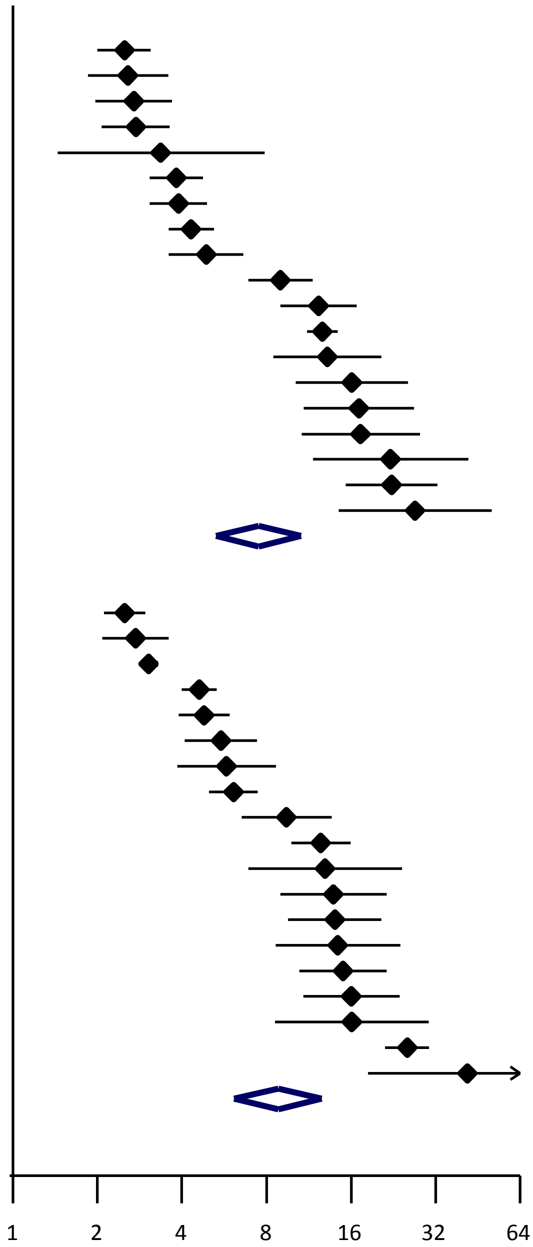

Legend: Multiple-adjusted includes anything that adjusted for more than just age. These covariates are listed in Table 1.Figures may contain less than 29 studies because we report age- and multiple-adjusted results separately. Some studies only contributed age-adjusted results whereas others only provided multiple-adjusted results. However, the count of unique studies that contributed to at least one of these analyses is 29.

eFigure 2: Age-adjusted women-to-men ratio of relative risks for incident lung cancer, comparing current smokers to non-smokers

Study	RRR (95% CI)	% Weight
CPS I	0.22 (0.15, 0.32)	5.94
ARIC	0.32 (0.13, 0.80)	3.61
Shanghai Health Study	0.49 (0.32, 0.75)	5.69
CPS II	0.50 (0.40, 0.62)	6.45
Swedish 1963 Smoking Habit Survey	0.52 (0.32, 0.84)	5.49
Korean Cancer Prevention Study	0.54 (0.42, 0.71)	6.33
Renfrew/Paisley Study	0.60 (0.38, 0.93)	5.64
EHS	0.71 (0.54, 0.93)	6.30
Copenhagen Cohort Studies	0.76 (0.46, 1.27)	5.37
JPHC, JACC, TPCS	0.81 (0.59, 1.11)	6.15
APCSC - Asia	1.03 (0.71, 1.49)	5.95
Norwegian Counties Study	1.12 (0.56, 2.23)	4.57
Wen	1.23 (0.50, 3.00)	3.74
APCSC - ANZ	1.23 (0.66, 2.31)	4.83
Korean National Health Insurance Service	1.25 (0.99, 1.58)	6.42
Reykjavik Study	1.59 (0.93, 2.72)	5.24
NHANES III	1.68 (0.69, 4.09)	3.75
SHHEC	1.71 (0.70, 4.20)	3.72
Migrant Study	3.00 (1.60, 5.64)	4.81
**Overall (I-squared = 86.2%, p = 0.000)**	0.81 (0.62, 1.04)	100.00

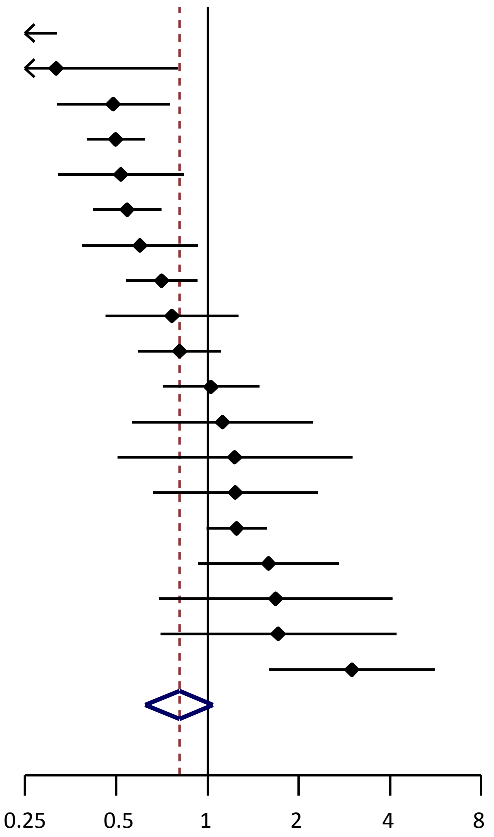

Legend: Multiple-adjusted includes anything that adjusted for more than just age. These covariates are listed in Table 1.Figures may contain less than 29 studies because we report age- and multiple-adjusted results separately. Some studies only contributed age-adjusted results whereas others only provided multiple-adjusted results. However, the count of unique studies that contributed to at least one of these analyses is 29.

eFigure 3: Contour funnel plots with pseudo 95% confidence limits for the ratio of relative risks associated with current and former smoking

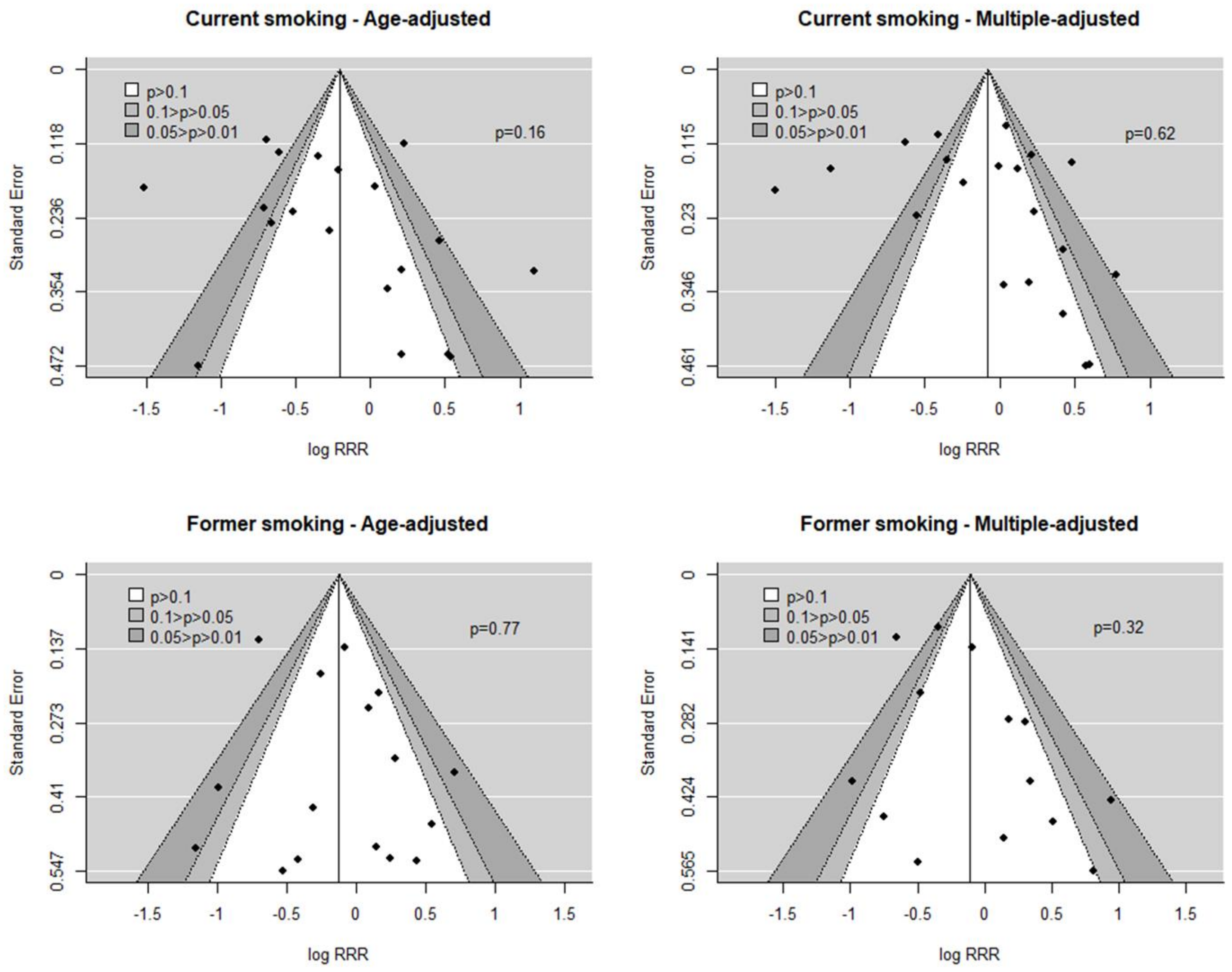

P value from Begg’s test = P=0.75

eFigure 4: Age-adjusted relative risk for incident lung cancer in women and men, comparing former smokers to never smokers

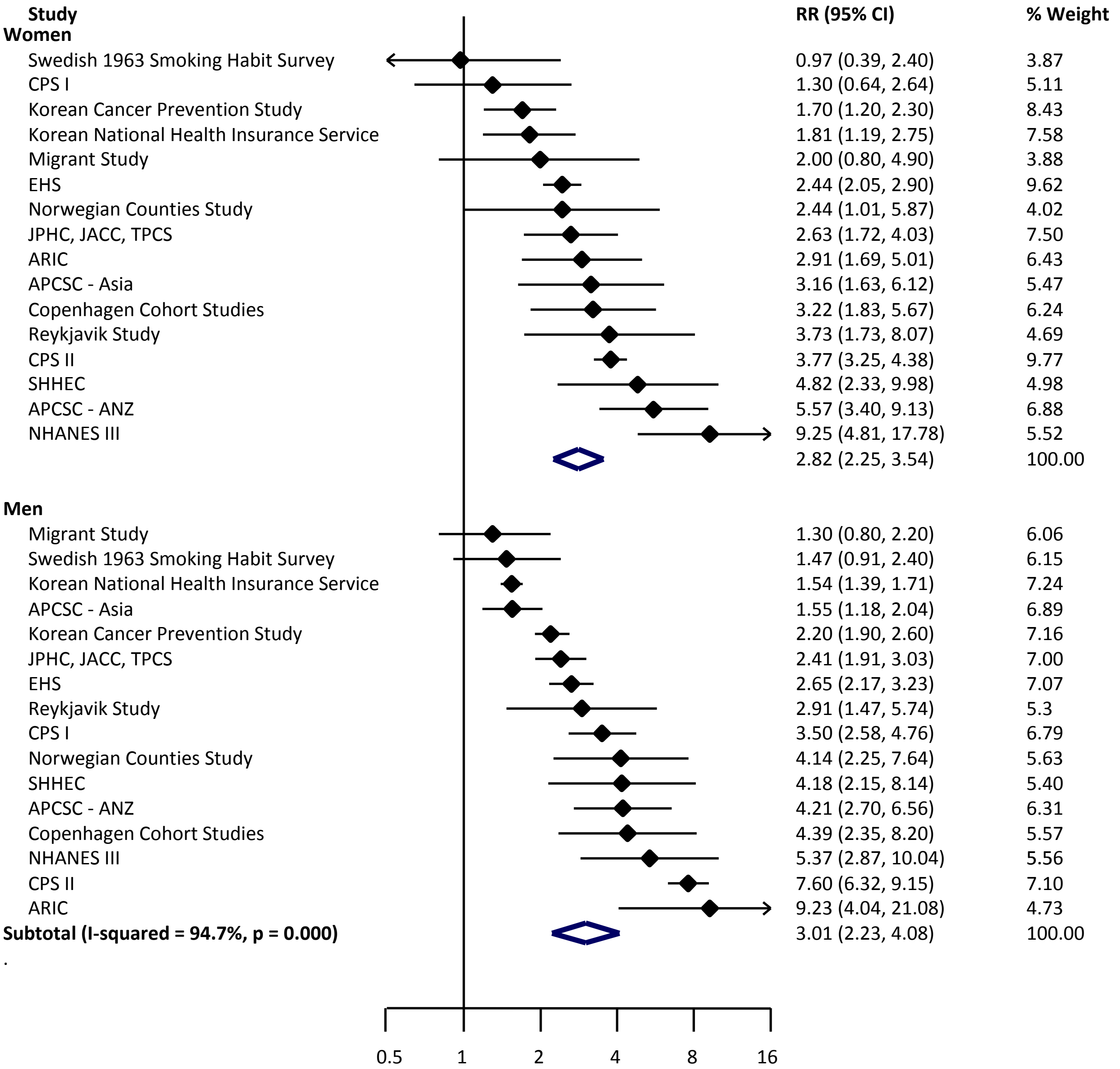

Legend: Multiple-adjusted includes anything that adjusted for more than just age. These covariates are listed in Table 1.Figures may contain less than 29 studies because we report age- and multiple-adjusted results separately. Some studies only contributed age-adjusted results whereas others only provided multiple-adjusted results. However, the count of unique studies that contributed to at least one of these analyses is 29.

eFigure 5: Age-adjusted women-to-men ratio of relative risks for incident lung cancer, comparing former smokers to never smokers

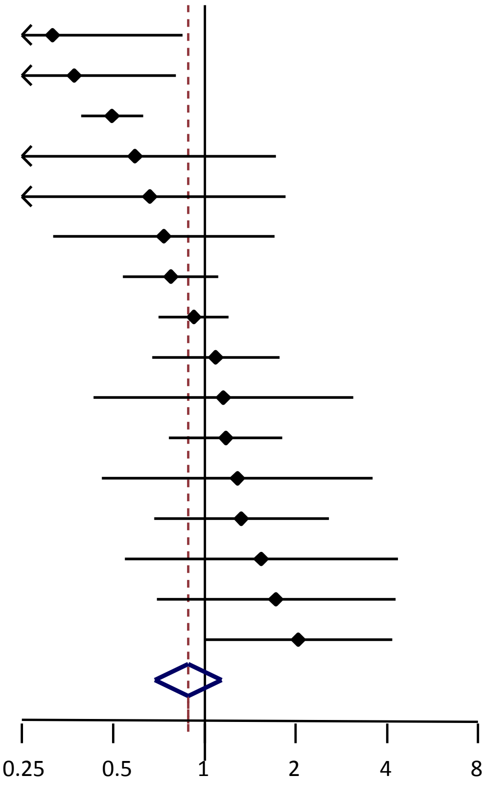

Study	RRR (95% CI)	% Weight
ARIC	0.32 (0.12, 0.85)	4.19
CPS I	0.37 (0.17, 0.80)	5.61
CPS II	0.50 (0.39, 0.63)	10.77
Norwegian Counties Study	0.59 (0.20, 1.72)	3.76
Swedish 1963 Smoking Habit Survey	0.66 (0.24, 1.85)	3.97
Copenhagen Cohort Studies	0.73 (0.32, 1.70)	5.09
Korean Cancer Prevention Study	0.77 (0.54, 1.11)	9.57
EHS	0.92 (0.71, 1.20)	10.54
JPHC, JACC, TPCS	1.09 (0.67, 1.77)	8.26
SHHEC	1.15 (0.43, 3.09)	4.20
Korean National Health Insurance Service	1.17 (0.76, 1.80)	8.84
Reykjavik Study	1.28 (0.46, 3.58)	3.98
APCSC - ANZ	1.32 (0.68, 2.57)	6.51
Migrant Study	1.54 (0.54, 4.34)	3.93
NHANES III	1.72 (0.70, 4.26)	4.68
APCSC - Asia	2.04 (1.00, 4.16)	6.07
**Overall (I-squared = 65.4%, p = 0.000)**	0.88 (0.69, 1.14)	100.00

Legend: Multiple-adjusted includes anything that adjusted for more than just age. These covariates are listed in Table 1.Figures may contain less than 29 studies because we report age- and multiple-adjusted results separately. Some studies only contributed age-adjusted results whereas others only provided multiple-adjusted results. However, the count of unique studies that contributed to at least one of these analyses is 29.

eFigure 6: Multiple-adjusted relative risk for incident lung cancer in women and men, comparing former smokers to never smokers

Study	RR (95% CI)	% Weight
**Women**		
Swiss National Cohort	1.05 (0.47, 2.34)	5.13
CPS I	1.30 (0.64, 2.64)	5.81
Korean National Health Insurance Service	1.74 (1.03, 2.96)	7.32
EHS	2.48 (2.07, 2.96)	10.36
Norwegian Counties Study	2.51 (1.04, 6.06)	4.62
Singapore Chinese Health Study	2.70 (1.70, 4.30)	7.94
Malmo Preventive Project	2.71 (1.02, 7.23)	4.07
EPIC	3.28 (2.46, 4.37)	9.56
APCSC - Asia	3.49 (1.63, 7.47)	5.41
CPS II	3.85 (3.32, 4.47)	10.53
SHHEC	4.84 (2.34, 10.02)	5.67
APCSC - ANZ	5.22 (2.91, 9.36)	6.83
NIH-AARP	5.40 (4.80, 6.20)	10.64
NHANES III	7.66 (3.92, 14.98)	6.11
**Subtotal (I-squared = 85.2%, p = 0.000)**	3.14 (2.45, 4.03)	100.00
**Men**		
Malmo Preventive Project	1.21 (0.72, 2.02)	6.87
APCSC - Asia	1.36 (0.95, 1.95)	7.26
Korean National Health Insurance Service	1.45 (1.30, 1.63)	7.62
Singapore Chinese Health Study	2.00 (1.50, 2.70)	7.39
Swiss National Cohort	2.22 (1.46, 3.38)	7.12
EHS	2.73 (2.23, 3.35)	7.53
CPS I	3.48 (2.56, 4.74)	7.36
APCSC - ANZ	3.71 (2.24, 6.14)	6.90
Norwegian Counties Study	4.13 (2.24, 7.64)	6.58
SHHEC	4.21 (2.16, 8.18)	6.42
NHANES III	4.60 (2.44, 8.66)	6.52
EPIC	5.28 (3.77, 7.38)	7.30
CPS II	7.41 (6.16, 8.91)	7.55
NIH-AARP	7.60 (6.60, 8.90)	7.59
**Subtotal (I-squared = 97.2%, p = 0.000)**	3.13 (2.06, 4.76)	100.00

.

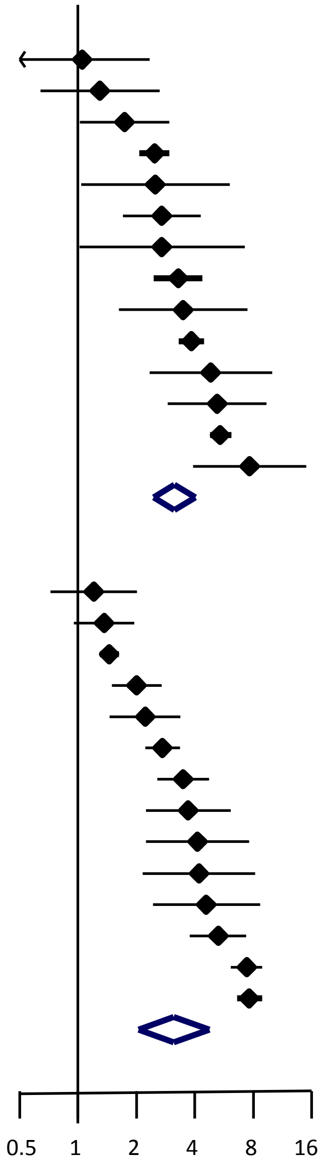

Legend: Multiple-adjusted includes anything that adjusted for more than just age. These covariates are listed in Table 1.Figures may contain less than 29 studies because we report age- and multiple-adjusted results separately. Some studies only contributed age-adjusted results whereas others only provided multiple-adjusted results. However, the count of unique studies that contributed to at least one of these analyses is 29.

eFigure 7: Multiple-adjusted women-to-men ratio of relative risks for incident lung cancer, comparing former smokers to never smokers

Study	RRR (95% CI)	% Weight
CPS I	0.37 (0.17, 0.81)	5.83
Swiss National Cohort	0.47 (0.19, 1.17)	4.81
CPS II	0.52 (0.41, 0.66)	12.01
Norwegian Counties Study	0.61 (0.21, 1.78)	3.81
EPIC	0.62 (0.40, 0.97)	9.44
NIH-AARP	0.71 (0.58, 0.87)	12.44
EHS	0.91 (0.69, 1.19)	11.62
SHHEC	1.15 (0.43, 3.08)	4.29
Korean National Health Insurance Service	1.20 (0.70, 2.06)	8.20
Singapore Chinese Health Study	1.35 (0.78, 2.34)	8.11
APCSC - ANZ	1.41 (0.65, 3.04)	5.84
NHANES III	1.67 (0.66, 4.19)	4.70
Malmo Preventive Project	2.24 (0.74, 6.77)	3.65
APCSC - Asia	2.56 (1.10, 5.94)	5.26
**Overall (I-squared = 68.9%, p = 0.000)**	0.89 (0.69, 1.13)	100.00

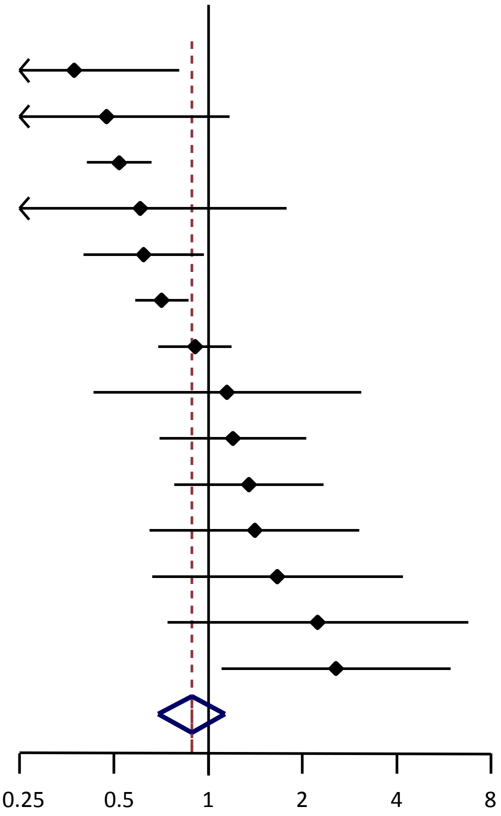

Legend: Multiple-adjusted includes anything that adjusted for more than just age. These covariates are listed in Table 1.Figures may contain less than 29 studies because we report age- and multiple-adjusted results separately. Some studies only contributed age-adjusted results whereas others only provided multiple-adjusted results. However, the count of unique studies that contributed to at least one of these analyses is 29.
