# Supplementary material for: Smoking as a risk factor for lung cancer in women and men: a systematic review and meta-analysis
Source: BMJ Open. 2018 Oct 3;8(10):e021611. doi: 10.1136/bmjopen-2018-021611 (PMC6194454; doi:10.1136/bmjopen-2018-021611)
Supplement: Author's manuscript [file bmjopen-2018-021611.draft_revisions.pdf]

BMJ Open

BMJ Open is committed to open peer review. As part of this commitment we make the peer review history of every article we publish publicly available.

When an article is published we post the peer reviewers' comments and the authors' responses online. We also post the versions of the paper that were used during peer review. These are the versions that the peer review comments apply to.

The versions of the paper that follow are the versions that were submitted during the peer review process. They are not the versions of record or the final published versions. They should not be cited or distributed as the published version of this manuscript.

BMJ Open is an open access journal and the full, final, typeset and author-corrected version of record of the manuscript is available on our site with no access controls, subscription charges or pay-per-view fees (http://bmjopen.bmj.com).

If you have any questions on BMJ Open's open peer review process please email info.bmjopen@bmj.com

BMJ Open

Smoking as a risk factor for lung cancer in women and men: a systematic review and meta-analysis

Journal:	*BMJ Open*
Manuscript ID	bmjopen-2018-021611
Article Type:	Research
Date Submitted by the Author:	15-Jan-2018
Complete List of Authors:	O'Keeffe, Linda; MRC Integrative Epidemiology Unit, Bristol Medical School; Population Health Sciences, Bristol Medical School, University of Bristol<br>Taylor, Gemma; MRC Integrative Epidemiology Unit at the University of Bristol, Bristol Medical School, University of Bristol, Bristol, UK; UK Centre for Tobacco and Alcohol Studies, School of Experimental Psychology, University of Bristol, UK<br>Huxley, Rachel; School of Public Health, Faculty of Health Sciences, Curtin University,<br>Mitchell, Paul; Olivia Newton-John Cancer and Wellness Centre, Austin Health and Olivia Newton-John Cancer Research Institute, Heidelberg, VIC 3084, Australia<br>Woodward, Mark; The George Institute for Global Health,<br>Peters, Sanne; University of Oxford, George Institute for Global Health
Keywords:	smoking, systematic review, lung cancer, sex-specific

SCHOLARONE™
Manuscripts

For peer review only

Smoking as a risk factor for lung cancer in women and men: a systematic review and meta-analysis

Linda M. O'Keeffe[1, 2*], Gemma Taylor[1, 2, 3*], Rachel R. Huxley[4], Paul Mitchell[5], Mark Woodward[6,7,8], Sanne A.E. Peters[6]

* Denotes equal contribution

1 MRC Integrative Epidemiology Unit at the University of Bristol, Bristol Medical School, University of Bristol, Bristol, UK
2 Population Health Sciences, Bristol Medical School, University of Bristol
3 UK Centre for Tobacco and Alcohol Studies, School of Experimental Psychology, University of Bristol, UK
4 School of Public Health, Faculty of Health Sciences, Curtin University, Perth, Australia
5 Olivia Newton-John Cancer and Wellness Centre, Austin Health and Olivia Newton-John Cancer Research Institute, Heidelberg, VIC 3084, Australia
6 The George Institute for Global Health, University of Oxford, Oxford, UK
7 The George Institute for Global Health, University of New South Wales, Sydney, Australia
8 Department of Epidemiology, Johns Hopkins University, Baltimore MD, USA

Correspondence: Dr. Linda O'Keeffe
MRC Integrative Epidemiology Unit at the University of Bristol, University of Bristol, Oakfield House, Oakfield Grove, BS8 2BN, United Kingdom T: +44 7534498919

Email address: lindaokeeffe1987@gmail.com

Funding: The MRC Integrative Epidemiology Unit at the University of Bristol is supported by the Medical Research Council and the University of Bristol [MC_UU_12013/6, MC_UU_12013/9]. LMOK is supported by a UK Medical Research Council Population Health Scientist fellowship (MR/M014509/1). GMJT is funded by a Cancer Research UK Postdoctoral Fellowship (C56067/A21330). SAEP is supported by a UK Medical Research Council Skills Development Fellowship (MR/P014550/1)

Data sharing statement: Technical appendix, statistical code, and dataset (of published data only) available from authors upon request.

Contributor statement: LMOK, GT, SAEP, RH and MW designed the study. LMOK and GT performed systematic searches, retrieved literature and performed data extraction. SAEP performed the analysis of the data. LMOK and GT wrote the first draft of the article. RH, MW and PM contributed important intellectual content and critical expertise and revisions to the manuscript.

Competing interest's statement: We have no competing interests to declare.

For peer review only

Abstract

Objectives: To investigate the sex-specific association between smoking and lung cancer.

Design: Systematic review and meta-analysis.

Data sources: We searched Pubmed and Embase, from January 1st, 1999 to April 15th, 2016 for cohort studies. Cohort studies before January 1st, 1999 were retrieved from previous meta-analyses. Individual participant data from three sources was also available to supplement analyses of published literature.

Eligibility criteria for selecting studies: Cohort studies reporting the sex-specific relative risk (RR) of lung cancer associated with smoking.

Results: Data from 99 cohort studies, representing seven million individuals and > 50,000 incident lung cancer cases were included. The pooled multiple-adjusted lung cancer RR was 6.99 (95% Confidence Interval (CI): 5.09, 9.59) in women and 7.33 (95% CI: 4.90, 10.96) in men. The pooled ratio of the RRs was 0.92 (95% CI: 0.72 – 1.16; I^2=89%; p<0.001), with no evidence of publication bias, or differences across major predefined participant and study subtypes. The women-to-men ratio of RRs were 0.99 (95% CI: 0.65, 1.52), 1.11 (95% CI: 0.75, 1.64), and 0.94 (95% CI: 0.69, 1.30), for light, moderate and heavy smoking respectively.

Conclusions: Smoking yields similar risks of lung cancer in women compared with men. However, these data may underestimate the true risks of smoking among women as the epidemic has not reached full maturity in women. Continued efforts to measure the sex-specific effects of smoking are required.

Strengths and Limitations

- Evidence on the sex-specific association of smoking and lung cancer were meta-analysed in over seven million participants across 99 cohort studies.
- Several subgroup analyses were performed to examine the robustness of findings across different population subgroups.
- However, the smoking epidemic is not yet fully mature in women and risks in women may still be underestimated.
- Detailed data on smoking behaviour and data on specific subtypes of lung cancer were not available.

For peer review only

Introduction

Lung cancer is the leading cause of cancer death worldwide with 1.7 million global deaths attributed to cigarette smoking in 2015. [1] Tobacco use is by far the single biggest cause of lung cancer; 55% of lung cancer deaths in women and over 70% of lung cancer deaths in men are due to smoking. [1] These global estimates, however, mask major differences in smoking prevalence in men and women across populations, with rates below 5% for women in most Asian and African countries to 40% and above for men in many parts of Asia and Eastern Europe.[2] Moreover, these estimates are predicated on the assumption that the hazards of smoking confer similar risks in women as they do in men, which may not be true given substantial physiological differences between the sexes that may exacerbate the harmful effects of tobacco, particularly for women. For example, compared with men, women have a smaller body size and smaller lung volume, which may increase their susceptibility to lung cancer at lower levels of tobacco smoke cigarette exposure. A recent meta-analysis showed that cigarette smoking confers a greater coronary hazard among women than men, which suggests the possibility that this may also be true for the risk of smoking-related lung cancer.[3]

A recent meta-analysis on the sex-specific effects of smoking on lung cancer risk did not resolve this uncertainty; although there were apparent differences in the relative risk estimates for men and women, virtually all data were from historical case-control studies and the three included prospective studies provided contradictory results.[4] A study of 50-year trends in smoking-related mortality in the US found that the relative risks of smoking-related lung cancer mortality were higher in men than women.[5] However, this sex difference was only apparent in the oldest cohorts with the longest follow-up, possibly reflecting greater cumulative tobacco exposure in men than in women. In contrast, a recent study in Korea, a population where smoking patterns continue to differ between the sexes, suggested that sex differences in the impact of smoking on lung cancer risk exist and differ by histologic subtype.[6] Analyses of a large UK primary care database showed that moderate and heavy smoking more strongly increase the risk of lung cancer in women than in men. [7]

For peer review only

In this study, we conducted a systematic review with meta-analysis of cohort studies published to date on the sex-specific effects of smoking on the risk of fatal and non-fatal lung cancer. An important *a priori* consideration is the substantial sex difference in the maturity of the smoking epidemic with men being at a more advanced stage than women in most parts of the world. This would be expected to translate into lower relative risk estimates for lung cancer in women than in men. Hence, the null hypothesis that smoking confers the same lung cancer hazard in both women in men, would be met if the ratio of the relative risks for lung cancer (women:men) was less than unity (reflecting a greater hazard in men than women). However, if the ratio of the relative risks was found to be unity (or higher) then this would suggest greater hazard associated with tobacco exposure in women than in men.

For peer review only

Methods

Search strategy

This review was conducted using a predefined protocol and in accordance to the MOOSE guidelines (**eAppendix 1**). We systematically searched PubMed and EMBASE for studies published between January 1, 1999 and April 15th, 2016 that reported on the relationship between smoking and lung cancer in men and women from a general population. The full search criteria are available in **eAppendix 2**. Articles published before January 1st , 1999 were retrieved from previous systematic reviews.[4] The reference lists of all relevant original research and review articles were scanned to capture missed studies. Two authors (LMOK and GMT) independently conducted the screening of studies and any disagreement was mediated by a third author (SAP).

Study selection

Observational cohort studies were included if they reported sex-specific relative risks (RRs), or equivalent, on the relationship between smoking and lung cancer. Studies were excluded if the variability around the point estimate was not reported, if they had not been adjusted for at least age, or if the study was performed in a population selected on the basis of prior lung cancer or another major underlying chronic disease. In case of duplicate reports from the same study, the report with the longest follow-up or the highest number of cases was included. Individual participant data (IPD) from studies available to the authors were also used; the Asia Pacific Cohort Studies Collaboration (APCSC), the National Health and Nutrition Examination Survey III (NHANES III), and the Scottish Heart Health Extended Cohort Study (SHHEC). A modified version of the Newcastle-Ottawa Quality assessment scale was used to assess the methodological quality of all included studies (Supplementary Material).

For peer review only

Statistical analyses

The primary analysis was a comparison of the sex-specific RR of lung cancer (fatal or non-fatal) in current smokers versus non-smokers (defined either as former or never smokers). For each study, we obtained the natural log of the sex-specific RRs and calculated the differences. The differences were pooled across studies using random effects meta-analysis weighted by the inverse of the variances of the log RRs, and then back-transformed to obtain the pooled women-to-men ratio of the RRs (RRR). The standard error of the log RRR was calculated as the square root of the sum of the variance of the two sex-specific log RRs for each study. RRRs were computed separately for studies with only age-adjusted estimates and then for those studies with multiple-adjusted estimates. The set of multiple adjustments made was allowed to vary by study, but had to include at least one other risk factor in addition to age. The I^2 statistic was used to estimate the percentage of variability across studies due to between-study heterogeneity. The presence of publication bias was graphically examined using funnel plots, plotting the natural log of the RRR against its standard error, and tested using Begg's test. Pre-defined subgroup analyses were conducted to obtain to the adjusted RRRs by study region (Asia or non-Asia), year of study baseline (pre- or post-1985), study endpoint (fatal only or fatal and non-fatal combined), and number of cigarettes smoked per day (>0 to 10, 10-20, >20). To include the largest number of studies available, we combined the age-and multiple-adjusted estimates, taking the maximum-adjustment set available. In secondary analyses, we obtained the sex-specific RRs and RRRs comparing former smokers to never smokers. All analyses were performed using Stata version 12.0.

For peer review only

Results

Of the 9,519 unique records that were identified through the systematic search, 227 qualified for full-text evaluation (**Figure 1**). Of these, 23 articles with information from 25 separate studies provided information about sex differences in the association between smoking and lung cancer. This database was extended with individual participant data from APCSC, NHANES III, and SHHEC.

The characteristics of the included studies are described in **Table 1**. Overall, data were available from 99 cohorts, including 7,113,303 individuals (46% women) - not accounting for two cohorts that used Census data - and at least 51,161 incident cases of lung cancer (31% women). Forty-six cohorts were from Asia (61% of the individuals), six were from the USA (28%), 27 were from Europe (10%), and ten were from Australia and New Zealand (1%). All included studies were of good quality (**eTable 1**).

For peer review only

Eighteen studies reported on the prevalence of smoking, which varied widely by study, region, and sex. The prevalence of smoking ranged from 1% to 47% in women and from 1% to 70% in men. In all but two studies, the prevalence of smoking was higher in men than women, especially in Asia where typically less than 10% of women were smokers compared with over 50% of men. Smoking cessation rates were also higher among men (7-61%) than women (<1-39%).

Risk of lung cancer in current smokers versus non-smokers

Compared with non-smoking, current smoking was associated with an age-adjusted RR of lung cancer of 7.48 (95% Confidence Interval (CI): 5.29, 10.60) in women and 8.78 (95% CI: 6.13, 12.57) in men (**eFigure 1** & **eTable 2**). The pooled age-adjusted women-to-men RRR was 0.81 (95% CI: 0.62, 1.04), with substantial between-study heterogeneity (I^2=86%; P<0.001) (**eFigure 2**). The multiple-adjusted RR of lung cancer associated with current smoking was 6.99 (95% CI: 5.09, 9.59) in women and 7.33 (95% CI: 4.90, 10.96) in men (**Figure 2**). The corresponding RRR was 0.92 (95% CI: 0.72,

1.16) and between-study heterogeneity was substantial (I^2=89%; P<0.001) (**Figure 3**). There was no evidence of publication bias (P=0.75) (**eFigure 3**).

There was no evidence for a difference in the RRR according to the women-to-men ratio of current smokers (P=0.90) or the women-to-men ratio of lung cancer incidence in the studies (P=0.64) (**Table 2**). Neither was there evidence that the findings differed by year of study baseline (P = 0.66), study endpoint (P=0.21) or study region (P=0.73) (**Table 2**). The RRR was 0.93 (95% CI: 0.72, 1.20) for studies from Asia and 0.87 (95% CI: 0.66, 1.14) for studies from USA, Europe, or Australia and New Zealand.

The risk of smoking-related lung cancer increased according to the number of cigarettes smoked per day in both sexes (**eTable 2**). In women, the RRs were 5.30 (95% CI: 3.52, 7.97), 10.67 (95% CI: 7.43, 15.33), and 17.09 (95% CI: 12.11, 24.11) across subgroups of <10, 10 to 20, and >20 cigarettes per day versus non-smoking, respectively. Corresponding RRs in men were 4.97 (95% CI: 2.74, 9.03), 8.93 (95% CI: 4.90, 16.28), and 14.61 (95% CI: 8.33, 25.59), respectively. The RRRs in these subgroups were 0.99 (95% CI: 0.65, 1.52), 1.11 (95% CI: 0.75, 1.64), and 0.94 (95% CI: 0.69, 1.30), respectively.

Risk of lung cancer in former smokers versus never smokers

Data from 89 cohorts, including 6,006,725 individuals and 38,244 cases of lung cancer, reported on the risk of lung cancer in former smokers compared with never smokers. The age-adjusted RR of lung cancer associated with former smoking was 2.82 (95% CI: 2.25, 3.54) in women and 3.01 (95% CI: 2.23, 4.08) in men (**eTable 2** & **eFigure 4**); the age-adjusted RRR was 0.88 (95% CI: 0.69, 1.14) (I^2=64%; P<0.001) (**eTable 2** & **eFigure 5**). The corresponding multiple-adjusted RRs were 3.14 (95% CI: 2.45, 4.03) in women and 3.13 (95% CI: 2.06, 4.76) in men (**eTable 2** & **eFigure6**). There was no statistical evidence that the effects of smoking cessation on risk of lung cancer differed between the

For peer review only

sexes; the multiple-adjusted RRR was 0.89 (95% CI: 0.69, 1.13) (I^2=69%; P<0.001) (**eTable 2** & **eFigure 7**). There was no evidence that the RRR differed across various subgroup analyses (**Table 2**).

For peer review only

Discussion

In this systematic review and meta-analysis, comprising data from more than seven million participants, 99 cohort studies and over 50,000 incident cases of lung cancer, there was no difference in the risk of smoking-related lung cancer in women compared with men. This was true across a range of subgroups and sensitivity analyses. However, as smoking prevalence and intensity was higher in men compared with women in most studies included in this analysis, there may yet be an unrealised sex-difference in the risk of smoking-related lung cancer that will only become fully manifest as the smoking epidemic reaches full maturity in women.

The seven-fold higher relative risks of lung cancer associated with smoking found in the present meta-analysis are considerably smaller than the 20-fold increased risks reported in the MWS[8] and the British Doctors Study.[9] Both these studies had the advantage of capturing smoking-related risks in populations that had smoked for long enough for the effects to become fully manifest, highlighting the importance of taking into consideration the stage of the tobacco epidemic in each sex. The relatively small relative risk estimates observed in this study are likely to partly reflect the heterogeneity in study populations in terms of baseline year of study, population age, prevalence of smoking, and smoking duration and intensity. However, the lack of any appreciable sex difference in the relative risk of lung cancer is surprising given men's greater cumulative exposure to smoking, in most populations, compared with women. Moreover, men have a greater exposure to other risk factors for lung cancer including occupational carcinogens [10] and smoking of cigars and pipes.[11] Men also take longer puffs of longer duration and leave shorter butts compared with women [12], thus predisposing them to greater risk of squamous cell carcinoma, compared with women who are more likely to succumb to non-squamous cell carcinoma.[13,14] Hence, it may be reasonable to surmise that the relative risk estimates of smoking-related lung cancer in women may eventually exceed those of men, once cumulative exposure to smoking in women is comparable to that in men. In a previous meta-analysis, using similar methodology, we found that smoking confers a 25% greater relative risk

For peer review only

of coronary heart disease (CHD) in women than in men. Two possible explanations for why a similar pattern is not observed for lung cancer are that, first, the lag-time of smoking associated with CHD is considerably shorter than for lung cancer[15], and second the pathways by which smoking increases risk are different between CHD and lung cancer.

Although not assessed in this analysis, evidence suggests that there are sex differences in the pattern of lung cancer among never smokers, with a higher prevalence of lung cancer among never-smoking women than never-smoking men.[16,17] A US study amongst 500,000 people found a 30% higher incidence of lung cancer in women never smokers compared with men never smokers.[18] An Australian study found the proportion of lung cancer patients who had never smoked was approximately 18% in women and 3% of men.[19] The reasons for this sex difference are not clear, but women may have increased exposure to environmental tobacco smoke[20] or other environmental carcinogens such as indoor air pollution[21], or sex-related differences in the metabolism of environmental carcinogens.

The main strengths of this meta-analysis are its size and the diversity of study populations included. We have used published literature supplemented by individual participant data from three established population databases allowing for a range of pre-specified sensitivity analyses and several subgroup analyses. Restriction to cohort studies in both men and women reduced the impact of biases of meta-analyses of single-sex and case-control studies. We performed stratified analyses based on level adjustment for confounders and smoking classification (current and former) and found no effect of these study characteristics on our primary results. Our results were consistent across regions and the women-to-men smoking ratio, suggesting that underestimation of the association of smoking with lung cancer in women due to underreporting is unlikely. This is especially relevant for parts of Asia where the prevalence of smoking in women is typically <10% and where smoking among women remains relatively socially unacceptable. Subgroup analyses based on

For peer review only

women to men lung cancer disease rate also suggested that the use of relative risk measures as compared with absolute risk is unlikely to have affected our estimates. The limitations of this study are inherent to its design and include heterogeneity across studies in study design, study population, verification of smoking status, and outcome ascertainment. Assessment of smoking status differed across studies and was generally self-reported, which may have introduced measurement error.[22] Notably, compared with men, women typically underreport smoking status, and underreporting is especially prevalent in countries where female smoking is not culturally acceptable.[23] The lack of standardisation across studies in smoking dose and duration are further limitations. In addition, there was insufficient data available to whether there were sex differences in the impact of age at smoking initiation and smoking duration on the risk of lung cancer. Furthermore, data on lung cancer subtypes were sparse and could not be assessed.

In conclusion, this meta-analysis, summarising all available literature to date, shows that the effect of smoking on risk of lung cancer is similar in women and men. However, these data may yet underestimate the true relative risk of smoking in women, given later uptake and lower intensity of smoking in women. Although strides have been made in reducing smoking rates particularly in high-income countries, continuing efforts to measure the effects of smoking on disease outcomes are required, as the smoking epidemic has not yet reached its global peak, particularly among women. In addition, tobacco control programmes that dissuade smokers of both sexes from smoking but which also encourage individuals to quit remain a priority.

For peer review only

Figure 1: Flow chart of study selection

Figure 2: Multiple-adjusted relative risk for incident lung cancer in women and men, comparing current smokers to non-smokers

Figure 3: Multiple-adjusted women-to-men ratio of relative risks for incident lung cancer, comparing current smokers to non-smokers

For peer review only

Table 1: Characteristics of included studies

Study name	Location	Age range, (y)	Baseline year	Follow-up, (years)	N (%) women	N (%) lung cancer in women	Fatal/ non-fatal	Current smoker, % W/M		Former smoker, % W/M		Maximum adjustment available
**APCSC – ANZ**[24]	ANZ	20-107	1961-93	10	87,130 (52)	501 (28)	F	7	11	11	18	Age, education, BMI
**APCSC - Asia**[24]	Asia	20-104	1989-96	7	476,755 (35)	1,275 (19)	F	2	59	<1	7	Age, education, BMI
**ARIC**[25]	USA	45-64	1987-89	20	14,610 (54)	470 (37)	F/NF	25	44	22	28	Age, race
**China Kadoorie Biobank**[26,27]	China	35-74	2004-08	7	512,891 (59)	1,953 (37)	F	3	61	1	15	Age, area, education, alcohol
**China National Hypertension Survey**[28]	China	15+	1991	8	155,131 (51)	NR	F	14	63	NR	NR	Age, education, region, HT, overweight/obesity, alcohol, PA, DM
**Copenhagen Cohort Studies**[29]	Denmark	20+	1964-92	14	30,874 (44)	867 (23)	F/NF	NR	NR	NR	NR	Age
**CPS I**	USA	55+	1959	6	518,982 (65)	1,293 (21)	F	15	40	4	17	Age, education, race
**CPS II**[30]	USA	55+	1982	6	746,485 (61)	4,957 (36)	F	18	24	21	43	Age, education, race
**EHS**[31,32]	China	65+	1998-00	11	65,510 (66)	1,096 (27)	F	4	10	8	20	Age, education, alcohol, depression, health status, social security assistance, housing type, expenditure
**EPIC**[33]	Europe	30-70	1992-00	11	441,211 (70)	2,995 (49)	F/NF	21	27	25	38	Age, education, BMI, alcohol, PA, energy intake, diet
**JPHC**[34]**, JACC, TPCS**[30,35,36]	Japan	40-79	1983-94	10	296,836 (53)	NR	F	8	54	2	25	Age
**Korean Cancer Prevention Study**[37]	Korea	30-95	1993-95	9	1,212,906 (32)	4,238 (14)	F	5	57	3	23	Age
**Korean National Health Insurance Service**[6]	Korea	20+	1998-99	10	1,355,891 (31)	6,491 (15)	F/NF	1	56	1	13	Age, BMI, alcohol, PA
**Malmo Preventive Project**[38]	Sweden	27-61	1974-92	24	33,346 (33)	436 (21)	F	36	49	19	27	Age, FEV, SES, marital status
**Migrant Study**[39]	Norway	33-72	1964-65	21	26,126 (55)	435 (23)	F	26	46	8	28	Age
**New Zealand Census 1981**[40]	New Zealand	25+	1981	5	NR	4,188 (28)	F/NF	NR	NR	NR	NR	Age, race
**New Zealand Census 1996**[40]	New Zealand	25+	1996	5	NR	4,467 (44)	F/NF	NR	NR	NR	NR	Age, race
**NHANES III**	USA	18-90	1988-94	13	20,006 (53)	320 (36)	F	21	30	17	32	Age, education, BMI
**NHIS**[41]	USA	25+	1997-04	7	202,248 (56)	1,223 (44)	F	21	26	20	29	Age, education, BMI, alcohol
**NIH-AARP**[5,42]	USA	50-71	1995-96	11	452,131 (41)	9,381 (37)	F/NF	17	13	39	61	Age, education, alcohol, ethnicity
**Norwegian Counties Study**[43-45]	Norway	20-49	1974-78	23	48,682 (48)	686 (35)	F	43	50	11	18	Age, SBP, TC, TG, PA, BMI, height, sickness leave, disability pension, family history of CHD
**Renfrew/Paisley Study**[46]	Scotland	45-64	1972-76	20	15,393 (54)	NR	F	47	59	7	25	Age
**Reykjavik Study**[47]	Iceland	32-60	1967	27	22,946 (50)	472 (42)	F/NF	39	30	15	24	Age
**Shanghai Health Study**[48]	China	40-74	1997-06	10	134,335 (54)	839 (49)	F	3	70	NR	NR	Age, education, BMI, alcohol, PA, income
**SHHEC**	Scotland	25-75	1984-87	23	17,731 (51)	558 (38)	F	40	51	21	27	Age, education, BMI
**Singapore Chinese Health Study**[49]	Singapore	45-74	1993-98	12	61,320 (55)	905 (31)	F/NF	6	36	3	21	Age, dialect, year of recruitment, education, alcohol, PA
**Swedish Smoking Habit Survey**[50,51]	Sweden	18-69	1963	33	41,544 (60)	442 (45)	F	18	27	5	23	Age, area
**Swiss National Cohort** [52]	Switzerland	14-99	1977-93	19	35,703 (53)	426 (29)	F	27	43	13	24	Age, survey, education, alcohol, PA, civil status, nationality, diet
**Wen**[53]	Taiwan	35+	1982-92	20	86,580 (39)	247 (2)	F	1	41	<1	11	Age

APCSC, Asia Pacific Cohort Studies Collaboration; ANZ, Australia and New-Zealand, ARIC, Atherosclerosis Risk in Communities study; BMI, body mass index; CPS, Cancer Prevention Study; CHD, coronary heart disease; DM, diabetes mellitus; EHS, Elderly Health Services; EPIC, European Prospective Investigation into Cancer; F, fatal; FEV, forced expiratory volume, JACC, Japan Collaborative Cohort study; JPHC, Japan Public Health Centre study; NF, non-fatal; NHANES III, National Health And Nutrition Examination Survey III; NHIS, ; NIH-AARP, NIH-AARP Diet and Health Study ; NR, not reported; PA, physical activity; SES, socioeconomic status; SHHEC, Scottish Heart Health Extended Cohort Study; SBP, systolic blood pressure; TC, total cholesterol; TG, triglycerides; TPCS, Three-Prefecture Cohort Study

For peer review only - http://bmjopen.bmj.com/site/about/guidelines.xhtml

Table 2: Maximum-adjusted women to men ratio of RR associated with smoking in subgroup analyses

	Former vs. never	P for interaction	Current vs. not	P for interaction
**Study region**				
Asia	1.07 (0.83, 1.37)		0.93 (0.72, 1.20)	
Non-Asia	0.73 (0.58, 0.93)	0.06	0.87 (0.66, 1.14)	0.73
**Study region**				
Asia	1.07 (0.83, 1.37)		0.93 (0.72, 1.20)	
USA	0.60 (0.42, 0.84)		0.58 (0.37, 0.91)	
Europe	0.81 (0.60, 1.10)		0.99 (0.63, 1.57)	
ANZ	1.41 (0.65, 3.04)	0.55	1.11 (0.97, 1.28)	0.69
**Year of study baseline**				
1985 or before	0.79 (0.56, 1.12)		0.96 (0.66, 1.40)	
After 1985	0.92 (0.73, 1.16)	0.43	0.85 (0.68, 1.06)	0.66
**Women to men smoking prevalence**				
>67% lower in women	1.16 (0.83, 1.62)		0.96 (0.72, 1.28)	
33-67% lower in women	0.72 (0.51, 1.03)		0.75 (0.50, 1.14)	
0-33% lower in women	0.78 (0.59, 1.03)	0.26	0.97 (0.59, 1.58)	0.90
**Women to men lung cancer rate**				
>= 50% lower in women	0.85 (0.65, 1.10)		0.85 (0.62, 1.17)	
0- 50% lower in women	0.83 (0.61, 1.13)	0.79	0.94 (0.68, 1.31)	0.64
**Study endpoint**				
Fatal lung cancer only	0.94 (0.68, 1.29)		0.97 (0.77, 1.21)	
Fatal and non-fatal lung cancer	0.80 (0.64, 1.01)	0.57	0.72 (0.48, 1.06)	0.21

For peer review only

1. Institute for Health Metrics and Evaluation. Global Burden of Disease 2015. 2015. http://vizhub.healthdata.org/gbd-compare/#
2. Reitsma MB, Fullman N, Ng M, et al. Smoking prevalence and attributable disease burden in 195 countries and territories, 1990–2015: a systematic analysis from the Global Burden of Disease Study 2015. *The Lancet* 2017.
3. Huxley RR, Woodward M. Cigarette smoking as a risk factor for coronary heart disease in women compared with men: a systematic review and meta-analysis of prospective cohort studies. *The Lancet* 2011; **378**(9799): 1297-305.
4. Lee PN, Forey BA, Coombs KJ. Systematic review with meta-analysis of the epidemiological evidence in the 1900s relating smoking to lung cancer. *BMC cancer* 2012; **12**(1): 385.
5. Thun MJ, Carter BD, Feskanich D, et al. 50-year trends in smoking-related mortality in the United States. *New England Journal of Medicine* 2013; **368**(4): 351-64.
6. Yun YD, Back JH, Ghang H, et al. Hazard ratio of smoking on lung cancer in Korea according to histological type and gender. *Lung* 2016; **194**(2): 281-9.
7. Powell HA, Iyen-Omofoman B, Hubbard RB, Baldwin DR, Tata LJ. The association between smoking quantity and lung cancer in men and women. *CHEST Journal* 2013; **143**(1): 123-9.
8. Pirie K, Peto R, Reeves GK, Green J, Beral V, Collaborators MWS. The 21st century hazards of smoking and benefits of stopping: a prospective study of one million women in the UK. *The Lancet* 2013; **381**(9861): 133-41.
9. Doll R, Peto R, Boreham J, Sutherland I. Mortality in relation to smoking: 50 years' observations on male British doctors. *BMJ (Clinical research ed)* 2004; **328**(7455): 1519.
10. Olsson AC, Gustavsson P, Zaridze D, et al. Lung cancer risk attributable to occupational exposures in a multicenter case-control study in Central and Eastern Europe. *Journal of occupational and environmental medicine* 2011; **53**(11): 1262-7.
11. Nelson DE, Davis RM, Chrismon JH, Giovino GA. Pipe smoking in the United States, 1965–1991: prevalence and attributable mortality. *Preventive medicine* 1996; **25**(2): 91-9.
12. Melikian AA, Djordjevic MV, Hosey J, et al. Gender differences relative to smoking behavior and emissions of toxins from mainstream cigarette smoke. *Nicotine & Tobacco Research* 2007; **9**(3): 377-87.
13. Ramroth H, Dietz A, Becher H. Intensity and inhalation of smoking in the aetiology of laryngeal cancer. *International journal of environmental research and public health* 2011; **8**(4): 976-84.
14. McCormack VA, Agudo A, Dahm CC, et al. Cigar and pipe smoking and cancer risk in the European Prospective Investigation into Cancer and Nutrition (EPIC). *International Journal of Cancer* 2010; **127**(10): 2402-11.
15. Ockene JK, Kuller LH, Svendsen KH, Meilahn E. The relationship of smoking cessation to coronary heart disease and lung cancer in the Multiple Risk Factor Intervention Trial (MRFIT). *American journal of public health* 1990; **80**(8): 954-8.
16. Zang EA, Wynder EL. Differences in lung cancer risk between men and women: examination of the evidence. *Journal of the National Cancer Institute* 1996; **88**(3-4): 183-92.
17. Thun MJ, Hannan LM, Adams-Campbell LL, et al. Lung cancer occurrence in never-smokers: an analysis of 13 cohorts and 22 cancer registry studies. *PLoS medicine* 2008; **5**(9): e185.
18. Freedman ND, Leitzmann MF, Hollenbeck AR, Schatzkin A, Abnet CC. Cigarette smoking and subsequent risk of lung cancer in men and women: analysis of a prospective cohort study. *The lancet oncology* 2008; **9**(7): 649-56.
19. Mitchell P, Thursfield VJ, Ball DL, et al. Lung cancer in Victoria: are we making progress. *The Medical journal of Australia* 2013; **199**(10): 674-9.
20. Sisti J, Boffetta P. What proportion of lung cancer in never-smokers can be attributed to known risk factors? *International journal of cancer* 2012; **131**(2): 265-75.

For peer review only

21. Hosgood III HD, Boffetta P, Greenland S, et al. In-home coal and wood use and lung cancer risk: a pooled analysis of the International Lung Cancer Consortium. *Environmental health perspectives* 2010; **118**(12): 1743.

22. Patrick DL, Cheadle A, Thompson DC, Diehr P, Koepsell T, Kinne S. The validity of self-reported smoking: a review and meta-analysis. *American journal of public health* 1994; **84**(7): 1086-93.

23. Jung-Choi K-H, Khang Y-H, Cho H-J. Hidden female smokers in Asia: a comparison of self-reported with cotinine-verified smoking prevalence rates in representative national data from an Asian population. *Tobacco Control* 2012; **21**(6): 536-42.

24. Huxley R, Jamrozik K, Lam TH, et al. Impact of smoking and smoking cessation on lung cancer mortality in the Asia-Pacific region. *American journal of epidemiology* 2007; **165**(11): 1280-6.

25. Prizment AE, Yatsuya H, Lutsey PL, et al. Smoking behavior and lung cancer in a biracial cohort: The atherosclerosis risk in communities study. *American journal of preventive medicine* 2014; **46**(6): 624-32.

26. Chen Z, Peto R, Zhou M, et al. Contrasting male and female trends in tobacco-attributed mortality in China: evidence from successive nationwide prospective cohort studies. *The Lancet* 2015; **386**(10002): 1447-56.

27. Chen ZM, Peto R, Iona A, et al. Emerging tobacco-related cancer risks in China: A nationwide, prospective study of 0.5 million adults. *Cancer* 2015; **121**(S17): 3097-106.

28. Gu D, Kelly TN, Wu X, et al. Mortality attributable to smoking in China. *New England Journal of Medicine* 2009; **360**(2): 150-9.

29. Prescott E, Osier M, Rein HO, et al. Gender and smoking-related risk of lung cancer. *Epidemiology (Cambridge, Mass)* 1998; **9**(1): 79-83.

30. Marugame T, Sobue T, Satoh H, et al. Lung cancer death rates by smoking status: Comparison of the Three-Prefecture Cohort study in Japan to the Cancer Prevention Study II in the USA. *Cancer science* 2005; **96**(2): 120-6.

31. Lam TH, Li ZB, Ho SY, et al. Smoking, quitting and mortality in an elderly cohort of 56 000 Hong Kong Chinese. *Tobacco control* 2007; **16**(3): 182-9.

32. Lam TH, Xu L, Schooling CM, Chan WM, Lee SY, Leung GM. Smoking and mortality in a prospective cohort study of elderly Chinese in Hong Kong. *Addiction* 2015; **110**(3): 502-10.

33. Agudo A, Bonet C, Travier N, et al. Impact of cigarette smoking on cancer risk in the European prospective investigation into cancer and nutrition study. *Journal of Clinical Oncology* 2012; **30**(36): 4550-7.

34. Sobue T, Yamamoto S, Hara M, Sasazuki S, Sasaki S, Tsugane S. Cigarette smoking and subsequent risk of lung cancer by histologic type in middle-aged Japanese men and women: The JPHC study. *International journal of cancer* 2002; **99**(2): 245-51.

35. Akiba S, Hirayama T. Cigarette smoking and cancer mortality risk in Japanese men and women--results from reanalysis of the six-prefecture cohort study data. *Environmental health perspectives* 1990; **87**: 19.

36. Katanoda K, Marugame T, Saika K, et al. Population attributable fraction of mortality associated with tobacco smoking in Japan: a pooled analysis of three large-scale cohort studies. *Journal of epidemiology* 2008; **18**(6): 251-64.

37. Jee SH, Samet JM, Ohrr H, Kim JH, Kim IS. Smoking and cancer risk in Korean men and women. *Cancer Causes & Control* 2004; **15**(4): 341-8.

38. Ekberg-Aronsson M, Nilsson PM, Nilsson J-Å, Löfdahl C-G, Löfdahl K. Mortality risks among heavy-smokers with special reference to women: a long-term follow-up of an urban population. *European journal of epidemiology* 2007; **22**(5): 301-9.

39. Engeland A, Haldorsen T, Andersen A, Tretli S. The impact of smoking habits on lung cancer risk: 28 years' observation of 26,000 Norwegian men and women. *Cancer Causes and Control* 1996; **7**(3): 366-76.

For peer review only

40. Blakely T, Barendregt JJ, Foster RH, et al. The association of active smoking with multiple cancers: national census-cancer registry cohorts with quantitative bias analysis. *Cancer Causes & Control* 2013; **24**(6): 1243-55.
41. Jha P, Ramasundarahettige C, Landsman V, et al. 21st-century hazards of smoking and benefits of cessation in the United States. *New England Journal of Medicine* 2013; **368**(4): 341-50.
42. Freedman ND, Abnet CC, Caporaso NE, et al. Impact of changing US cigarette smoking patterns on incident cancer: risks of 20 smoking-related cancers among the women and men of the NIH-AARP cohort. *International journal of epidemiology* 2016; **45**(3): 846-56.
43. Bjartveit K, Tverdal A. Health consequences of sustained smoking cessation. *Tobacco control* 2009; **18**(3): 197-205.
44. Bjartveit K, Tverdal A. Health consequences of smoking 1–4 cigarettes per day. *Tobacco control* 2005; **14**(5): 315-20.
45. Vollset SE, Tverdal A, Gjessing HkK. Smoking and deaths between 40 and 70 years of age in women and men. *Annals of internal medicine* 2006; **144**(6): 381-9.
46. Marang-van de Mheen PJ, Smith GD, Hart CL, Hole DJ. Are women more sensitive to smoking than men? Findings from the Renfrew and Paisley study. *International journal of epidemiology* 2001; **30**(4): 787-92.
47. Tulinius H, Sigfusson N, Sigvaldason H, Bjarnadottir K, Tryggvadottir L. Risk factors for malignant diseases: a cohort study on a population of 22,946 Icelanders. *Cancer epidemiology, biomarkers & prevention : a publication of the American Association for Cancer Research, cosponsored by the American Society of Preventive Oncology* 1997; **6**(11): 863-73.
48. Wang Y-Y, Zhang W, Li H-L, et al. Population attributable risks of cigarette smoking for deaths of all causes, all cancers and other chronic diseases among adults aged 40-74 years in urban Shanghai, China. *Chinese Journal of Cancer Research* 2015; **27**(1): 59.
49. Shankar A, Yuan J-M, Koh W-P, Lee H-P, Mimi CY. Morbidity and mortality in relation to smoking among women and men of Chinese ethnicity: the Singapore Chinese Health Study. *European journal of cancer* 2008; **44**(1): 100-9.
50. Nilsson S, Carstensen J, Pershagen G. Mortality among male and female smokers in Sweden: a 33 year follow up. *Journal of epidemiology and community health* 2001; **55**(11): 825-30.
51. Nordlund LA, Carstensen JM, Pershagen G. Are male and female smokers at equal risk of smoking-related cancer: evidence from a Swedish prospective study. *Scandinavian journal of public health* 1999; **27**(1): 56-62.
52. Meyer J, Rohrmann S, Bopp M, Faeh D. Impact of smoking and excess body weight on overall and site-specific cancer mortality risk. *Cancer Epidemiology and Prevention Biomarkers* 2015; **24**(10): 1516-22.
53. Wen C-P, Tsai SP, Chen C-J, Cheng T-Y. The mortality risks of smokers in Taiwan: Part I: cause-specific mortality. *Preventive medicine* 2004; **39**(3): 528-35.

For peer review only

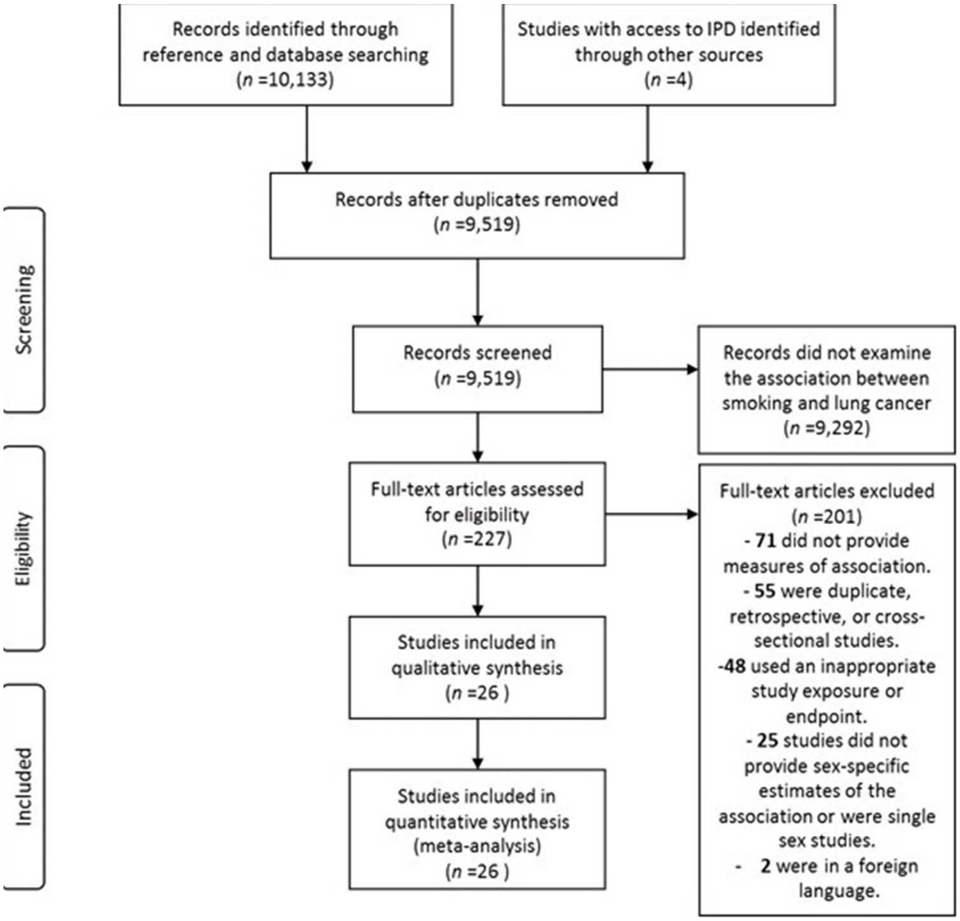

Figure 1: Flow chart of study selection
Legend: APCSC, Asia Pacific Cohort Studies Collaboration; ANZ, Australia and New-Zealand, ARIC, Atherosclerosis Risk in Communities study; BMI, body mass index; CPS, Cancer Prevention Study; CHD, coronary heart disease; DM, diabetes mellitus; EHS, Elderly Health Services; EPIC, European Prospective Investigation into Cancer; F, fatal; FEV, forced expiratory volume, JACC, Japan Collaborative Cohort study; JPHC, Japan Public Health Centre study; NF, non-fatal; NHANES III, National Health And Nutrition Examination Survey III; NHIS, ; NIH-AARP, NIH-AARP Diet and Health Study ; NR, not reported; PA, physical activity; SES, socioeconomic status; SHHEC, Scottish Heart Health Extended Cohort Study; SBP, systolic blood pressure; TC, total cholesterol; TG, triglycerides; TPCS, Three-Prefecture Cohort Study

53x55mm (300 x 300 DPI)

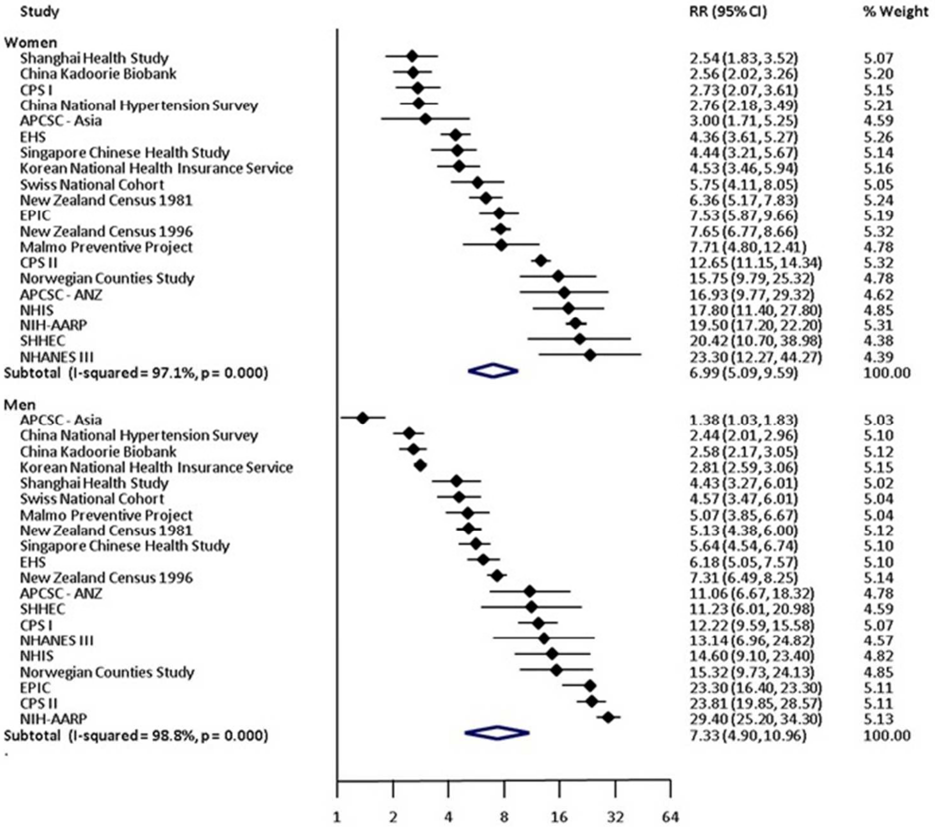

Figure 2: Multiple-adjusted relative risk for incident lung cancer in women and men, comparing current smokers to non-smokers

62x53mm (300 x 300 DPI)

only

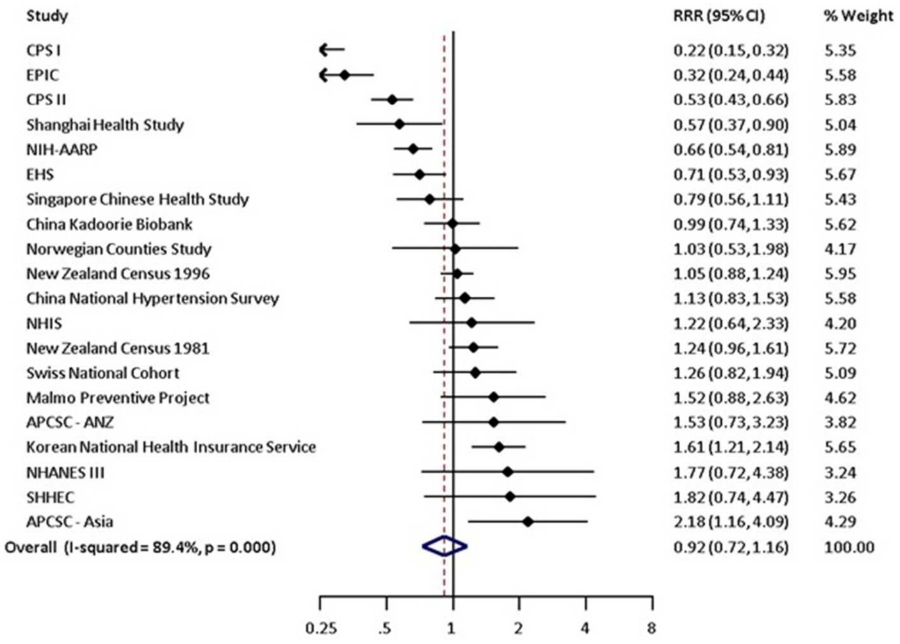

Figure 3: Multiple-adjusted women-to-men ratio of relative risks for incident lung cancer, comparing current smokers to non-smokers
Legend: APCSC, Asia Pacific Cohort Studies Collaboration; ANZ, Australia and New-Zealand, ARIC, Atherosclerosis Risk in Communities study; BMI, body mass index; CPS, Cancer Prevention Study; CHD, coronary heart disease; DM, diabetes mellitus; EHS, Elderly Health Services; EPIC, European Prospective Investigation into Cancer; F, fatal; FEV, forced expiratory volume, JACC, Japan Collaborative Cohort study; JPHC, Japan Public Health Centre study; NF, non-fatal; NHANES III, National Health And Nutrition Examination Survey III; NHIS, ; NIH-AARP, NIH-AARP Diet and Health Study ; NR, not reported; PA, physical activity; SES, socioeconomic status; SHHEC, Scottish Heart Health Extended Cohort Study; SBP, systolic blood pressure; TC, total cholesterol; TG, triglycerides; TPCS, Three-Prefecture Cohort Study

59x42mm (300 x 300 DPI)

Supplementary Material

Smoking as a risk factor for lung cancer in women and men: a systematic review and meta-analysis

eAppendix 1: MOOSE checklist

eAppendix 2: Details of search strategy

eTable 1: Quality assessment of the included studies

eTable 2: Sex-specific pooled relative risks and ratio of RRs for lung cancer associated with smoking

eFigure 1: Age-adjusted relative risk for incident lung cancer in women and men, comparing current smokers to non-smokers

eFigure 2: Age-adjusted women-to-men ratio of relative risks for incident lung cancer, comparing current smokers to non-smokers

eFigure 3: Funnel plots with pseudo 95% confidence limits for RRRs in age-adjusted in multiple-adjusted analyses on the risk of lung cancer associated with current and former smoking

eFigure 4: Age-adjusted relative risk for incident lung cancer in women and men, comparing former smokers to never smokers

eFigure 5: Age-adjusted women-to-men ratio of relative risks for incident lung cancer, comparing former smokers to never smokers

eFigure 6: Multiple-adjusted relative risk for incident lung cancer in women and men, comparing former smokers to never smokers

eFigure 7: Multiple-adjusted women-to-men ratio of relative risks for incident lung cancer, comparing former smokers to never smokers

For peer review only

For peer review only - http://bmjopen.bmj.com/site/about/guidelines.xhtml

eAppendix 1: MOOSE checklist

Item No	Recommendation	Reported on Page No
Reporting of background should include		
1	Problem definition	4
2	Hypothesis statement	4
3	Description of study outcome(s)	4
4	Type of exposure or intervention used	4
5	Type of study designs used	4
6	Study population	4
Reporting of search strategy should include		
7	Qualifications of searchers (eg, librarians and investigators)	6
8	Search strategy, including time period included in the synthesis and key words	6
9	Effort to include all available studies, including contact with authors	6
10	Databases and registries searched	6
11	Search software used, name and version, including special features used (eg, explosion)	6
12	Use of hand searching (eg, reference lists of obtained articles)	6
13	List of citations located and those excluded, including justification	13
14	Method of addressing articles published in languages other than English	6
15	Method of handling abstracts and unpublished studies	6
16	Description of any contact with authors	6
Reporting of methods should include		
17	Description of relevance or appropriateness of studies assembled for assessing the hypothesis to be tested	6&7
18	Rationale for the selection and coding of data (eg, sound clinical principles or convenience)	6&7
19	Documentation of how data were classified and coded (eg, multiple raters, blinding and interrater reliability)	6&7

For peer review only - http://bmjopen.bmj.com/site/about/guidelines.xhtml

20	Assessment of confounding (eg, comparability of cases and controls in studies where appropriate)	6&7
21	Assessment of study quality, including blinding of quality assessors, stratification or regression on possible predictors of study results	6
22	Assessment of heterogeneity	7
23	Description of statistical methods (eg, complete description of fixed or random effects models, justification of whether the chosen models account for predictors of study results, dose-response models, or cumulative meta-analysis) in sufficient detail to be replicated	7
24	Provision of appropriate tables and graphics	13-18
Reporting of results should include		
25	Graphic summarizing individual study estimates and overall estimate	14&15
26	Table giving descriptive information for each study included	12
27	Results of sensitivity testing (eg, subgroup analysis)	18
28	Indication of statistical uncertainty of findings	8&9

For peer review only

Item No	Recommendation	Reported on Page No
Reporting of discussion should include		
29	Quantitative assessment of bias (eg, publication bias)	eTable7
30	Justification for exclusion (eg, exclusion of non-English language citations)	6
31	Assessment of quality of included studies	eTable1
Reporting of conclusions should include		
32	Consideration of alternative explanations for observed results	10
33	Generalization of the conclusions (ie, appropriate for the data presented and within the domain of the literature review)	10
34	Guidelines for future research	11
35	Disclosure of funding source	3

From: Stroup DF, Berlin JA, Morton SC, et al, for the Meta-analysis Of Observational Studies in Epidemiology (MOOSE) Group. Meta-analysis of Observational Studies in Epidemiology. A Proposal for Reporting. *JAMA*. 2000;283(15):2008-2012. doi: 10.1001/jama.283.15.2008.

Transcribed from the original paper within the NEUROSURGERY® Editorial Office, Atlanta, GA, United Sates. August 2012.

For peer review only - http://bmjopen.bmj.com/site/about/guidelines.xhtml

Appendix 2: Details of search strategy

Date of search: 15/04/2016

EMBASE (5,545 hits)

(Tobacco.ti. or tobacco.ab. or *smoking/ or smoking.ti. or smoking.ab. or smoker.ti. or smoker.ab.) AND ((cancer.ti. or cancer.ab. or neoplasm.ti. or neoplasm.ab. or neoplasms.ti. or neoplasms.ab. or neoplastic.ti. or neoplastic.ab. or *cancer mortality/) AND (lung.ti or lung.ab. or pulmonary.ti. or pulmonary.ab.) or *carcinoma or *neoplasm or *lung cancer) AND (male.ti. or male.ab. or men.ab. or men.ti. or female.ti. or female.ab. or women.ab. or women.ti or sex.ti. or sex.ab. or gender.ti. or gender.ab. or *gender or *sex or *sex difference) AND (*cohort analysis/ or cohort.ti. or cohort.ab. or follow-up.ti. or follow-up.ab. or *follow up/ or prospective.ti. or prospective.ab. or retrospective.ti. or retrospective.ab. or longitudinal.ti. or longitudinal.ab. or *population research/ or *register or *health survey/ or *risk/ or *attributable risk/ or *population risk/ or risk ratio.ab. or risk.ti. or risk.ab. or *hazard ratio/ or rate ratio.ab.)

HUMANS, SINCE 01/01/1999

Pubmed/Medline (4,588 hits)

((((("tobacco"[MeSH Terms] OR "tobacco"[ti/ab] OR "smoking"[MeSH Terms] OR "smoker*"[ti/ab] OR "smoking"[ti/ab])) AND ("mortality"[ti/ab] OR death[ti/ab] OR "cancer"[ti/ab] OR "neoplasm"[ti/ab] OR "neoplasms"[ti/ab] OR "neoplastic"[ti/ab] OR "neoplasms"[MeSH Terms] OR "Smoking/mortality*"[MeSH Terms])) AND (("male"[mesh] OR "male"[all fields] OR "men"[mesh] OR "men"[all fields]) AND ("Female"[MeSH] OR "female"[all fields] OR "women"[mesh] OR "women"[all fields]) OR ("sex"[MeSH] OR "sex"[ti/ab] OR "gender"[ti/ab])) AND ("Cohort Studies"[Mesh] OR "Prospective Studies"[Mesh] OR "Longitudinal Studies"[Mesh] OR "cohort"[All Fields] OR "prospective"[All Fields] OR "longitudinal"[All Fields] OR "population-based"[All Fields] OR "registries"[MeSH] OR "relative risk"[all fields] OR "risk ratio"[all fields] OR "hazard ratio"[all fields] OR "rate ratio"[all fields] OR "Health Surveys"[MesH])))) Sort by: Relevance Filters: Publication date from 1999/01/01

For peer review only

For peer review only - http://bmjopen.bmj.com/site/about/guidelines.xhtml

eTable 1: Quality assessment of the included studies

	Selection	Comparability	Ascertainment of outcome	Overall quality score
**APCSC**	2	1	3	6
**ARIC**	4	1	3	8
**China Kadoorie Biobank**	3	1	3	7
**China National Hypertension Survey**	3	1	3	7
**Copenhagen Cohort Studies**	3	1	2	6
**CPS**	2	1	2	5
**EHS**	3	1	3	7
**EPIC**	4	1	3	8
**JPHC, JACC, TPCS**	2	1	3	6
**Korean Cancer Prevention Study**	3	1	2	6
**Korean National Health Insurance Service**	3	1	3	7
**Malmo Preventive Project**	2	1	2	5
**Migrant study**	3	1	3	7
**New Zealand Census**	3	1	3	7
**NHANES III**	3	1	3	7
**NHIS**	3	1	3	7
**NIH-AARP**	3	1	3	7
**Norwegian Counties Study**	2	1	3	6
**Renfrew/Paisley study**	2	1	3	6
**Reykjavik study**	3	1	3	7
**Shanghai Health Study**	3	1	3	7
**SHHEC**	2	1	3	6
**Singapore Chinese Health Study**	4	1	3	8
**Swedish smoking habit survey**	4	1	3	8
**Swiss National Cohort**	2	1	2	5
**Wen**	2	1	2	5

APCSC, Asia Pacific Cohort Studies Collaboration; ARIC, Atherosclerosis Risk in Communities study; CPS, Cancer Prevention Study; EHS, Elderly Health Services; EPIC, European Prospective Investigation into Cancer; JACC, Japan Collaborative Cohort study; JPHC, Japan Public Health Centre study; NHANES III, National Health And Nutrition Examination Survey III; NHIS, National Health Interview Survey; NIH-AARP, NIH-AARP Diet and Health Study ; SHHEC, Scottish Heart Health Extended Cohort Study; TPCS, Three-Prefecture Cohort Study.

Study quality assessment was based on the nine star Newcastle-Ottawa Scale (NOS) using pre-defined criteria namely: selection (population representativeness), comparability (Adjustment for confounders), and ascertainment of outcome. The NOS assigns a maximum of four points for selection, two points for comparability and three points for outcome. Nine points on the NOS reflects the highest study quality.

For peer review only

For peer review only - http://bmjopen.bmj.com/site/about/guidelines.xhtml

eTable 2: Sex-specific pooled relative risks and ratio of RRs for lung cancer associated with smoking

		RR in women	RR in men	Ratio of RR
Age-adjusted	Former vs. never	2.82 (2.25, 3.54)	3.01 (2.23, 4.08)	0.88 (0.69, 1.14)
	Current vs. not	7.48 (5.29. 10.60)	8.78 (6.13, 12.57)	0.81 (0.62, 1.04)
Multiple-adjusted	Former vs. never	3.14 (2.45, 4.03)	3.13 (2.06, 4.76)	0.89 (0.69, 1.13)
	Current vs. not	6.99 (5.09, 9.59)	7.33 (4.90, 10.96)	0.92 (0.72, 1.16)
Maximum-adjusted	Former vs. never	2.92 (2.35, 3.63)	3.08 (2.31, 4.11)	0.86 (0.71, 1.05)
	Current vs. not	7.32 (5.58, 9.61)	8.05 (5.90, 10.98)	0.89 (0.73, 1.08)
**Cigarettes per day among current smokers vs. never (maximum-adjusted)**				
	10 or less	5.30 (3.52, 7.97)	4.97 (2.74, 9.03)	0.99 (0.65, 1.52)
	10 to 19	10.67 (7.43, 15.33)	8.93 (4.90, 16.28)	1.11 (0.75, 1.64)
	20 or more	17.09 (12.11, 24.11)	14.61 (8.33, 25.59)	0.94 (0.69, 1.30)

For peer review only

For peer review only - http://bmjopen.bmj.com/site/about/guidelines.xhtml

eFigure 1: Age-adjusted relative risk for incident lung cancer in women and men, comparing current smokers to non-smokers

**Study**	**RR (95% CI)**	**% Weight**
**Women**		
Korean Cancer Prevention Study	2.50 (2.00, 3.10)	5.53
APCSC - Asia	2.57 (1.85, 3.56)	5.39
Shanghai Health Study	2.69 (1.96, 3.68)	5.41
CPS I	2.74 (2.07, 3.62)	5.46
Wen	3.36 (1.44, 7.86)	4.23
Korean National Health Insurance Service	3.81 (3.06, 4.74)	5.53
JPHC, JACC, TPCS	3.88 (3.07, 4.90)	5.52
EHS	4.31 (3.58, 5.19)	5.57
Swedish 1963 Smoking Habit Survey	4.87 (3.58, 6.62)	5.42
Renfrew/Paisley Study	8.94 (6.87, 11.64)	5.48
Copenhagen Cohort Studies	12.24 (8.95, 16.75)	5.41
CPS II	12.62 (11.13, 14.31)	5.62
ARIC	13.13 (8.46, 20.41)	5.19
Norwegian Counties Study	16.07 (10.16, 25.43)	5.15
APCSC - ANZ	17.05 (10.86, 26.77)	5.17
Migrant Study	17.28 (10.64, 28.06)	5.10
SHHEC	22.11 (11.68, 41.86)	4.75
Reykjavik Study	22.25 (15.29, 32.39)	5.31
NHANES III	27.02 (14.41, 50.65)	4.78
**Subtotal (I-squared = 96.4%, p = 0.000)**	7.48 (5.29, 10.60)	100.00
.		
**Men**		
APCSC - Asia	2.50 (2.11, 2.96)	5.54
Wen	2.73 (2.08, 3.59)	5.43
Korean National Health Insurance Service	3.05 (2.83, 3.29)	5.59
Korean Cancer Prevention Study	4.60 (4.00, 5.30)	5.56
JPHC, JACC, TPCS	4.79 (3.88, 5.92)	5.50
Shanghai Health Study	5.50 (4.09, 7.40)	5.40
Migrant Study	5.76 (3.85, 8.64)	5.23
EHS	6.10 (5.00, 7.43)	5.51
Swedish 1963 Smoking Habit Survey	9.40 (6.53, 13.60)	5.30
CPS I	12.49 (9.80, 15.92)	5.47
SHHEC	12.91 (6.87, 24.26)	4.78
APCSC - ANZ	13.82 (8.94, 21.38)	5.18
Reykjavik Study	13.98 (9.54, 20.49)	5.27
Norwegian Counties Study	14.33 (8.60, 23.86)	5.04
Renfrew/Paisley Study	14.95 (10.46, 21.37)	5.31
Copenhagen Cohort Studies	16.03 (10.78, 23.84)	5.25
NHANES III	16.07 (8.58, 30.11)	4.79
CPS II	25.30 (21.10, 30.34)	5.53
ARIC	41.41 (18.36, 93.42)	4.35
**Subtotal (I-squared = 97.8%, p = 0.000)**	8.78 (6.13, 12.57)	100.00
.		

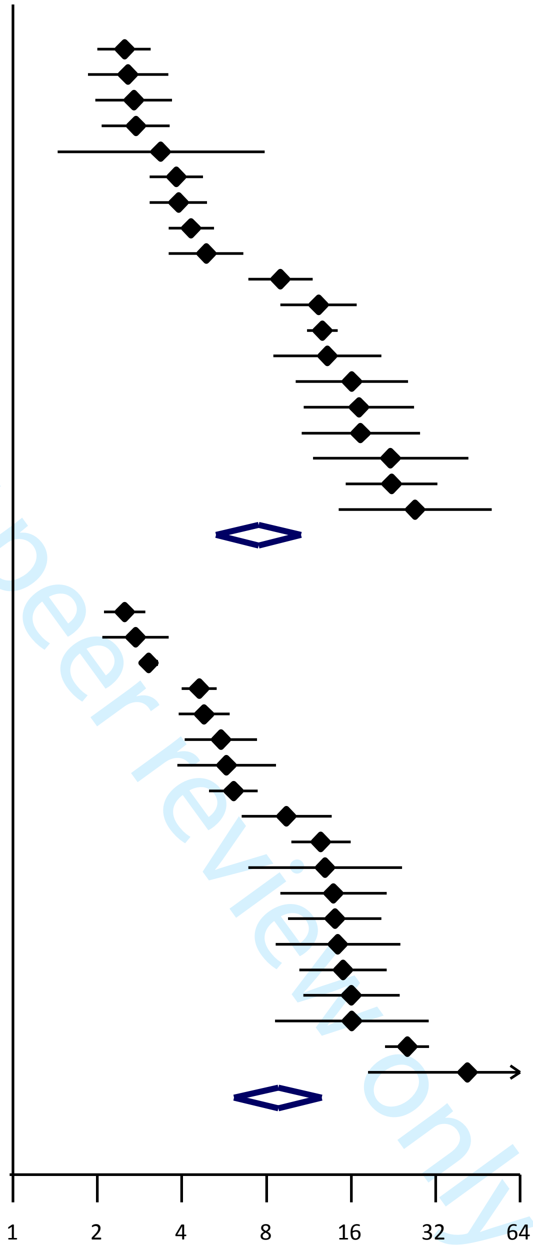

For peer review only

eFigure 2: Age-adjusted women-to-men ratio of relative risks for incident lung cancer, comparing current smokers to non-smokers

Study	RRR (95% CI)	% Weight
CPS I	0.22 (0.15, 0.32)	5.94
ARIC	0.32 (0.13, 0.80)	3.61
Shanghai Health Study	0.49 (0.32, 0.75)	5.69
CPS II	0.50 (0.40, 0.62)	6.45
Swedish 1963 Smoking Habit Survey	0.52 (0.32, 0.84)	5.49
Korean Cancer Prevention Study	0.54 (0.42, 0.71)	6.33
Renfrew/Paisley Study	0.60 (0.38, 0.93)	5.64
EHS	0.71 (0.54, 0.93)	6.30
Copenhagen Cohort Studies	0.76 (0.46, 1.27)	5.37
JPHC, JACC, TPCS	0.81 (0.59, 1.11)	6.15
APCSC - Asia	1.03 (0.71, 1.49)	5.95
Norwegian Counties Study	1.12 (0.56, 2.23)	4.57
Wen	1.23 (0.50, 3.00)	3.74
APCSC - ANZ	1.23 (0.66, 2.31)	4.83
Korean National Health Insurance Service	1.25 (0.99, 1.58)	6.42
Reykjavik Study	1.59 (0.93, 2.72)	5.24
NHANES III	1.68 (0.69, 4.09)	3.75
SHHEC	1.71 (0.70, 4.20)	3.72
Migrant Study	3.00 (1.60, 5.64)	4.81
**Overall (I-squared = 86.2%, p = 0.000)**	0.81 (0.62, 1.04)	100.00

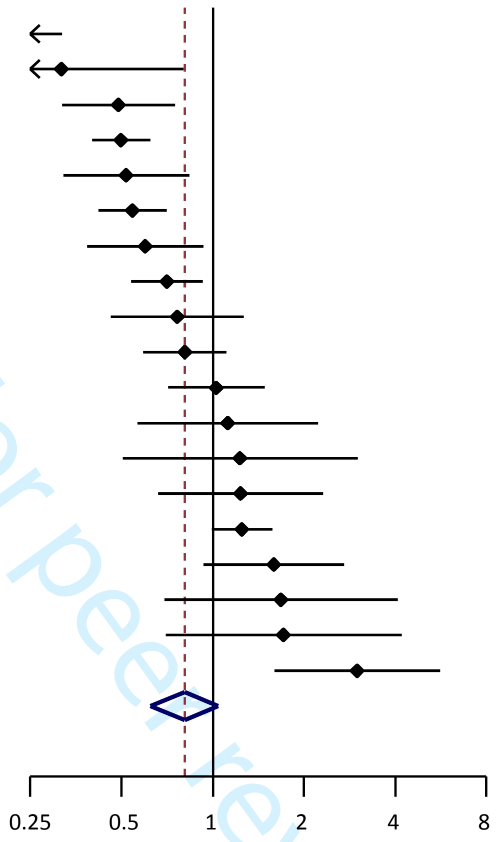

For peer review only

For peer review only - http://bmjopen.bmj.com/site/about/guidelines.xhtml

eFigure 3: Funnel plots with pseudo 95% confidence limits for RRRs in in multiple-adjusted analysis on the risk of lung cancer associated with current smoking

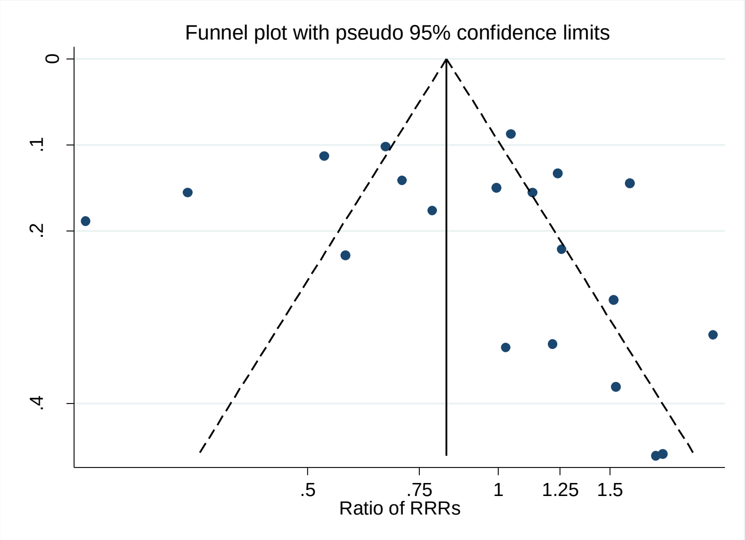

For peer review only - http://bmjopen.bmj.com/site/about/guidelines.xhtml

eFigure 4: Age-adjusted relative risk for incident lung cancer in women and men, comparing former smokers to never smokers

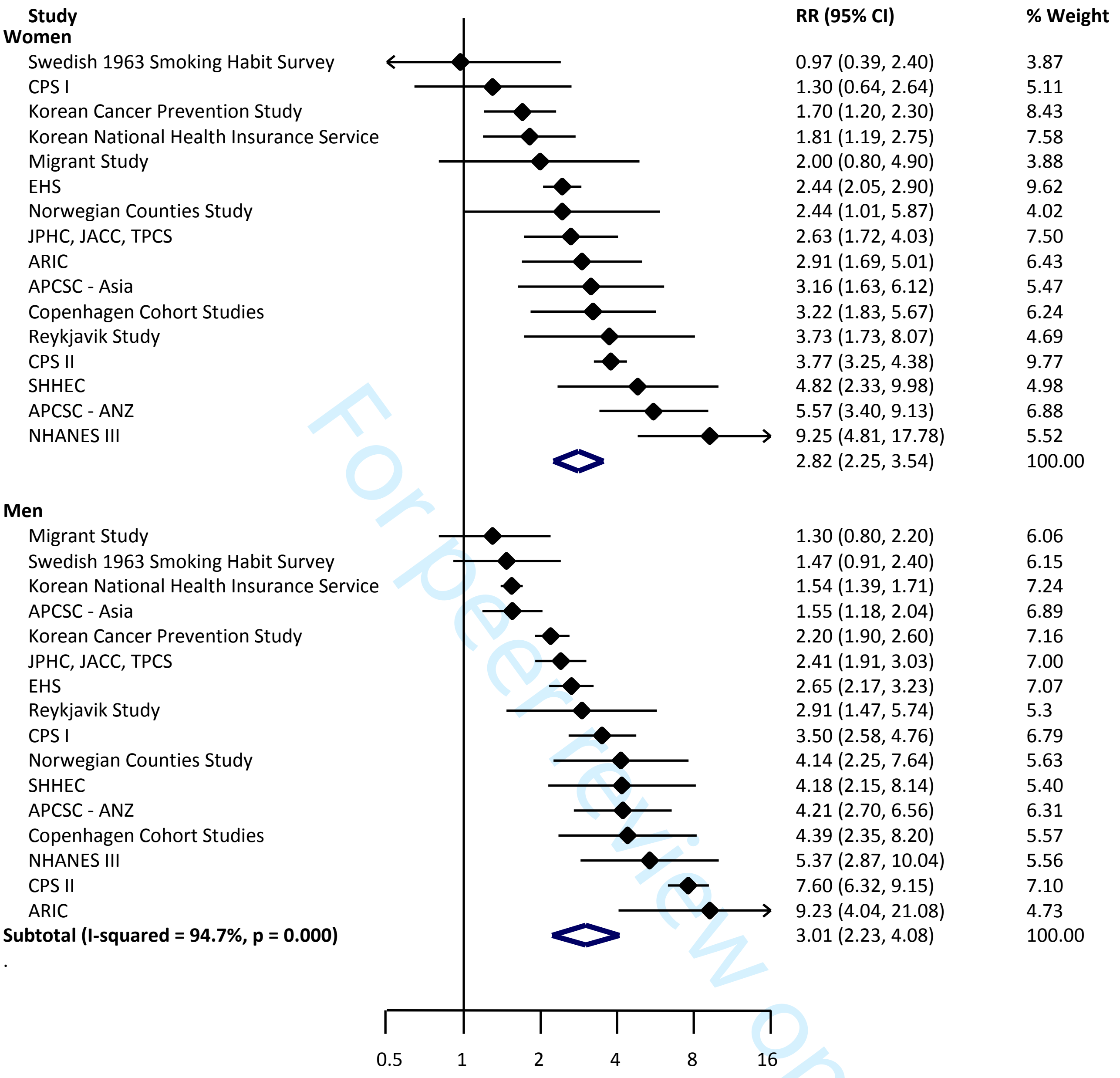

For peer review only

For peer review only - http://bmjopen.bmj.com/site/about/guidelines.xhtml

eFigure 5: Age-adjusted women-to-men ratio of relative risks for incident lung cancer, comparing former smokers to never smokers

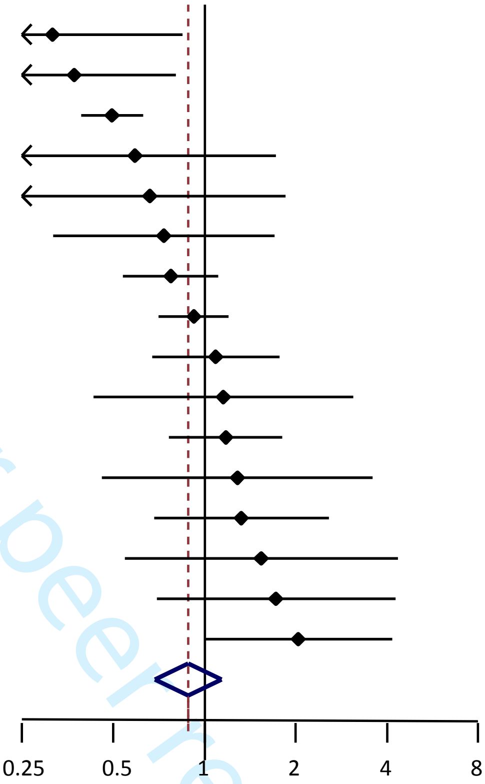

Study	RRR (95% CI)	% Weight
ARIC	0.32 (0.12, 0.85)	4.19
CPS I	0.37 (0.17, 0.80)	5.61
CPS II	0.50 (0.39, 0.63)	10.77
Norwegian Counties Study	0.59 (0.20, 1.72)	3.76
Swedish 1963 Smoking Habit Survey	0.66 (0.24, 1.85)	3.97
Copenhagen Cohort Studies	0.73 (0.32, 1.70)	5.09
Korean Cancer Prevention Study	0.77 (0.54, 1.11)	9.57
EHS	0.92 (0.71, 1.20)	10.54
JPHC, JACC, TPCS	1.09 (0.67, 1.77)	8.26
SHHEC	1.15 (0.43, 3.09)	4.20
Korean National Health Insurance Service	1.17 (0.76, 1.80)	8.84
Reykjavik Study	1.28 (0.46, 3.58)	3.98
APCSC - ANZ	1.32 (0.68, 2.57)	6.51
Migrant Study	1.54 (0.54, 4.34)	3.93
NHANES III	1.72 (0.70, 4.26)	4.68
APCSC - Asia	2.04 (1.00, 4.16)	6.07
**Overall (I-squared = 65.4%, p = 0.000)**	0.88 (0.69, 1.14)	100.00

For peer review only

eFigure 6: Multiple-adjusted relative risk for incident lung cancer in women and men, comparing former smokers to never smokers

Study	RR (95% CI)	% Weight
**Women**		
Swiss National Cohort	1.05 (0.47, 2.34)	5.13
CPS I	1.30 (0.64, 2.64)	5.81
Korean National Health Insurance Service	1.74 (1.03, 2.96)	7.32
EHS	2.48 (2.07, 2.96)	10.36
Norwegian Counties Study	2.51 (1.04, 6.06)	4.62
Singapore Chinese Health Study	2.70 (1.70, 4.30)	7.94
Malmo Preventive Project	2.71 (1.02, 7.23)	4.07
EPIC	3.28 (2.46, 4.37)	9.56
APCSC - Asia	3.49 (1.63, 7.47)	5.41
CPS II	3.85 (3.32, 4.47)	10.53
SHHEC	4.84 (2.34, 10.02)	5.67
APCSC - ANZ	5.22 (2.91, 9.36)	6.83
NIH-AARP	5.40 (4.80, 6.20)	10.64
NHANES III	7.66 (3.92, 14.98)	6.11
**Subtotal (I-squared = 85.2%, p = 0.000)**	3.14 (2.45, 4.03)	100.00
**Men**		
Malmo Preventive Project	1.21 (0.72, 2.02)	6.87
APCSC - Asia	1.36 (0.95, 1.95)	7.26
Korean National Health Insurance Service	1.45 (1.30, 1.63)	7.62
Singapore Chinese Health Study	2.00 (1.50, 2.70)	7.39
Swiss National Cohort	2.22 (1.46, 3.38)	7.12
EHS	2.73 (2.23, 3.35)	7.53
CPS I	3.48 (2.56, 4.74)	7.36
APCSC - ANZ	3.71 (2.24, 6.14)	6.90
Norwegian Counties Study	4.13 (2.24, 7.64)	6.58
SHHEC	4.21 (2.16, 8.18)	6.42
NHANES III	4.60 (2.44, 8.66)	6.52
EPIC	5.28 (3.77, 7.38)	7.30
CPS II	7.41 (6.16, 8.91)	7.55
NIH-AARP	7.60 (6.60, 8.90)	7.59
**Subtotal (I-squared = 97.2%, p = 0.000)**	3.13 (2.06, 4.76)	100.00

.

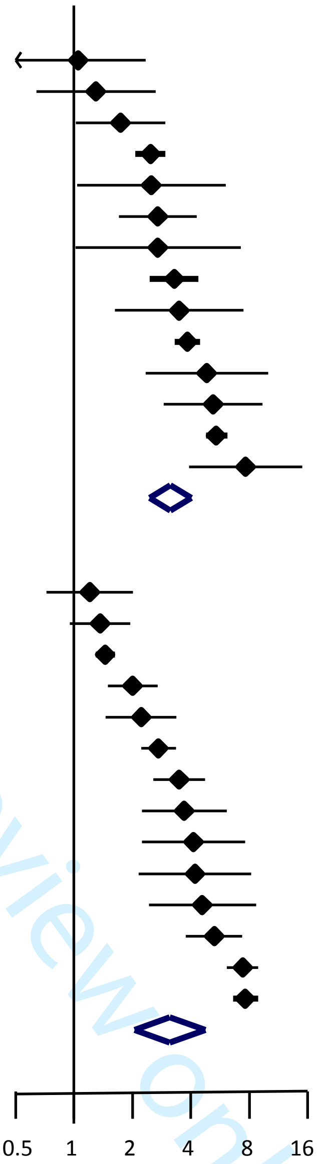

For peer review only

For peer review only - http://bmjopen.bmj.com/site/about/guidelines.xhtml

eFigure 7: Multiple-adjusted women-to-men ratio of relative risks for incident lung cancer, comparing former smokers to never smokers

Study	RRR (95% CI)	% Weight
CPS I	0.37 (0.17, 0.81)	5.83
Swiss National Cohort	0.47 (0.19, 1.17)	4.81
CPS II	0.52 (0.41, 0.66)	12.01
Norwegian Counties Study	0.61 (0.21, 1.78)	3.81
EPIC	0.62 (0.40, 0.97)	9.44
NIH-AARP	0.71 (0.58, 0.87)	12.44
EHS	0.91 (0.69, 1.19)	11.62
SHHEC	1.15 (0.43, 3.08)	4.29
Korean National Health Insurance Service	1.20 (0.70, 2.06)	8.20
Singapore Chinese Health Study	1.35 (0.78, 2.34)	8.11
APCSC - ANZ	1.41 (0.65, 3.04)	5.84
NHANES III	1.67 (0.66, 4.19)	4.70
Malmo Preventive Project	2.24 (0.74, 6.77)	3.65
APCSC - Asia	2.56 (1.10, 5.94)	5.26
**Overall (I-squared = 68.9%, p = 0.000)**	0.89 (0.69, 1.13)	100.00

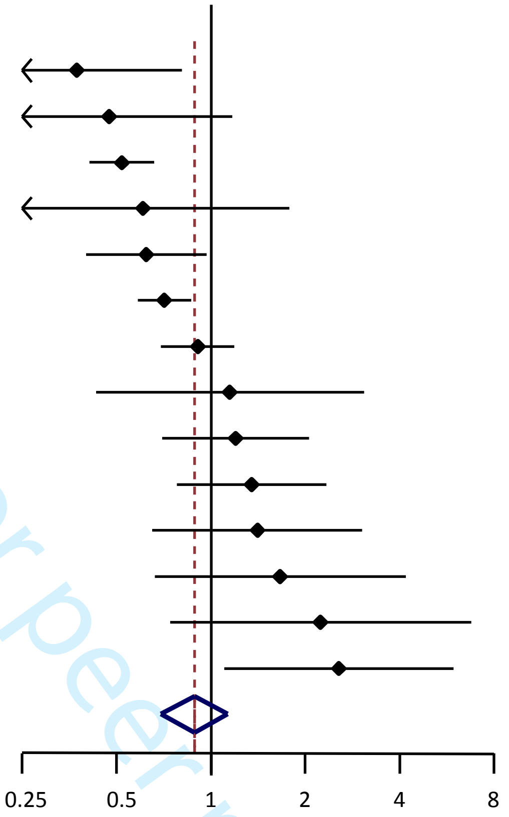

For peer review only

For peer review only

For peer review only - http://bmjopen.bmj.com/site/about/guidelines.xhtml

eAppendix 1: MOOSE checklist

Item No	Recommendation	Reported on Page No
Reporting of background should include		
1	Problem definition	4
2	Hypothesis statement	4
3	Description of study outcome(s)	4
4	Type of exposure or intervention used	4
5	Type of study designs used	4
6	Study population	4
Reporting of search strategy should include		
7	Qualifications of searchers (eg, librarians and investigators)	6
8	Search strategy, including time period included in the synthesis and key words	6
9	Effort to include all available studies, including contact with authors	6
10	Databases and registries searched	6
11	Search software used, name and version, including special features used (eg, explosion)	6
12	Use of hand searching (eg, reference lists of obtained articles)	6
13	List of citations located and those excluded, including justification	13
14	Method of addressing articles published in languages other than English	6
15	Method of handling abstracts and unpublished studies	6
16	Description of any contact with authors	6
Reporting of methods should include		
17	Description of relevance or appropriateness of studies assembled for assessing the hypothesis to be tested	6&7
18	Rationale for the selection and coding of data (eg, sound clinical principles or convenience)	6&7
19	Documentation of how data were classified and coded (eg, multiple raters, blinding and interrater reliability)	6&7
20	Assessment of confounding (eg, comparability of cases and controls in studies	6&7

For peer review only - http://bmjopen.bmj.com/site/about/guidelines.xhtml

	where appropriate)	
21	Assessment of study quality, including blinding of quality assessors, stratification or regression on possible predictors of study results	6
22	Assessment of heterogeneity	7
23	Description of statistical methods (eg, complete description of fixed or random effects models, justification of whether the chosen models account for predictors of study results, dose-response models, or cumulative meta-analysis) in sufficient detail to be replicated	7
24	Provision of appropriate tables and graphics	13-18
Reporting of results should include		
25	Graphic summarizing individual study estimates and overall estimate	14&15
26	Table giving descriptive information for each study included	12
27	Results of sensitivity testing (eg, subgroup analysis)	18
28	Indication of statistical uncertainty of findings	8&9

For peer review only

For peer review only - http://bmjopen.bmj.com/site/about/guidelines.xhtml

Item No	Recommendation	Reported on Page No
Reporting of discussion should include		
29	Quantitative assessment of bias (eg, publication bias)	eTable7
30	Justification for exclusion (eg, exclusion of non-English language citations)	6
31	Assessment of quality of included studies	eTable1
Reporting of conclusions should include		
32	Consideration of alternative explanations for observed results	10
33	Generalization of the conclusions (ie, appropriate for the data presented and within the domain of the literature review)	10
34	Guidelines for future research	11
35	Disclosure of funding source	3

From: Stroup DF, Berlin JA, Morton SC, et al, for the Meta-analysis Of Observational Studies in Epidemiology (MOOSE) Group. Meta-analysis of Observational Studies in Epidemiology. A Proposal for Reporting. *JAMA*. 2000;283(15):2008-2012. doi: 10.1001/jama.283.15.2008.

Transcribed from the original paper within the NEUROSURGERY® Editorial Office, Atlanta, GA, United Sates. August 2012.

For peer review only

For peer review only - http://bmjopen.bmj.com/site/about/guidelines.xhtml

BMJ Open

Smoking as a risk factor for lung cancer in women and men: a systematic review and meta-analysis

Journal:	*BMJ Open*
Manuscript ID	bmjopen-2018-021611.R1
Article Type:	Research
Date Submitted by the Author:	16-May-2018
Complete List of Authors:	O'Keeffe, Linda; MRC Integrative Epidemiology Unit, Bristol Medical School; Population Health Sciences, Bristol Medical School, University of Bristol<br>Taylor, Gemma; MRC Integrative Epidemiology Unit at the University of Bristol, Bristol Medical School, University of Bristol, Bristol, UK; UK Centre for Tobacco and Alcohol Studies, School of Experimental Psychology, University of Bristol, UK<br>Huxley, Rachel; School of Public Health, Faculty of Health Sciences, Curtin University,<br>Mitchell, Paul; Olivia Newton-John Cancer and Wellness Centre, Austin Health and Olivia Newton-John Cancer Research Institute, Heidelberg, VIC 3084, Australia<br>Woodward, Mark; The George Institute for Global Health,<br>Peters, Sanne; University of Oxford, George Institute for Global Health
**Primary Subject Heading**:	Epidemiology
Secondary Subject Heading:	Oncology, Public health
Keywords:	smoking, systematic review, lung cancer, sex-specific

SCHOLARONE™
Manuscripts

Smoking as a risk factor for lung cancer in women and men: a systematic review and meta-analysis

Linda M. O'Keeffe[1, 2*], Gemma Taylor[1, 2, 3*], Rachel R. Huxley[4], Paul Mitchell[5], Mark Woodward[6,7,8], Sanne A.E. Peters[6]

* Denotes equal contribution

1 MRC Integrative Epidemiology Unit at the University of Bristol, Bristol Medical School, University of Bristol, Bristol, UK
2 Population Health Sciences, Bristol Medical School, University of Bristol
3 UK Centre for Tobacco and Alcohol Studies, School of Experimental Psychology, University of Bristol, UK
4 School of Public Health, Faculty of Health Sciences, Curtin University, Perth, Australia
5 Olivia Newton-John Cancer and Wellness Centre, Austin Health and Olivia Newton-John Cancer Research Institute, Heidelberg, VIC 3084, Australia
6 The George Institute for Global Health, University of Oxford, Oxford, UK
7 The George Institute for Global Health, University of New South Wales, Sydney, Australia
8 Department of Epidemiology, Johns Hopkins University, Baltimore MD, USA

Correspondence: Dr. Linda O'Keeffe
MRC Integrative Epidemiology Unit at the University of Bristol, University of Bristol, Oakfield House, Oakfield Grove, BS8 2BN, United Kingdom T: +44 7534498919

Email address: lindaokeeffe1987@gmail.com

Funding: The MRC Integrative Epidemiology Unit at the University of Bristol is supported by the Medical Research Council and the University of Bristol [MC_UU_12013/6, MC_UU_12013/9]. LMOK is supported by a UK Medical Research Council Population Health Scientist fellowship (MR/M014509/1). GMJT is funded by a Cancer Research UK Postdoctoral Fellowship (C56067/A21330). SAEP is supported by a UK Medical Research Council Skills Development Fellowship (MR/P014550/1)

Data sharing statement: Technical appendix, statistical code, and dataset (of published data only) available from authors upon request.

Contributor statement: LMOK, GT, SAEP, RH and MW designed the study. LMOK and GT performed systematic searches, retrieved literature and performed data extraction. SAEP performed the analysis of the data. LMOK and GT wrote the first draft of the article. RH, MW and PM contributed important intellectual content and critical expertise and revisions to the manuscript.

Competing interest's statement: We have no competing interests to declare.

For peer review only

Abstract

Objectives: To investigate the sex-specific association between smoking and lung cancer.

Design: Systematic review and meta-analysis.

Data sources: We searched Pubmed and Embase, from January 1st, 1999 to April 15th, 2016 for cohort studies. Cohort studies before January 1st, 1999 were retrieved from a previous meta-analysis. Individual participant data from three sources was also available to supplement analyses of published literature.

Eligibility criteria for selecting studies: Cohort studies reporting the sex-specific relative risk (RR) of lung cancer associated with smoking.

Results: Data from 29 studies representing 99 cohort studies, seven million individuals and > 50,000 incident lung cancer cases were included. The sex-specific RRs and their ratio comparing women with men were pooled using random-effects meta-analysis with inverse-variance weighting. The pooled multiple-adjusted lung cancer RR was 6.99 (95% Confidence Interval (CI): 5.09, 9.59) in women and 7.33 (95% CI: 4.90, 10.96) in men. The pooled ratio of the RRs was 0.92 (95% CI: 0.72 – 1.16; I^2=89%; p<0.001), with no evidence of publication bias, or differences across major predefined participant and study subtypes. The women-to-men ratio of RRs were 0.99 (95% CI: 0.65, 1.52), 1.11 (95% CI: 0.75, 1.64), and 0.94 (95% CI: 0.69, 1.30), for light, moderate and heavy smoking respectively.

Conclusions: Smoking yields similar risks of lung cancer in women compared with men. However, these data may underestimate the true risks of lung cancer among women, as the smoking epidemic has not yet reached full maturity in women. Continued efforts to measure the sex-specific association of smoking with lung cancer are required.

For peer review only - http://bmjopen.bmj.com/site/about/guidelines.xhtml

Strengths and Limitations

- Evidence on the sex-specific association of smoking and lung cancer were meta-analysed in over seven million participants across 99 cohort studies.
- Several subgroup analyses were performed to examine the robustness of findings across different population subgroups.
- However, the smoking epidemic is not yet fully mature in women and risks of lung cancer in women may still be underestimated.
- Detailed data on smoking behaviour and data on specific subtypes of lung cancer were not available.

For peer review only

For peer review only - http://bmjopen.bmj.com/site/about/guidelines.xhtml

Introduction

Lung cancer is the leading cause of cancer death worldwide with 1.7 million global deaths attributed to cigarette smoking in 2015.[1] Tobacco use is the leading cause of lung cancer; 55% of lung cancer deaths in women and over 70% of lung cancer deaths in men are due to smoking.[1] These global estimates, however, mask major differences in smoking prevalence in men and women across populations, with rates below 5% for women in most Asian and African countries to 40% and above for men in many parts of Asia and Eastern Europe.[2] [2] In addition, smoking behaviour varies significantly by sex. For example, compared with women, men smoke more cigars and pipes,[3] take puffs of longer duration, and leave shorter butts,[4] which each could potentially predispose them to greater risks of smoking-related lung cancer. Substantial physiological differences between the sexes may also result in sex differences in the effects of smoking, particularly for women. For example, compared with men, women have a smaller lung size and different airway behaviour,[5] which may increase their susceptibility to lung cancer at lower levels of smoking. A recent meta-analysis showed that cigarette smoking confers a greater coronary hazard in women compared with men, which suggests the possibility that this may also be true for the risk of smoking-related lung cancer.[6]

A study of 50-year trends in smoking-related mortality in the US found that the relative risks of smoking-related lung cancer mortality were higher in men than women.[7] However, this sex difference was only apparent in the oldest cohorts with the longest follow-up, possibly reflecting greater cumulative tobacco exposure in men than in women. In contrast, a recent study in Korea, a population where smoking patterns continue to differ between the sexes, suggested that sex differences in the impact of smoking on lung cancer risk exist and differ by histologic subtype.[8] Analyses of a large UK primary care database showed that moderate and heavy smoking more strongly increase the risks of lung cancer in women than in men. [9]

Two recent meta-analyses examined the sex-specific association between smoking and lung cancer. In the most recent of these, males were found to have a greater risk of lung cancer associated with smoking compared with females.[10] However, virtually all data were from historical

For peer review only - http://bmjopen.bmj.com/site/about/guidelines.xhtml

case-control studies, which have several limitations, and the three included prospective studies provided contradictory results. While a previous meta-analysis by Lee et al [11] included 287 cohort and case-control studies and provided sex-specific estimates, single-sex cohorts were also included, sex differences in the smoking-related risk of lung cancer were not formally compared within studies, and only studies published up-to 1999 were included.

To resolve this uncertainty, we performed a systematic review and meta-analysis of prospective cohort studies published to date on the sex-specific association of smoking on the risk of fatal and non-fatal lung cancer. Our systematic review builds on these previous meta-analyses by adding literature from 1999 onwards and restricting the analyses to cohort studies, which are less prone to bias than case-control studies. In addition, we perform several pre-defined subgroup analyses which have not been performed in meta-analyses of cohort studies included in previous reviews and supplement our findings with results from three sources of individual participant data, not published previously. An important *a priori* consideration is the substantial sex difference in the maturity of the smoking epidemic with men being at a more advanced stage than women in most parts of the world.[2] This would be expected to translate into lower relative risk estimates for lung cancer in women than in men. Hence, the null hypothesis that smoking confers the same lung cancer hazard in both women and men, would be met if the ratio of the relative risks for lung cancer (women:men) was less than unity (reflecting a greater hazard in men than women). However, if the ratio of the relative risks was found to be unity (or higher) then this would suggest a greater hazard associated with tobacco exposure in women than in men.

For peer review only - http://bmjopen.bmj.com/site/about/guidelines.xhtml

Methods

Search strategy

This review was conducted using a predefined protocol and in accordance to the MOOSE guidelines (**eAppendix 1**). We systematically searched PubMed and EMBASE for studies published between January 1, 1999 and April 15th, 2016 that reported on the relationship between smoking and lung cancer in men and women from a general population. The computer-based searches combined MeSH and free-text terms related to “tobacco/smoking”, “cancer”, “sex” and “cohort studies”. The full search criteria are available in **eAppendix 2**. Articles published before January 1st , 1999 were retrieved from a previous systematic review.[11] The reference lists of all relevant original research and review articles were scanned to capture missed studies. Two authors (LMOK and GMT) independently conducted the screening of studies and any disagreement was mediated by a third author (SAP).

Data extraction

Data were extracted, in duplicate, from studies deemed to meet the eligibility criteria. These included details on general study characteristics (study name, duration of follow-up, year of publication), information about the studied population (prevalence of smoking, mean age, number of men and women, incidence of lung cancer, whether lung cancer was fatal or non-fatal, and level of adjustment for covariates). We extracted sex-specific adjusted measures of relative risk and 95% confidence intervals.

Study selection

Observational cohort studies were included if they reported sex-specific relative risks (RRs), or equivalent, on the relationship between smoking and lung cancer. Studies were excluded if the variability around the point estimate was not reported, if they had not been adjusted for at least age, or if the study was performed in a population selected on the basis of prior lung cancer or

For peer review only - http://bmjopen.bmj.com/site/about/guidelines.xhtml

another major underlying chronic disease. In the case of duplicate reports from the same study, the report with the longest follow-up or the highest number of cases was included. Individual participant data (IPD) from studies available to the authors were also used; the Asia Pacific Cohort Studies Collaboration (APCSC), the National Health and Nutrition Examination Survey III (NHANES III), and the Scottish Heart Health Extended Cohort Study (SHHEC). The Newcastle-Ottawa Quality assessment (NOS) scale was used to assess the methodological quality of all included studies, on a 9-point scale (**eAppendix 3 & eTable 1**).[12]

Meta-analysis

The primary analysis was a comparison of the sex-specific RR of lung cancer (fatal or non-fatal) in current smokers versus non-smokers (defined either as former or never smokers). For each study, we obtained the natural log of the sex-specific RRs and calculated the differences. The differences were pooled across studies using random effects meta-analysis which allows the RR of lung cancer to vary from study to study, weighted by the inverse of the variances of the log RRs, and then back-transformed to obtain the pooled women-to-men ratio of the RRs (RRR). The standard error of the log RRR was calculated as the square root of the sum hetof the variance of the two sex-specific log RRs for each study. Pooled RRRs were computed separately for studies with only age-adjusted estimates and then for those studies with multiple-adjusted estimates. The set of multiple adjustments made was allowed to vary by study, but had to include at least one other risk factor in addition to age. The I^2 statistic was used to estimate the percentage of variability across studies due to between-study heterogeneity. The presence of publication bias was graphically examined using contour funnel plots, plotting the natural log of the RRR against its standard error, and tested using Begg's test. Pre-defined subgroup analyses were conducted to obtain the adjusted RRRs by study region (Asia or non-Asia and Asia, Europe, US, and Australia and New Zealand [ANZ]), year of study baseline (pre- or post-1985), study endpoint (fatal only or fatal and non-fatal combined), number of cigarettes smoked per day (>0 to 10, 10-20, >20), study quality ≤6 vs. >6 points) and follow-up time

For peer review only - http://bmjopen.bmj.com/site/about/guidelines.xhtml

(≤10 vs. >10 years). Random effects meta-analysis were used for all subgroup analyses and differences between subgroups were examined using meta-regression. To include the largest number of studies available, we combined the age-and multiple-adjusted estimates, taking the maximum available adjustment set available. In secondary analyses, we obtained the sex-specific RRs and RRRs comparing former smokers to never smokers and performed the same set of subgroup analyses. All analyses were performed using Stata version 12.0.

Patient and public involvement

There were no patients or applicable public involved in this review.

For peer review only

Results

Of the 9,519 unique records that were identified through the systematic search, 227 qualified for full-text evaluation (**Figure 1**). Of these, 25 separate studies provided information about sex differences in the association between smoking and lung cancer. This database was extended with individual participant data from APCSC (separately for Asia and ANZ), NHANES III, and SHHEC leading to a total of 29 individual estimates, representing a total of 99 cohort studies available for meta-analysis.

The characteristics of the included studies are described in **Table 1**. Overall, data were available from 99 cohorts, including 7,113,303 individuals (46% women) - not accounting for two cohorts that used Census data - and at least 51,161 incident cases of lung cancer (31% women). Forty-six cohorts were from Asia (61% of the individuals), six were from the USA (28%), 27 were from Europe (10%), and ten were from Australia and New Zealand (1%). Of 29 studies, four studies had a quality score of 5 out of 9, 9 studies had a score of 6, 12 studies had a score of 7 and 4 studies with a score of 8 (**eTable 1**).

Eighteen studies reported on the prevalence of smoking, which varied widely by study, region, and sex. The prevalence of smoking ranged from 1% to 47% in women and from 1% to 70% in men. In all but two studies, the prevalence of smoking was higher in men than women, especially in Asia where typically less than 10% of women were smokers compared with over 50% of men. Smoking cessation rates were also higher among men (7-61%) than women (<1-39%).

Risk of lung cancer in current smokers versus non-smokers

Compared with non-smoking, current smoking was associated with an age-adjusted RR of lung cancer of 7.48 (95% Confidence Interval (CI): 5.29, 10.60) in women and 8.78 (95% CI: 6.13, 12.57) in men (**Table 2 & eFigure 1**). The pooled age-adjusted women-to-men RRR was 0.81 (95% CI: 0.62, 1.04), with substantial between-study heterogeneity (I^2=86%; P<0.001) (**Table 2 & eFigure 2**). The

For peer review only - http://bmjopen.bmj.com/site/about/guidelines.xhtml

multiple-adjusted RR of lung cancer associated with current smoking was 6.99 (95% CI: 5.09, 9.59) in women and 7.33 (95% CI: 4.90, 10.96) in men (**Table 2 & Figure 2**). The corresponding RRR was 0.92 (95% CI: 0.72, 1.16) and between-study heterogeneity was substantial (I^2=89%; P<0.001) (**Table 2 & Figure 3**). There was no evidence of publication bias based on the Begg's test (P=0.75) (**eFigure 3**).

The sex difference in the risk of smoking-related lung cancer in our main analysis did not differ in subgroup analyses stratified by the women-to-men ratio of current smokers (P=0.90), women-to-men ratio of lung cancer incidence in the studies (P=0.64), year of study baseline (P = 0.66), study endpoint (P=0.21) or study region (P=0.73) (**Table 3**). The sex difference in the risk of smoking-related lung cancer in our main analysis also did not differ by or follow-up time (P=0.83) or study quality (P=0.69). The RRR was 0.93 (95% CI: 0.72, 1.20) for studies from Asia and 0.87 (95% CI: 0.66, 1.14) for studies from USA, Europe, or Australia and New Zealand.

The risk of smoking-related lung cancer increased according to the number of cigarettes smoked per day in both sexes (**Table 2**). In women, the RRs were 5.30 (95% CI: 3.52, 7.97), 10.67 (95% CI: 7.43, 15.33), and 17.09 (95% CI: 12.11, 24.11) across subgroups of <10, 10 to 20, and >20 cigarettes per day versus non-smoking, respectively. Corresponding RRs in men were 4.97 (95% CI: 2.74, 9.03), 8.93 (95% CI: 4.90, 16.28), and 14.61 (95% CI: 8.33, 25.59), respectively. The RRRs in these subgroups were 0.99 (95% CI: 0.65, 1.52), 1.11 (95% CI: 0.75, 1.64), and 0.94 (95% CI: 0.69, 1.30), respectively.

Risk of lung cancer in former smokers versus never smokers

Data from 89 cohorts, including 6,006,725 individuals and 38,244 cases of lung cancer, reported on the risk of lung cancer in former smokers compared with never smokers. The age-adjusted RR of lung cancer associated with former smoking was 2.82 (95% CI: 2.25, 3.54) in women and 3.01 (95% CI: 2.23, 4.08) in men (**Table 2** & **eFigure 4**); the age-adjusted RRR was 0.88 (95% CI: 0.69, 1.14) (I^2=64%; P<0.001) (**Table 2** & **eFigure 5**). The corresponding multiple-adjusted RRs were 3.14 (95% CI:

For peer review only - http://bmjopen.bmj.com/site/about/guidelines.xhtml

2.45, 4.03) in women and 3.13 (95% CI: 2.06, 4.76) in men (**Table 2** & **eFigure6**). There was no statistical evidence that the effects of smoking cessation on risk of lung cancer differed between the sexes; the multiple-adjusted RRR was 0.89 (95% CI: 0.69, 1.13) (I^2=69%; P<0.001) (**Table 2** & **eFigure 7**). There was no evidence that the RRR differed across various subgroup analyses (**Table 3**).

For peer review only

For peer review only - http://bmjopen.bmj.com/site/about/guidelines.xhtml

Discussion

In this systematic review and meta-analysis, comprising data from more than seven million participants, 99 cohort studies and over 50,000 incident cases of lung cancer, there was no evidence for a difference in the risk of smoking-related lung cancer in women compared with men. This was true across a range of subgroup and sensitivity analyses. However, as smoking prevalence and intensity was higher in men compared with women in most studies included in this analysis, there may yet be an unrealised sex-difference in the risk of smoking-related lung cancer that will only become fully manifest as the smoking epidemic reaches full maturity in women.[2]

The seven-fold higher relative risks of lung cancer associated with smoking found in the present meta-analysis are considerably smaller than the 20-fold increased risks reported in the Million Women's Study (MWS)[13] and the British Doctors Study.[14] Both these studies had the advantage of capturing smoking-related risks in populations that had smoked for long enough for the effects to become fully manifest, highlighting the importance of taking into consideration the stage of the tobacco epidemic in each sex. The lack of any appreciable sex difference in the relative risks of lung cancer is surprising given men's greater cumulative exposure to smoking, in most populations, compared with women. In addition, men have a greater exposure to other risk factors for lung cancer including occupational carcinogens. [15] Men also smoke more cigars and pipes, [3] take longer puffs of longer duration and leave shorter butts compared with women.[4] Hence, it may be reasonable to surmise that the relative risk estimates of smoking-related lung cancer in women may eventually exceed those of men, once cumulative exposure to smoking in women is comparable to that in men. In a previous meta-analysis, using similar methodology, we found that smoking conferred a 25% greater relative risk of coronary heart disease (CHD) in women than in men. Two possible explanations for why a similar pattern is not observed for lung cancer are that, first, the lag-time of smoking associated with CHD is considerably shorter than for lung cancer[16], and second the pathways by which smoking increases risk are different between CHD and lung cancer.

For peer review only - http://bmjopen.bmj.com/site/about/guidelines.xhtml

Although not assessed in this analysis, evidence suggests that there are sex differences in the pattern of lung cancer among never smokers, with a higher prevalence of lung cancer among never-smoking women than never-smoking men.[17 18] A US study amongst 500,000 people found a 30% higher incidence of lung cancer in women never smokers compared with men never smokers.[19] An Australian study found the proportion of lung cancer patients who had never smoked was approximately 18% in women and 3% of men.[20] The reasons for this sex difference are not clear, but women may have increased exposure to environmental tobacco smoke[21] or other environmental carcinogens such as indoor air pollution[22], or sex-related differences in the metabolism of environmental carcinogens. The possibility of greater exposure to environmental tobacco smoke and other environmental carcinogens in women compared with men could have resulted in a greater underestimation of the association between smoking and lung cancer in women than men. This, in turn, could have impacted the sex difference in risk of smoking-related lung cancer reported in this study.

Our study has several strengths including restriction to cohort studies which provide more robust evidence of the associations compared with case-control studies. Differences between case-control and cohort studies may also explain why a previous meta-analysis of case-control studies (which included only 3 cohort studies) showed higher relative risk of lung cancer in men compared with women.[10] Other strengths to our study include an update of findings to include studies published after 1999,[11] with supplementation of published literature by individual participant data from three established population databases. We have also performed a range of pre-specified sensitivity analyses and several subgroup analyses which were not performed in previous meta-analyses. Our results were consistent across regions and irrespective of the women-to-men smoking ratio, suggesting that underestimation of the association of smoking with lung cancer in women due to sex differences in smoking prevalence and underreporting of smoking is unlikely. This is especially

For peer review only - http://bmjopen.bmj.com/site/about/guidelines.xhtml

relevant for parts of Asia where the prevalence of smoking in women is typically <10% and where smoking among women remains relatively socially unacceptable. As the up-take of smoking continues among women in countries where significant sex differences in smoking prevalence exist, the sex-specific risks of lung cancer due to smoking may become further apparent. This is also true in regards to Western countries where differences in prevalence between women and men have reduced substantially over time, with prevalence of smoking in younger cohorts of women and men approaching unity.[23] The limitations of this study include heterogeneity across studies in study design, study population, verification of smoking status, and outcome ascertainment. Assessment of smoking status differed across studies and was generally self-reported, which may have introduced measurement error.[24] Notably, compared with men, women typically are more likely to underreport smoking status, and underreporting is especially prevalent in countries where female smoking is not culturally acceptable.[25] The lack of standardisation across studies in how smoking status was obtained, including how smoking dose and duration were measured is also a major limitation. In addition, there was insufficient data available to examine whether there were sex differences in the impact of age at smoking initiation and smoking duration on the risk of lung cancer. The reference group of non-smokers in our analysis of current smoking was composed of former and never smokers which may inflate the risks of smoking-related lung cancer risk among non-smokers. However, we have also examined former smoking compared with never smoking and demonstrated no appreciable sex differences in the risks of smoking-related lung cancer in this group, which provides some evidence that the inclusion of former smokers in the reference category is unlikely to have biased the sex difference in our main analysis. We quantified sex differences in the risk of lung cancer associated with smoking based on RRs rather than absolute risks. This might introduce a statistical artefact, in which the generally higher absolute risk for lung cancer in men, and the same risk difference subsequent to smoking in each sex, would translate to a greater relative risk in women than men. However, our previous meta-analyses on risk factors for cardiovascular diseases demonstrated that sex differences in RRs are not inevitable[26], despite differences in absolute risks.

For peer review only - http://bmjopen.bmj.com/site/about/guidelines.xhtml

Compared with absolute risks, RRs are more stable across populations with different background risks, which makes them suitable for meta-analyses. In addition, RRs are reported much more commonly than absolute risks. In our review, no studies reported adjusted absolute risks, with standard errors, that allow for meta-analyses. We therefore believe that usage of RRs in the present analysis is appropriate. In addition, while we have aimed to assess study quality using the widely accepted and used Newcastle-Ottawa Scale, the value and contribution of quality assessment scales such as this to systematic reviews and meta-analyses continues to be debated. [27-29] Finally, there are differences between men and women in histological subtypes of lung cancer. Adenocarcinoma is more common in women and squamous cell carcinoma is more common in men. [30] Smoking is more strongly associated with squamous cell carcinoma than adenocarcinoma. [30] Few studies reported the sex-specific association of smoking with histological subtypes of cancer, which precluded the examination of sex differences in the association of smoking-related lung cancer subtypes and this remains an important limitation of our review. [30] Further studies of the smoking-related risks of lung cancer in women and men are required as the smoking epidemic reaches its full maturity in women. Given the later up-take of smoking in women, studies which allow sufficient lag time for lung cancer to develop are essential. In addition, reducing under-reporting of smoking in women, using standardised and robust methods for the ascertainment of smoking status and smoking behaviours and more extensive measurement and adjustment for confounders which differ by sex (such as exposure to environmental tobacco smoke) is also important for future work, as well as examination of histological subtypes of lung cancer which was not possible in this review.

In conclusion, this meta-analysis, summarising all available literature to date, shows that the effect of smoking on risk of lung cancer is similar in women and men. However, these data may yet underestimate the true relative risk of smoking in women, given later uptake and lower intensity of smoking in women. Although strides have been made in reducing smoking rates particularly in high-income countries, continuing efforts to measure the effects of smoking on disease outcomes are

For peer review only - http://bmjopen.bmj.com/site/about/guidelines.xhtml

required, as the smoking epidemic has not yet reached its global peak, particularly among women. In addition, tobacco control programmes that dissuade smokers of both sexes from smoking but which also encourage individuals to quit remain a priority.

For peer review only

For peer review only - http://bmjopen.bmj.com/site/about/guidelines.xhtml

Figure 1: Flow chart of study selection

Figure 2: Multiple-adjusted relative risk for incident lung cancer in women and men, comparing current smokers to non-smokers

Legend: APCSC, Asia Pacific Cohort Studies Collaboration; ARIC, Atherosclerosis Risk in Communities study; CPS, Cancer Prevention Study; EHS, Elderly Health Services; EPIC, European Prospective Investigation into Cancer; JACC, Japan Collaborative Cohort Study; JPHC, Japan Public Health Centre study; NHANES III, National Health And Nutrition Examination Survey III; NHIS, National Health Interview Survey; NIH-AARP, NIH-AARP Diet and Health Study ; SHHEC, Scottish Heart Health Extended Cohort Study; TPCS, Three-Prefecture Cohort Study. Multiple-adjusted includes anything that adjusted for more than just age. These covariates are listed in Table 1.Figures may contain less than 29 studies because we report age- and multiple-adjusted results separately. Some studies only contributed age-adjusted results whereas others only provided multiple-adjusted results. However, the count of unique studies that contributed to at least one of these analyses is 29.

Figure 3: Multiple-adjusted women-to-men ratio of relative risks for incident lung cancer, comparing current smokers to non-smokers

Legend: APCSC, Asia Pacific Cohort Studies Collaboration; ARIC, Atherosclerosis Risk in Communities study; CPS, Cancer Prevention Study; EHS, Elderly Health Services; EPIC, European Prospective Investigation into Cancer; JACC, Japan Collaborative Cohort Study; JPHC, Japan Public Health Centre study; NHANES III, National Health And Nutrition Examination Survey III; NHIS, National Health Interview Survey; NIH-AARP, NIH-AARP Diet and Health Study ; SHHEC, Scottish Heart Health Extended Cohort Study; TPCS, Three-Prefecture Cohort Study. Multiple-adjusted includes anything that adjusted for more than just age. These covariates are listed in Table 1.Figures may contain less than 29 studies because we report age- and multiple-adjusted results separately. Some studies only contributed age-adjusted results whereas others only provided multiple-adjusted results. However, the count of unique studies that contributed to at least one of these analyses is 29.

For peer review only

For peer review only - http://bmjopen.bmj.com/site/about/guidelines.xhtml

Table 1: Characteristics of included studies

Study name *	Location	Age range, (y)	Baseline year	Follow-up, (years)	NOS score (points)	N (%) women	N (%) lung cancer in women	Fatal/ non-fatal	Current smoker, % W	Current smoker, % M	Former smoker, % W	Former smoker, % M	Maximum available adjustment available
**APCSC – ANZ[31] (8 cohorts)**	ANZ	20-107	1961-93	10	6	87,130 (52)	501 (28)	F	7	11	11	18	Age, education, BMI
**APCSC - Asia[31] (33 cohorts)**	Asia	20-104	1989-96	7	6	476,755 (35)	1,275 (19)	F	2	59	<1	7	Age, education, BMI
**ARIC[32] (1 cohort)**	USA	45-64	1987-89	20	8	14,610 (54)	470 (37)	F/NF	25	44	22	28	Age, race
**China Kadoorie Biobank[33 34] (1 cohort)**	China	35-74	2004-08	7	7	512,891 (59)	1,953 (37)	F	3	61	1	15	Age, area, education, alcohol
**China National Hypertension Survey[35] (1 cohort)**	China	15+	1991	8	7	155,131 (51)	NR	F	14	63	NR	NR	Age, education, region, HT, overweight/obesity, alcohol, PA, DM
**Copenhagen Cohort Studies[36] (3 cohorts)**	Denmark	20+	1964-92	14	6	30,874 (44)	867 (23)	F/NF	NR	NR	NR	NR	Age
**CPS I (1 cohort)**	USA	55+	1959	6	5	518,982 (65)	1,293 (21)	F	15	40	4	17	Age, education, race
**CPS II[37] (1 cohort)**	USA	55+	1982	6	5	746,485 (61)	4,957 (36)	F	18	24	21	43	Age, education, race
**EHS[38 39] (1 cohort)**	China	65+	1998-00	11	7	65,510 (66)	1,096 (27)	F	4	10	8	20	Age, education, alcohol, depression, health status, social security assistance, housing type, expenditure
**EPIC[40] (23 cohorts)**	Europe	30-70	1992-00	11	8	441,211 (70)	2,995 (49)	F/NF	21	27	25	38	Age, education, BMI, alcohol, PA, energy intake, diet
**JPHC[41], JACC, TPCS[37 42 43] (3 cohorts)**	Japan	40-79	1983-94	10	6	296,836 (53)	NR	F	8	54	2	25	Age
**Korean Cancer Prevention Study[44] (1 cohort)**	Korea	30-95	1993-95	9	6	1,212,906 (32)	4,238 (14)	F	5	57	3	23	Age
**Korean National Health Insurance Service[8] (1 cohort)**	Korea	20+	1998-99	10	7	1,355,891 (31)	6,491 (15)	F/NF	1	56	1	13	Age, BMI, alcohol, PA
**Malmo Preventive Project[45] (1 cohort)**	Sweden	27-61	1974-92	24	5	33,346 (33)	436 (21)	F	36	49	19	27	Age, FEV, SES, marital status
**Migrant Study[46] (1 cohort)**	Norway	33-72	1964-65	21	7	26,126 (55)	435 (23)	F	26	46	8	28	Age
**New Zealand Census 1981[47] (1 cohort)**	New Zealand	25+	1981	5	7	NR	4,188 (28)	F/NF	NR	NR	NR	NR	Age, race
**New Zealand Census 1996[47] (1 cohort)**	New Zealand	25+	1996	5	7	NR	4,467 (44)	F/NF	NR	NR	NR	NR	Age, race
**NHANES III (1 cohort)**	USA	18-90	1988-94	13	7	20,006 (53)	320 (36)	F	21	30	17	32	Age, education, BMI
**NHIS[48] (1 cohort)**	USA	25+	1997-04	7	7	202,248 (56)	1,223 (44)	F	21	26	20	29	Age, education, BMI, alcohol
**NIH-AARP[7 49] (1 cohort)**	USA	50-71	1995-96	11	7	452,131 (41)	9,381 (37)	F/NF	17	13	39	61	Age, education, alcohol, ethnicity
**Norwegian Counties Study[50-52] (1 cohort)**	Norway	20-49	1974-78	23	6	48,682 (48)	686 (35)	F	43	50	11	18	Age, SBP, TC, TG, PA, BMI, height, sickness leave, disability pension, family history of CHD
**Renfrew/Paisley Study[53] (1 cohort)**	Scotland	45-64	1972-76	20	6	15,393 (54)	NR	F	47	59	7	25	Age
**Reykjavik Study[54] (1 cohort)**	Iceland	32-60	1967	27	7	22,946 (50)	472 (42)	F/NF	39	30	15	24	Age
**Shanghai Health Study[55] (2 cohorts)**	China	40-74	1997-06	10	7	134,335 (54)	839 (49)	F	3	70	NR	NR	Age, education, BMI, alcohol, PA, income

For peer review only - http://bmjopen.bmj.com/site/about/guidelines.xhtml

**SHHEC (1 cohort)**	Scotland	25-75	1984-87	23	6	17,731 (51)	558 (38)	F	40	51	21	27	Age, education, BMI
**Singapore Chinese Health Study**[56] **(1 cohort)**	Singapore	45-74	1993-98	12	8	61,320 (55)	905 (31)	F/NF	6	36	3	21	Age, dialect, year of recruitment, education, alcohol, PA
**Swedish Smoking Habit Survey**[57 58] **(1 cohort)**	Sweden	18-69	1963	33	8	41,544 (60)	442 (45)	F	18	27	5	23	Age, area
**Swiss National Cohort** [59] **(4 cohorts)**	Switzerland	14-99	1977-93	19	5	35,703 (53)	426 (29)	F	27	43	13	24	Age, survey, education, alcohol, PA, civil status, nationality, diet
**Wen**[60] **(2 cohorts)**	Taiwan	35+	1982-92	20	5	86,580 (39)	247 (2)	F	1	41	<1	11	Age

APCSC, Asia Pacific Cohort Studies Collaboration; ANZ, Australia and New-Zealand, ARIC, Atherosclerosis Risk in Communities study; BMI, body mass index; CPS, Cancer Prevention Study; CHD, coronary heart disease; DM, diabetes mellitus; EHS, Elderly Health Services; EPIC, European Prospective Investigation into Cancer; F, fatal; FEV, forced expiratory volume, JACC, Japan Collaborative Cohort study; JPHC, Japan Public Health Centre study; NF, non-fatal; NHANES III, National Health And Nutrition Examination Survey III; NHIS, National Health Interview Survey ; NIH-AARP, NIH-AARP Diet and Health Study ; NOS, Newcastle Ottawa Scale; NR, not reported; PA, physical activity; SES, socioeconomic status; SHHEC, Scottish Heart Health Extended Cohort Study; SBP, systolic blood pressure; TC, total cholesterol; TG, triglycerides; TPCS, Three-Prefecture Cohort Study

*Note that where several studies are cited for a single cohort, data may be extracted from multiple studies if all data required is not available in the most up-to-date and relevant study.

.

For peer review only

For peer review only - http://bmjopen.bmj.com/site/about/guidelines.xhtml

Table 2: Sex-specific pooled relative risks and ratio of relative risks for lung cancer associated with smoking

		RR in women	RR in men	Ratio of RR
Age-adjusted	Former vs. never	2.82 (2.25, 3.54)	3.01 (2.23, 4.08)	0.88 (0.69, 1.14)
	Current vs. not	7.48 (5.29. 10.60)	8.78 (6.13, 12.57)	0.81 (0.62, 1.04)
Multiple-adjusted	Former vs. never	3.14 (2.45, 4.03)	3.13 (2.06, 4.76)	0.89 (0.69, 1.13)
	Current vs. not	6.99 (5.09, 9.59)	7.33 (4.90, 10.96)	0.92 (0.72, 1.16)
Maximum-adjusted	Former vs. never	2.92 (2.35, 3.63)	3.08 (2.31, 4.11)	0.86 (0.71, 1.05)
	Current vs. not	7.32 (5.58, 9.61)	8.05 (5.90, 10.98)	0.89 (0.73, 1.08)
**Cigarettes per day among current smokers vs. never (maximum available-adjusted)**				
	10 or less	5.30 (3.52, 7.97)	4.97 (2.74, 9.03)	0.99 (0.65, 1.52)
	10 to 19	10.67 (7.43, 15.33)	8.93 (4.90, 16.28)	1.11 (0.75, 1.64)
	20 or more	17.09 (12.11, 24.11)	14.61 (8.33, 25.59)	0.94 (0.69, 1.30)

Multiple-adjusted includes anything that adjusted for more than just age. Maximum available adjustment refers to the most adjustments provided in the study. For some studies, this would have been age-adjusted whereas other studies adjusted for more factors than age only (i.e. multiple-adjusted). These covariates are listed in Table 1.

For peer review only - http://bmjopen.bmj.com/site/about/guidelines.xhtml

Table 3: Maximally adjusted sex difference in the association of smoking with lung cancer, in subgroup analyses

	N studies	Former vs. never	P for interaction	N studies	Current vs. not	P for interaction
**Study region**						
Asia	40	1.07 (0.83, 1.37)		46	0.93 (0.72, 1.20)	
Non-Asia	49	0.73 (0.58, 0.93)	0.06	53	0.87 (0.66, 1.14)	0.73
**Study region**						
Asia	40	1.07 (0.83, 1.37)		46	0.93 (0.72, 1.20)	
USA	5	0.60 (0.42, 0.84)		6	0.58 (0.37, 0.91)	
Europe	36	0.81 (0.60, 1.10)		37	0.99 (0.63, 1.57)	
ANZ	8	1.41 (0.65, 3.04)	0.55	10	1.11 (0.97, 1.28)	0.69
**Year of study baseline**						
1985 or before	23	0.79 (0.56, 1.12)		25	0.96 (0.66, 1.40)	
After 1985	66	0.92 (0.73, 1.16)	0.43	74	0.85 (0.68, 1.06)	0.66
**Women to men smoking prevalence**						
>67% lower in women	39	1.16 (0.83, 1.62)		43	0.96 (0.72, 1.28)	
33-67% lower in women	19	0.72 (0.51, 1.03)		20	0.75 (0.50, 1.14)	
0-33% lower in women	28	0.78 (0.59, 1.03)	0.26	29	0.97 (0.59, 1.58)	0.90
**Women to men lung cancer rate**						
>= 50% lower in women	80	0.85 (0.65, 1.10)		83	0.85 (0.62, 1.17)	
0- 50% lower in women	6	0.83 (0.61, 1.13)	0.79	9	0.94 (0.68, 1.31)	0.64
**Study endpoint**						
Fatal lung cancer only	57	0.94 (0.68, 1.29)		67	0.97 (0.77, 1.21)	
Fatal and non-fatal lung cancer	32	0.80 (0.64, 1.01)	0.57	32	0.72 (0.48, 1.06)	0.21
**Duration of follow-up**						
≤ 10 yrs	48	0.90 (0.60, 1.35)		53	0.91(0.68, 1.24)	
>10 yrs	41	0.85 (0.71, 1.05)	0.92	46	0.87 (0.67, 1.12)	0.83
**Study quality**						
≤ 6 points	58	0.85 (0.64, 1.13)		61	0.84 (0.53, 1.12)	
>6 points	31	0.89 (0.66, 1.20)	0.81	38	0.93 (0.72, 1.20)	0.69

Random effects meta-analysis were used for all subgroup analyses and differences between subgroups were examined using meta-regression.

For peer review only - http://bmjopen.bmj.com/site/about/guidelines.xhtml

References

1. Institute for Health Metrics and Evaluation. Global Burden of Disease 2015 2015 [Available from: http://vizhub.healthdata.org/gbd-compare/#
2. Reitsma MB, Fullman N, Ng M, et al. Smoking prevalence and attributable disease burden in 195 countries and territories, 1990–2015: a systematic analysis from the Global Burden of Disease Study 2015. *The Lancet* 2017
3. Nelson DE, Davis RM, Chrismon JH, et al. Pipe smoking in the United States, 1965–1991: prevalence and attributable mortality. *Preventive medicine* 1996;25(2):91-99.
4. Melikian AA, Djordjevic MV, Hosey J, et al. Gender differences relative to smoking behavior and emissions of toxins from mainstream cigarette smoke. *Nicotine & Tobacco Research* 2007;9(3):377-87.
5. Becklake MR, Kauffmann F. Gender differences in airway behaviour over the human life span. *Thorax* 1999;54(12):1119-38.
6. Huxley RR, Woodward M. Cigarette smoking as a risk factor for coronary heart disease in women compared with men: a systematic review and meta-analysis of prospective cohort studies. *The Lancet* 2011;378(9799):1297-305.
7. Thun MJ, Carter BD, Feskanich D, et al. 50-year trends in smoking-related mortality in the United States. *New England Journal of Medicine* 2013;368(4):351-64.
8. Yun YD, Back JH, Ghang H, et al. Hazard ratio of smoking on lung cancer in Korea according to histological type and gender. *Lung* 2016;194(2):281-89.
9. Powell HA, Iyen-Omofoman B, Hubbard RB, et al. The association between smoking quantity and lung cancer in men and women. *CHEST Journal* 2013;143(1):123-29.
10. Yu Y, Liu H, Zheng S, et al. Gender susceptibility for cigarette smoking-attributable lung cancer: A systematic review and meta-analysis. *Lung cancer* 2014;85(3):351-60.
11. Lee PN, Forey BA, Coombs KJ. Systematic review with meta-analysis of the epidemiological evidence in the 1900s relating smoking to lung cancer. *BMC cancer* 2012;12(1):385.
12. G W, B S, D OC, et al. The Newcastle-Ottawa Scale (NOS) for Assessing the Quality of Nonrandomised Studies in Meta-analyses Ottawa Ottawa Hospital Research Institute; 2012 [
13. Pirie K, Peto R, Reeves GK, et al. The 21st century hazards of smoking and benefits of stopping: a prospective study of one million women in the UK. *The Lancet* 2013;381(9861):133-41.
14. Doll R, Peto R, Boreham J, et al. Mortality in relation to smoking: 50 years' observations on male British doctors. *BMJ (Clinical research ed)* 2004;328(7455):1519.
15. Olsson AC, Gustavsson P, Zaridze D, et al. Lung cancer risk attributable to occupational exposures in a multicenter case-control study in Central and Eastern Europe. *Journal of occupational and environmental medicine* 2011;53(11):1262-67.
16. Ockene JK, Kuller LH, Svendsen KH, et al. The relationship of smoking cessation to coronary heart disease and lung cancer in the Multiple Risk Factor Intervention Trial (MRFIT). *American journal of public health* 1990;80(8):954-58.
17. Zang EA, Wynder EL. Differences in lung cancer risk between men and women: examination of the evidence. *Journal of the National Cancer Institute* 1996;88(3-4):183-92.
18. Thun MJ, Hannan LM, Adams-Campbell LL, et al. Lung cancer occurrence in never-smokers: an analysis of 13 cohorts and 22 cancer registry studies. *PLoS medicine* 2008;5(9):e185.
19. Freedman ND, Leitzmann MF, Hollenbeck AR, et al. Cigarette smoking and subsequent risk of lung cancer in men and women: analysis of a prospective cohort study. *The lancet oncology* 2008;9(7):649-56.
20. Mitchell P, Thursfield VJ, Ball DL, et al. Lung cancer in Victoria: are we making progress. *The Medical journal of Australia* 2013;199(10):674-9.
21. Sisti J, Boffetta P. What proportion of lung cancer in never-smokers can be attributed to known risk factors? *International journal of cancer* 2012;131(2):265-75.

For peer review only - http://bmjopen.bmj.com/site/about/guidelines.xhtml

22. Hosgood III HD, Boffetta P, Greenland S, et al. In-home coal and wood use and lung cancer risk: a pooled analysis of the International Lung Cancer Consortium. *Environmental health perspectives* 2010;118(12):1743.
23. Peters SA, Huxley RR, Woodward M. Do smoking habits differ between women and men in contemporary Western populations? Evidence from half a million people in the UK Biobank study. *BMJ open* 2014;4(12):e005663.
24. Patrick DL, Cheadle A, Thompson DC, et al. The validity of self-reported smoking: a review and meta-analysis. *American journal of public health* 1994;84(7):1086-93.
25. Jung-Choi K-H, Khang Y-H, Cho H-J. Hidden female smokers in Asia: a comparison of self-reported with cotinine-verified smoking prevalence rates in representative national data from an Asian population. *Tobacco Control* 2012;21(6):536-42.
26. Huxley RR, Peters SA, Mishra GD, et al. Risk of all-cause mortality and vascular events in women versus men with type 1 diabetes: a systematic review and meta-analysis. *The Lancet Diabetes & Endocrinology* 2015;3(3):198-206.
27. Katikireddi SV, Egan M, Petticrew M. How do systematic reviews incorporate risk of bias assessments into the synthesis of evidence? A methodological study. *Journal of epidemiology and community health* 2014:jech-2014-204711.
28. Viswanathan M, Berkman ND, Dryden DM, et al. Assessing risk of bias and confounding in observational studies of interventions or exposures: further development of the RTI item bank. 2013
29. Sanderson S, Tatt ID, Higgins J. Tools for assessing quality and susceptibility to bias in observational studies in epidemiology: a systematic review and annotated bibliography. *International journal of epidemiology* 2007;36(3):666-76.
30. Devesa SS, Bray F, Vizcaino AP, et al. International lung cancer trends by histologic type: male: female differences diminishing and adenocarcinoma rates rising. *International journal of cancer* 2005;117(2):294-99.
31. Huxley R, Jamrozik K, Lam TH, et al. Impact of smoking and smoking cessation on lung cancer mortality in the Asia-Pacific region. *American journal of epidemiology* 2007;165(11):1280-86.
32. Prizment AE, Yatsuya H, Lutsey PL, et al. Smoking behavior and lung cancer in a biracial cohort: The atherosclerosis risk in communities study. *American journal of preventive medicine* 2014;46(6):624-32.
33. Chen Z, Peto R, Zhou M, et al. Contrasting male and female trends in tobacco-attributed mortality in China: evidence from successive nationwide prospective cohort studies. *The Lancet* 2015;386(10002):1447-56.
34. Chen ZM, Peto R, Iona A, et al. Emerging tobacco-related cancer risks in China: A nationwide, prospective study of 0.5 million adults. *Cancer* 2015;121(S17):3097-106.
35. Gu D, Kelly TN, Wu X, et al. Mortality attributable to smoking in China. *New England Journal of Medicine* 2009;360(2):150-59.
36. Prescott E, Osier M, Rein HO, et al. Gender and smoking-related risk of lung cancer. *Epidemiology (Cambridge, Mass)* 1998;9(1):79-83.
37. Marugame T, Sobue T, Satoh H, et al. Lung cancer death rates by smoking status: Comparison of the Three-Prefecture Cohort study in Japan to the Cancer Prevention Study II in the USA. *Cancer science* 2005;96(2):120-26.
38. Lam TH, Li ZB, Ho SY, et al. Smoking, quitting and mortality in an elderly cohort of 56 000 Hong Kong Chinese. *Tobacco control* 2007;16(3):182-89.
39. Lam TH, Xu L, Schooling CM, et al. Smoking and mortality in a prospective cohort study of elderly Chinese in Hong Kong. *Addiction* 2015;110(3):502-10.
40. Agudo A, Bonet C, Travier N, et al. Impact of cigarette smoking on cancer risk in the European prospective investigation into cancer and nutrition study. *Journal of Clinical Oncology* 2012;30(36):4550-57.

For peer review only - http://bmjopen.bmj.com/site/about/guidelines.xhtml

41. Sobue T, Yamamoto S, Hara M, et al. Cigarette smoking and subsequent risk of lung cancer by histologic type in middle-aged Japanese men and women: The JPHC study. *International journal of cancer* 2002;99(2):245-51.
42. Akiba S, Hirayama T. Cigarette smoking and cancer mortality risk in Japanese men and women--results from reanalysis of the six-prefecture cohort study data. *Environmental health perspectives* 1990;87:19.
43. Katanoda K, Marugame T, Saika K, et al. Population attributable fraction of mortality associated with tobacco smoking in Japan: a pooled analysis of three large-scale cohort studies. *Journal of epidemiology* 2008;18(6):251-64.
44. Jee SH, Samet JM, Ohrr H, et al. Smoking and cancer risk in Korean men and women. *Cancer Causes & Control* 2004;15(4):341-48.
45. Ekberg-Aronsson M, Nilsson PM, Nilsson J-Å, et al. Mortality risks among heavy-smokers with special reference to women: a long-term follow-up of an urban population. *European journal of epidemiology* 2007;22(5):301-09.
46. Engeland A, Haldorsen T, Andersen A, et al. The impact of smoking habits on lung cancer risk: 28 years' observation of 26,000 Norwegian men and women. *Cancer Causes and Control* 1996;7(3):366-76.
47. Blakely T, Barendregt JJ, Foster RH, et al. The association of active smoking with multiple cancers: national census-cancer registry cohorts with quantitative bias analysis. *Cancer Causes & Control* 2013;24(6):1243-55.
48. Jha P, Ramasundarahettige C, Landsman V, et al. 21st-century hazards of smoking and benefits of cessation in the United States. *New England Journal of Medicine* 2013;368(4):341-50.
49. Freedman ND, Abnet CC, Caporaso NE, et al. Impact of changing US cigarette smoking patterns on incident cancer: risks of 20 smoking-related cancers among the women and men of the NIH-AARP cohort. *International journal of epidemiology* 2016;45(3):846-56.
50. Bjartveit K, Tverdal A. Health consequences of sustained smoking cessation. *Tobacco control* 2009;18(3):197-205.
51. Bjartveit K, Tverdal A. Health consequences of smoking 1–4 cigarettes per day. *Tobacco control* 2005;14(5):315-20.
52. Vollset SE, Tverdal A, Gjessing HkK. Smoking and deaths between 40 and 70 years of age in women and men. *Annals of internal medicine* 2006;144(6):381-89.
53. Marang-van de Mheen PJ, Smith GD, Hart CL, et al. Are women more sensitive to smoking than men? Findings from the Renfrew and Paisley study. *International journal of epidemiology* 2001;30(4):787-92.
54. Tulinius H, Sigfusson N, Sigvaldason H, et al. Risk factors for malignant diseases: a cohort study on a population of 22,946 Icelanders. *Cancer epidemiology, biomarkers & prevention : a publication of the American Association for Cancer Research, cosponsored by the American Society of Preventive Oncology* 1997;6(11):863-73. [published Online First: 1997/11/21]
55. Wang Y-Y, Zhang W, Li H-L, et al. Population attributable risks of cigarette smoking for deaths of all causes, all cancers and other chronic diseases among adults aged 40-74 years in urban Shanghai, China. *Chinese Journal of Cancer Research* 2015;27(1):59.
56. Shankar A, Yuan J-M, Koh W-P, et al. Morbidity and mortality in relation to smoking among women and men of Chinese ethnicity: the Singapore Chinese Health Study. *European journal of cancer* 2008;44(1):100-09.
57. Nilsson S, Carstensen J, Pershagen G. Mortality among male and female smokers in Sweden: a 33 year follow up. *Journal of epidemiology and community health* 2001;55(11):825-30.
58. Nordlund LA, Carstensen JM, Pershagen G. Are male and female smokers at equal risk of smoking-related cancer: evidence from a Swedish prospective study. *Scandinavian journal of public health* 1999;27(1):56-62.

For peer review only - http://bmjopen.bmj.com/site/about/guidelines.xhtml

59. Meyer J, Rohrmann S, Bopp M, et al. Impact of smoking and excess body weight on overall and site-specific cancer mortality risk. *Cancer Epidemiology and Prevention Biomarkers* 2015;24(10):1516-22.
60. Wen C-P, Tsai SP, Chen C-J, et al. The mortality risks of smokers in Taiwan: Part I: cause-specific mortality. *Preventive medicine* 2004;39(3):528-35.

For peer review only

For peer review only - http://bmjopen.bmj.com/site/about/guidelines.xhtml

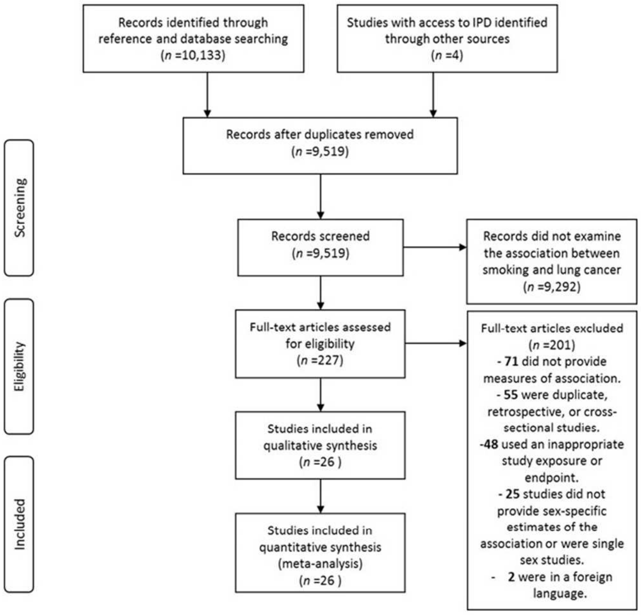

Figure 1: Flow diagram of study selection

90x90mm (300 x 300 DPI)

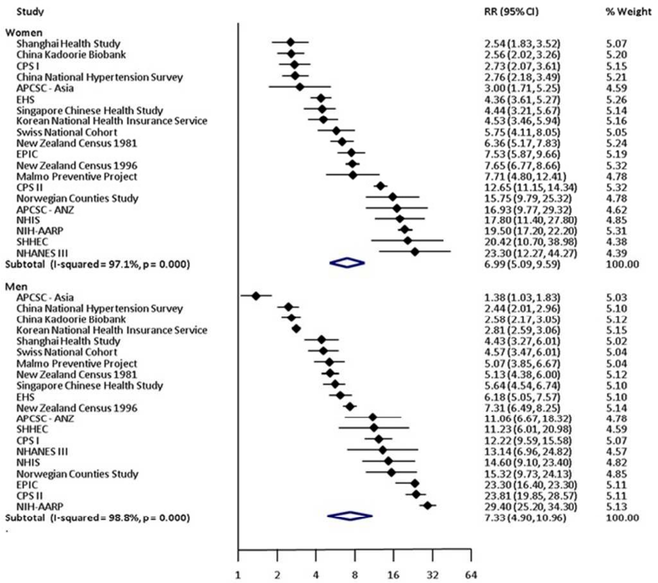

Figure 2: Multiple-adjusted relative risk for incident lung cancer in women and men, comparing current smokers to non-smokers

90x90mm (300 x 300 DPI)

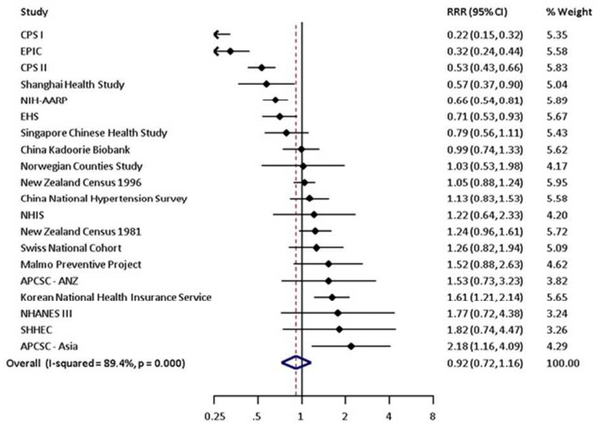

Figure 3: Multiple-adjusted women-to-men ratio of relative risks for incident lung cancer, comparing current smokers to non-smokers

90x90mm (300 x 300 DPI)

Supplementary Material

Smoking as a risk factor for lung cancer in women and men: a systematic review and meta-analysis

eAppendix 1: MOOSE checklist

eAppendix 2: Details of search strategy

eAppendix3: Quality assessment checklist

eTable 1: Quality assessment of the included studies

eFigure 1: Age-adjusted relative risk for incident lung cancer in women and men, comparing current smokers to non-smokers

eFigure 2: Age-adjusted women-to-men ratio of relative risks for incident lung cancer, comparing current smokers to non-smokers

eFigure 3: Contour funnel plots with pseudo 95% confidence limits for the ratio of relative risks associated with current and former smoking

eFigure 4: Age-adjusted relative risk for incident lung cancer in women and men, comparing former smokers to never smokers

eFigure 5: Age-adjusted women-to-men ratio of relative risks for incident lung cancer, comparing former smokers to never smokers

eFigure 6: Multiple-adjusted relative risk for incident lung cancer in women and men, comparing former smokers to never smokers

eFigure 7: Multiple-adjusted women-to-men ratio of relative risks for incident lung cancer, comparing former smokers to never smokers

For peer review only

eAppendix 1: MOOSE checklist

Item No	Recommendation	Reported on Page No
Reporting of background should include		
1	Problem definition	4
2	Hypothesis statement	4
3	Description of study outcome(s)	4
4	Type of exposure or intervention used	4
5	Type of study designs used	4
6	Study population	4
Reporting of search strategy should include		
7	Qualifications of searchers (eg, librarians and investigators)	6
8	Search strategy, including time period included in the synthesis and key words	6
9	Effort to include all available studies, including contact with authors	6
10	Databases and registries searched	6
11	Search software used, name and version, including special features used (eg, explosion)	6
12	Use of hand searching (eg, reference lists of obtained articles)	6
13	List of citations located and those excluded, including justification	13
14	Method of addressing articles published in languages other than English	6
15	Method of handling abstracts and unpublished studies	6
16	Description of any contact with authors	6
Reporting of methods should include		
17	Description of relevance or appropriateness of studies assembled for assessing the hypothesis to be tested	6&7
18	Rationale for the selection and coding of data (eg, sound clinical principles or convenience)	6&7
19	Documentation of how data were classified and coded (eg, multiple raters, blinding and interrater reliability)	6&7

For peer review only

For peer review only - http://bmjopen.bmj.com/site/about/guidelines.xhtml

20	Assessment of confounding (eg, comparability of cases and controls in studies where appropriate)	6&7
21	Assessment of study quality, including blinding of quality assessors, stratification or regression on possible predictors of study results	6
22	Assessment of heterogeneity	7
23	Description of statistical methods (eg, complete description of fixed or random effects models, justification of whether the chosen models account for predictors of study results, dose-response models, or cumulative meta-analysis) in sufficient detail to be replicated	7
24	Provision of appropriate tables and graphics	13-18
Reporting of results should include		
25	Graphic summarizing individual study estimates and overall estimate	14&15
26	Table giving descriptive information for each study included	12
27	Results of sensitivity testing (eg, subgroup analysis)	18
28	Indication of statistical uncertainty of findings	8&9

For peer review only

For peer review only - http://bmjopen.bmj.com/site/about/guidelines.xhtml

Item No	Recommendation	Reported on Page No
Reporting of discussion should include		
29	Quantitative assessment of bias (eg, publication bias)	eTable7
30	Justification for exclusion (eg, exclusion of non-English language citations)	6
31	Assessment of quality of included studies	eTable1
Reporting of conclusions should include		
32	Consideration of alternative explanations for observed results	10
33	Generalization of the conclusions (ie, appropriate for the data presented and within the domain of the literature review)	10
34	Guidelines for future research	11
35	Disclosure of funding source	3

From: Stroup DF, Berlin JA, Morton SC, et al, for the Meta-analysis Of Observational Studies in Epidemiology (MOOSE) Group. Meta-analysis of Observational Studies in Epidemiology. A Proposal for Reporting. *JAMA*. 2000;283(15):2008-2012. doi: 10.1001/jama.283.15.2008.

Transcribed from the original paper within the NEUROSURGERY® Editorial Office, Atlanta, GA, United Sates. August 2012.

For peer review only

eAppendix 1: MOOSE checklist

Item No	Recommendation	Reported on Page No
Reporting of background should include		
1	Problem definition	4
2	Hypothesis statement	4
3	Description of study outcome(s)	4
4	Type of exposure or intervention used	4
5	Type of study designs used	4
6	Study population	4
Reporting of search strategy should include		
7	Qualifications of searchers (eg, librarians and investigators)	6
8	Search strategy, including time period included in the synthesis and key words	6
9	Effort to include all available studies, including contact with authors	6
10	Databases and registries searched	6
11	Search software used, name and version, including special features used (eg, explosion)	6
12	Use of hand searching (eg, reference lists of obtained articles)	6
13	List of citations located and those excluded, including justification	13
14	Method of addressing articles published in languages other than English	6
15	Method of handling abstracts and unpublished studies	6
16	Description of any contact with authors	6
Reporting of methods should include		
17	Description of relevance or appropriateness of studies assembled for assessing the hypothesis to be tested	6&7
18	Rationale for the selection and coding of data (eg, sound clinical principles or convenience)	6&7
19	Documentation of how data were classified and coded (eg, multiple raters, blinding and interrater reliability)	6&7
20	Assessment of confounding (eg, comparability of cases and controls in studies	6&7

For peer review only

For peer review only - http://bmjopen.bmj.com/site/about/guidelines.xhtml

	where appropriate)	
21	Assessment of study quality, including blinding of quality assessors, stratification or regression on possible predictors of study results	6
22	Assessment of heterogeneity	7
23	Description of statistical methods (eg, complete description of fixed or random effects models, justification of whether the chosen models account for predictors of study results, dose-response models, or cumulative meta-analysis) in sufficient detail to be replicated	7
24	Provision of appropriate tables and graphics	13-18
Reporting of results should include		
25	Graphic summarizing individual study estimates and overall estimate	14&15
26	Table giving descriptive information for each study included	12
27	Results of sensitivity testing (eg, subgroup analysis)	18
28	Indication of statistical uncertainty of findings	8&9

For peer review only

Item No	Recommendation	Reported on Page No
Reporting of discussion should include		
29	Quantitative assessment of bias (eg, publication bias)	eTable7
30	Justification for exclusion (eg, exclusion of non-English language citations)	6
31	Assessment of quality of included studies	eTable1
Reporting of conclusions should include		
32	Consideration of alternative explanations for observed results	10
33	Generalization of the conclusions (ie, appropriate for the data presented and within the domain of the literature review)	10
34	Guidelines for future research	11
35	Disclosure of funding source	3

From: Stroup DF, Berlin JA, Morton SC, et al, for the Meta-analysis Of Observational Studies in Epidemiology (MOOSE) Group. Meta-analysis of Observational Studies in Epidemiology. A Proposal for Reporting. *JAMA*. 2000;283(15):2008-2012. doi: 10.1001/jama.283.15.2008.

Transcribed from the original paper within the NEUROSURGERY® Editorial Office, Atlanta, GA, United Sates. August 2012.

For peer review only

For peer review only

For peer review only - http://bmjopen.bmj.com/site/about/guidelines.xhtml

BMJ Open

Smoking as a risk factor for lung cancer in women and men: a systematic review and meta-analysis

Journal:	*BMJ Open*
Manuscript ID	bmjopen-2018-021611.R2
Article Type:	Research
Date Submitted by the Author:	13-Jun-2018
Complete List of Authors:	O'Keeffe, Linda; MRC Integrative Epidemiology Unit, Bristol Medical School; Population Health Sciences, Bristol Medical School, University of Bristol<br>Taylor, Gemma; MRC Integrative Epidemiology Unit at the University of Bristol, Bristol Medical School, University of Bristol, Bristol, UK; UK Centre for Tobacco and Alcohol Studies, School of Experimental Psychology, University of Bristol, UK<br>Huxley, Rachel; School of Public Health, Faculty of Health Sciences, Curtin University,<br>Mitchell, Paul; Olivia Newton-John Cancer and Wellness Centre, Austin Health and Olivia Newton-John Cancer Research Institute, Heidelberg, VIC 3084, Australia<br>Woodward, Mark; The George Institute for Global Health,<br>Peters, Sanne; University of Oxford, George Institute for Global Health
**Primary Subject Heading**:	Epidemiology
Secondary Subject Heading:	Oncology, Public health
Keywords:	smoking, systematic review, lung cancer, sex-specific

SCHOLARONE™
Manuscripts

Smoking as a risk factor for lung cancer in women and men: a systematic review and meta-analysis

Linda M. O'Keeffe[1, 2*], Gemma Taylor[1, 2, 3*], Rachel R. Huxley[4], Paul Mitchell[5], Mark Woodward[6,7,8], Sanne A.E. Peters[6]

* Denotes equal contribution

1 MRC Integrative Epidemiology Unit at the University of Bristol, Bristol Medical School, University of Bristol, Bristol, UK
2 Population Health Sciences, Bristol Medical School, University of Bristol
3 UK Centre for Tobacco and Alcohol Studies, School of Experimental Psychology, University of Bristol, UK
4 School of Public Health, Faculty of Health Sciences, Curtin University, Perth, Australia
5 Olivia Newton-John Cancer and Wellness Centre, Austin Health and Olivia Newton-John Cancer Research Institute, Heidelberg, VIC 3084, Australia
6 The George Institute for Global Health, University of Oxford, Oxford, UK
7 The George Institute for Global Health, University of New South Wales, Sydney, Australia
8 Department of Epidemiology, Johns Hopkins University, Baltimore MD, USA

Correspondence: Dr. Linda O'Keeffe
MRC Integrative Epidemiology Unit at the University of Bristol, University of Bristol, Oakfield House, Oakfield Grove, BS8 2BN, United Kingdom T: +44 7534498919

Email address: lindaokeeffe1987@gmail.com

Funding: The MRC Integrative Epidemiology Unit at the University of Bristol is supported by the Medical Research Council and the University of Bristol [MC_UU_12013/6, MC_UU_12013/9]. LMOK is supported by a UK Medical Research Council Population Health Scientist fellowship (MR/M014509/1). GMJT is funded by a Cancer Research UK Postdoctoral Fellowship (C56067/A21330). SAEP is supported by a UK Medical Research Council Skills Development Fellowship (MR/P014550/1)

Data sharing statement: Technical appendix, statistical code, and dataset (of published data only) available from authors upon request.

Contributor statement: LMOK, GT, SAEP, RH and MW designed the study. LMOK and GT performed systematic searches, retrieved literature and performed data extraction. SAEP performed the analysis of the data. LMOK and GT wrote the first draft of the article. RH, MW and PM contributed important intellectual content and critical expertise and revisions to the manuscript.

Competing interest's statement: We have no competing interests to declare.

For peer review only - http://bmjopen.bmj.com/site/about/guidelines.xhtml

Abstract

Objectives: To investigate the sex-specific association between smoking and lung cancer.

Design: Systematic review and meta-analysis.

Data sources: We searched Pubmed and Embase, from January 1st, 1999 to April 15th, 2016 for cohort studies. Cohort studies before January 1st, 1999 were retrieved from a previous meta-analysis. Individual participant data from three sources was also available to supplement analyses of published literature.

Eligibility criteria for selecting studies: Cohort studies reporting the sex-specific relative risk (RR) of lung cancer associated with smoking.

Results: Data from 29 studies representing 99 cohort studies, seven million individuals and > 50,000 incident lung cancer cases were included. The sex-specific RRs and their ratio comparing women with men were pooled using random-effects meta-analysis with inverse-variance weighting. The pooled multiple-adjusted lung cancer RR was 6.99 (95% Confidence Interval (CI): 5.09, 9.59) in women and 7.33 (95% CI: 4.90, 10.96) in men. The pooled ratio of the RRs was 0.92 (95% CI: 0.72 – 1.16; I^2=89%; p<0.001), with no evidence of publication bias, or differences across major predefined participant and study subtypes. The women-to-men ratio of RRs were 0.99 (95% CI: 0.65, 1.52), 1.11 (95% CI: 0.75, 1.64), and 0.94 (95% CI: 0.69, 1.30), for light, moderate and heavy smoking respectively.

Conclusions: Smoking yields similar risks of lung cancer in women compared with men. However, these data may underestimate the true risks of lung cancer among women, as the smoking epidemic has not yet reached full maturity in women. Continued efforts to measure the sex-specific association of smoking with lung cancer are required.

For peer review only - http://bmjopen.bmj.com/site/about/guidelines.xhtml

Strengths and Limitations

- Evidence on the sex-specific association of smoking and lung cancer were meta-analysed in over seven million participants across 99 cohort studies.
- Several subgroup analyses were performed to examine the robustness of findings across different population subgroups.
- However, the smoking epidemic is not yet fully mature in women and risks of lung cancer in women may still be underestimated.
- Detailed data on smoking behaviour and data on specific subtypes of lung cancer were not available.

For peer review only

For peer review only - http://bmjopen.bmj.com/site/about/guidelines.xhtml

Introduction

Lung cancer is the leading cause of cancer death worldwide with 1.7 million global deaths attributed to cigarette smoking in 2015.[1] Tobacco use is the leading cause of lung cancer; 55% of lung cancer deaths in women and over 70% of lung cancer deaths in men are due to smoking.[1] These global estimates, however, mask major differences in smoking prevalence in men and women across populations, with rates below 5% for women in most Asian and African countries to 40% and above for men in many parts of Asia and Eastern Europe.[2] [2] In addition, smoking behaviour varies significantly by sex. For example, compared with women, men smoke more cigars and pipes,[3] take puffs of longer duration, and leave shorter butts,[4] which each could potentially predispose them to greater risks of smoking-related lung cancer. Substantial physiological differences between the sexes may also result in sex differences in the effects of smoking, particularly for women. For example, compared with men, women have a smaller lung size and different airway behaviour,[5] which may increase their susceptibility to lung cancer at lower levels of smoking. A recent meta-analysis showed that cigarette smoking confers a greater coronary hazard in women compared with men, which suggests the possibility that this may also be true for the risk of smoking-related lung cancer.[6]

A study of 50-year trends in smoking-related mortality in the US found that the relative risks of smoking-related lung cancer mortality were higher in men than women.[7] However, this sex difference was only apparent in the oldest cohorts with the longest follow-up, possibly reflecting greater cumulative tobacco exposure in men than in women. In contrast, a recent study in Korea, a population where smoking patterns continue to differ between the sexes, suggested that sex differences in the impact of smoking on lung cancer risk exist and differ by histologic subtype.[8] Analyses of a large UK primary care database showed that moderate and heavy smoking more strongly increase the risks of lung cancer in women than in men. [9]

Two recent meta-analyses examined the sex-specific association between smoking and lung cancer. In the most recent of these, males were found to have a greater risk of lung cancer associated with smoking compared with females.[10] However, virtually all data were from historical

For peer review only - http://bmjopen.bmj.com/site/about/guidelines.xhtml

case-control studies, which have several limitations, and the three included prospective studies provided contradictory results. While a previous meta-analysis by Lee et al [11] included 287 cohort and case-control studies and provided sex-specific estimates, single-sex cohorts were also included, sex differences in the smoking-related risk of lung cancer were not formally compared within studies, and only studies published up-to 1999 were included.

To resolve this uncertainty, we performed a systematic review and meta-analysis of prospective cohort studies published to date on the sex-specific association of smoking on the risk of fatal and non-fatal lung cancer. Our systematic review builds on these previous meta-analyses by adding literature from 1999 onwards and restricting the analyses to cohort studies, which are less prone to bias than case-control studies. In addition, we perform several pre-defined subgroup analyses which have not been performed in meta-analyses of cohort studies included in previous reviews and supplement our findings with results from three sources of individual participant data, not published previously. An important *a priori* consideration is the substantial sex difference in the maturity of the smoking epidemic with men being at a more advanced stage than women in most parts of the world.[2] This would be expected to translate into lower relative risk estimates for lung cancer in women than in men. Hence, the null hypothesis that smoking confers the same lung cancer hazard in both women and men, would be met if the ratio of the relative risks for lung cancer (women:men) was less than unity (reflecting a greater hazard in men than women). However, if the ratio of the relative risks was found to be unity (or higher) then this would suggest a greater hazard associated with tobacco exposure in women than in men.

For peer review only - http://bmjopen.bmj.com/site/about/guidelines.xhtml

Methods

Search strategy

This review was conducted using a predefined protocol and in accordance to the MOOSE guidelines (**eAppendix 1**). We systematically searched PubMed and EMBASE for studies published between January 1, 1999 and April 15th, 2016 that reported on the relationship between smoking and lung cancer in men and women from a general population. The computer-based searches combined MeSH and free-text terms related to "tobacco/smoking", "cancer", "sex" and "cohort studies". The full search criteria are available in **eAppendix 2**. Articles published before January 1st , 1999 were retrieved from a previous systematic review.[11] The reference lists of all relevant original research and review articles were scanned to capture missed studies. Two authors (LMOK and GMT) independently conducted the screening of studies and any disagreement was mediated by a third author (SAP).

Data extraction

Data were extracted, in duplicate, from studies deemed to meet the eligibility criteria. These included details on general study characteristics (study name, duration of follow-up, year of publication), information about the studied population (prevalence of smoking, mean age, number of men and women, incidence of lung cancer, whether lung cancer was fatal or non-fatal, and level of adjustment for covariates). We extracted sex-specific adjusted measures of relative risk and 95% confidence intervals.

Study selection

Observational cohort studies were included if they reported sex-specific relative risks (RRs), or equivalent, on the relationship between smoking and lung cancer. Studies were excluded if the variability around the point estimate was not reported, if they had not been adjusted for at least age, or if the study was performed in a population selected on the basis of prior lung cancer or

For peer review only - http://bmjopen.bmj.com/site/about/guidelines.xhtml
For peer review only

another major underlying chronic disease. In the case of duplicate reports from the same study, the report with the longest follow-up or the highest number of cases was included. Individual participant data (IPD) from studies available to the authors were also used; the Asia Pacific Cohort Studies Collaboration (APCSC), the National Health and Nutrition Examination Survey III (NHANES III), and the Scottish Heart Health Extended Cohort Study (SHHEC). The Newcastle-Ottawa Quality assessment (NOS) scale was used to assess the methodological quality of all included studies, on a 9-point scale (**eAppendix 3 & eTable 1**).[12]

Meta-analysis

The primary analysis was a comparison of the sex-specific RR of lung cancer (fatal or non-fatal) in current smokers versus non-smokers (defined either as former or never smokers). For each study, we obtained the natural log of the sex-specific RRs and calculated the differences. The differences were pooled across studies using random effects meta-analysis which allows the RR of lung cancer to vary from study to study, weighted by the inverse of the variances of the log RRs, and then back-transformed to obtain the pooled women-to-men ratio of the RRs (RRR). The standard error of the log RRR was calculated as the square root of the sum hetof the variance of the two sex-specific log RRs for each study. Pooled RRRs were computed separately for studies with only age-adjusted estimates and then for those studies with multiple-adjusted estimates. The set of multiple adjustments made was allowed to vary by study, but had to include at least one other risk factor in addition to age. The I^2 statistic was used to estimate the percentage of variability across studies due to between-study heterogeneity. The presence of publication bias was graphically examined using contour funnel plots, plotting the natural log of the RRR against its standard error, and tested using Begg's test. Pre-defined subgroup analyses were conducted to obtain the adjusted RRRs by study region (Asia or non-Asia and Asia, Europe, US, and Australia and New Zealand [ANZ]), year of study baseline (pre- or post-1985), study endpoint (fatal only or fatal and non-fatal combined), number of cigarettes smoked per day (>0 to 10, 10-20, >20), study quality ≤6 vs. >6 points) and follow-up time

For peer review only - http://bmjopen.bmj.com/site/about/guidelines.xhtml

(≤10 vs. >10 years). Random effects meta-analysis were used for all subgroup analyses and differences between subgroups were examined using meta-regression. To include the largest number of studies available, we combined the age-and multiple-adjusted estimates, taking the maximum available adjustment set available. In secondary analyses, we obtained the sex-specific RRs and RRRs comparing former smokers to never smokers and performed the same set of subgroup analyses. All analyses were performed using Stata version 12.0.

Patient and public involvement

There were no patients or applicable public involved in this review.

For peer review only

For peer review only - http://bmjopen.bmj.com/site/about/guidelines.xhtml

Results

Of the 9,519 unique records that were identified through the systematic search, 227 qualified for full-text evaluation (**Figure 1**). Of these, 25 separate studies provided information about sex differences in the association between smoking and lung cancer. This database was extended with individual participant data from APCSC (separately for Asia and ANZ), NHANES III, and SHHEC leading to a total of 29 individual estimates, representing a total of 99 cohort studies available for meta-analysis.

The characteristics of the included studies are described in **Table 1**. Overall, data were available from 99 cohorts, including 7,113,303 individuals (46% women) - not accounting for two cohorts that used Census data - and at least 51,161 incident cases of lung cancer (31% women). Forty-six cohorts were from Asia (61% of the individuals), six were from the USA (28%), 27 were from Europe (10%), and ten were from Australia and New Zealand (1%). Of 29 studies, four studies had a quality score of 5 out of 9, 9 studies had a score of 6, 12 studies had a score of 7 and 4 studies with a score of 8 (**eTable 1**).

Eighteen studies reported on the prevalence of smoking, which varied widely by study, region, and sex. The prevalence of smoking ranged from 1% to 47% in women and from 1% to 70% in men. In all but two studies, the prevalence of smoking was higher in men than women, especially in Asia where typically less than 10% of women were smokers compared with over 50% of men. Smoking cessation rates were also higher among men (7-61%) than women (<1-39%).

Risk of lung cancer in current smokers versus non-smokers

Compared with non-smoking, current smoking was associated with an age-adjusted RR of lung cancer of 7.48 (95% Confidence Interval (CI): 5.29, 10.60) in women and 8.78 (95% CI: 6.13, 12.57) in men (**Table 2 & eFigure 1**). The pooled age-adjusted women-to-men RRR was 0.81 (95% CI: 0.62, 1.04), with substantial between-study heterogeneity (I^2=86%; P<0.001) (**Table 2 & eFigure 2**). The

For peer review only - http://bmjopen.bmj.com/site/about/guidelines.xhtml

multiple-adjusted RR of lung cancer associated with current smoking was 6.99 (95% CI: 5.09, 9.59) in women and 7.33 (95% CI: 4.90, 10.96) in men (**Table 2 & Figure 2**). The corresponding RRR was 0.92 (95% CI: 0.72, 1.16) and between-study heterogeneity was substantial (I^2=89%; P<0.001) (**Table 2 & Figure 3**). There was no evidence of publication bias based on the Begg's test (P=0.75) (**eFigure 3**).

The sex difference in the risk of smoking-related lung cancer in our main analysis did not differ in subgroup analyses stratified by the women-to-men ratio of current smokers (P=0.90), women-to-men ratio of lung cancer incidence in the studies (P=0.64), year of study baseline (P = 0.66), study endpoint (P=0.21) or study region (P=0.73) (**Table 3**). The sex difference in the risk of smoking-related lung cancer in our main analysis also did not differ by or follow-up time (P=0.83) or study quality (P=0.69). The RRR was 0.93 (95% CI: 0.72, 1.20) for studies from Asia and 0.87 (95% CI: 0.66, 1.14) for studies from USA, Europe, or Australia and New Zealand.

The risk of smoking-related lung cancer increased according to the number of cigarettes smoked per day in both sexes (**Table 2**). In women, the RRs were 5.30 (95% CI: 3.52, 7.97), 10.67 (95% CI: 7.43, 15.33), and 17.09 (95% CI: 12.11, 24.11) across subgroups of <10, 10 to 20, and >20 cigarettes per day versus non-smoking, respectively. Corresponding RRs in men were 4.97 (95% CI: 2.74, 9.03), 8.93 (95% CI: 4.90, 16.28), and 14.61 (95% CI: 8.33, 25.59), respectively. The RRRs in these subgroups were 0.99 (95% CI: 0.65, 1.52), 1.11 (95% CI: 0.75, 1.64), and 0.94 (95% CI: 0.69, 1.30), respectively.

Risk of lung cancer in former smokers versus never smokers

Data from 89 cohorts, including 6,006,725 individuals and 38,244 cases of lung cancer, reported on the risk of lung cancer in former smokers compared with never smokers. The age-adjusted RR of lung cancer associated with former smoking was 2.82 (95% CI: 2.25, 3.54) in women and 3.01 (95% CI: 2.23, 4.08) in men (**Table 2** & **eFigure 4**); the age-adjusted RRR was 0.88 (95% CI: 0.69, 1.14) (I^2=64%; P<0.001) (**Table 2** & **eFigure 5**). The corresponding multiple-adjusted RRs were 3.14 (95% CI:

For peer review only - http://bmjopen.bmj.com/site/about/guidelines.xhtml

2.45, 4.03) in women and 3.13 (95% CI: 2.06, 4.76) in men (**Table 2** & **eFigure6**). There was no statistical evidence that the effects of smoking cessation on risk of lung cancer differed between the sexes; the multiple-adjusted RRR was 0.89 (95% CI: 0.69, 1.13) (I^2=69%; P<0.001) (**Table 2** & **eFigure 7**). There was no evidence that the RRR differed across various subgroup analyses (**Table 3**).

For peer review only

For peer review only - http://bmjopen.bmj.com/site/about/guidelines.xhtml

Discussion

In this systematic review and meta-analysis, comprising data from more than seven million participants, 99 cohort studies and over 50,000 incident cases of lung cancer, there was no evidence for a difference in the risk of smoking-related lung cancer in women compared with men. This was true across a range of subgroup and sensitivity analyses. However, as smoking prevalence and intensity was higher in men compared with women in most studies included in this analysis, there may yet be an unrealised sex-difference in the risk of smoking-related lung cancer that will only become fully manifest as the smoking epidemic reaches full maturity in women.[2]

The seven-fold higher relative risks of lung cancer associated with smoking found in the present meta-analysis are considerably smaller than the 20-fold increased risks reported in the Million Women's Study (MWS)[13] and the British Doctors Study.[14] Both these studies had the advantage of capturing smoking-related risks in populations that had smoked for long enough for the effects to become fully manifest, highlighting the importance of taking into consideration the stage of the tobacco epidemic in each sex. The lack of any appreciable sex difference in the relative risks of lung cancer is surprising given men's greater cumulative exposure to smoking, in most populations, compared with women. In addition, men have a greater exposure to other risk factors for lung cancer including occupational carcinogens. [15] Men also smoke more cigars and pipes, [3] take longer puffs of longer duration and leave shorter butts compared with women.[4] Hence, it may be reasonable to surmise that the relative risk estimates of smoking-related lung cancer in women may eventually exceed those of men, once cumulative exposure to smoking in women is comparable to that in men. In a previous meta-analysis, using similar methodology, we found that smoking conferred a 25% greater relative risk of coronary heart disease (CHD) in women than in men. Two possible explanations for why a similar pattern is not observed for lung cancer are that, first, the lag-time of smoking associated with CHD is considerably shorter than for lung cancer[16], and second the pathways by which smoking increases risk are different between CHD and lung cancer.

For peer review only - http://bmjopen.bmj.com/site/about/guidelines.xhtml

Although not assessed in this analysis, evidence suggests that there are sex differences in the pattern of lung cancer among never smokers, with a higher prevalence of lung cancer among never-smoking women than never-smoking men.[17 18] A US study amongst 500,000 people found a 30% higher incidence of lung cancer in women never smokers compared with men never smokers.[19] An Australian study found the proportion of lung cancer patients who had never smoked was approximately 18% in women and 3% of men.[20] The reasons for this sex difference are not clear, but women may have increased exposure to environmental tobacco smoke[21] or other environmental carcinogens such as indoor air pollution[22], or sex-related differences in the metabolism of environmental carcinogens. The possibility of greater exposure to environmental tobacco smoke and other environmental carcinogens in women compared with men could have resulted in a greater underestimation of the association between smoking and lung cancer in women than men. This, in turn, could have impacted the sex difference in risk of smoking-related lung cancer reported in this study.

Our study has several strengths including restriction to cohort studies which provide more robust evidence of the associations compared with case-control studies. Differences between case-control and cohort studies may also explain why a previous meta-analysis of case-control studies (which included only 3 cohort studies) showed higher relative risk of lung cancer in men compared with women.[10] Other strengths to our study include an update of findings to include studies published after 1999,[11] with supplementation of published literature by individual participant data from three established population databases. We have also performed a range of pre-specified sensitivity analyses and several subgroup analyses which were not performed in previous meta-analyses. Our results were consistent across regions and irrespective of the women-to-men smoking ratio, suggesting that underestimation of the association of smoking with lung cancer in women due to sex differences in smoking prevalence and underreporting of smoking is unlikely. This is especially

For peer review only - http://bmjopen.bmj.com/site/about/guidelines.xhtml

relevant for parts of Asia where the prevalence of smoking in women is typically <10% and where smoking among women remains relatively socially unacceptable. As the up-take of smoking continues among women in countries where significant sex differences in smoking prevalence exist, the sex-specific risks of lung cancer due to smoking may become further apparent. This is also true in regards to Western countries where differences in prevalence between women and men have reduced substantially over time, with prevalence of smoking in younger cohorts of women and men approaching unity.[23] The limitations of this study include heterogeneity across studies in study design, study population, verification of smoking status, and outcome ascertainment. Assessment of smoking status differed across studies and was generally self-reported, which may have introduced measurement error.[24] Notably, compared with men, women typically are more likely to underreport smoking status, and underreporting is especially prevalent in countries where female smoking is not culturally acceptable.[25] The lack of standardisation across studies in how smoking status was obtained, including how smoking dose and duration were measured is also a major limitation. In addition, there was insufficient data available to examine whether there were sex differences in the impact of age at smoking initiation and smoking duration on the risk of lung cancer. The reference group of non-smokers in our analysis of current smoking was composed of former and never smokers which may inflate the risks of smoking-related lung cancer risk among non-smokers. However, we have also examined former smoking compared with never smoking and demonstrated no appreciable sex differences in the risks of smoking-related lung cancer in this group, which provides some evidence that the inclusion of former smokers in the reference category is unlikely to have biased the sex difference in our main analysis. We quantified sex differences in the risk of lung cancer associated with smoking based on RRs rather than absolute risks. This might introduce a statistical artefact, in which the generally higher absolute risk for lung cancer in men, and the same risk difference subsequent to smoking in each sex, would translate to a greater relative risk in women than men. However, our previous meta-analyses on risk factors for cardiovascular diseases demonstrated that sex differences in RRs are not inevitable[26], despite differences in absolute risks.

For peer review only - http://bmjopen.bmj.com/site/about/guidelines.xhtml

Compared with absolute risks, RRs are more stable across populations with different background risks, which makes them suitable for meta-analyses. In addition, RRs are reported much more commonly than absolute risks. In our review, no studies reported adjusted absolute risks, with standard errors, that allow for meta-analyses. We therefore believe that usage of RRs in the present analysis is appropriate. In addition, while we have aimed to assess study quality using the widely accepted and used Newcastle-Ottawa Scale, the value and contribution of quality assessment scales such as this to systematic reviews and meta-analyses continues to be debated. [27-29] Finally, there are differences between men and women in histological subtypes of lung cancer. Adenocarcinoma is more common in women and squamous cell carcinoma is more common in men. [30] Smoking is more strongly associated with squamous cell carcinoma than adenocarcinoma. [30] Few studies reported the sex-specific association of smoking with histological subtypes of cancer, which precluded the examination of sex differences in the association of smoking-related lung cancer subtypes and this remains an important limitation of our review. [30] Further studies of the smoking-related risks of lung cancer in women and men are required as the smoking epidemic reaches its full maturity in women. Given the later up-take of smoking in women, studies which allow sufficient lag time for lung cancer to develop are essential. In addition, reducing under-reporting of smoking in women, using standardised and robust methods for the ascertainment of smoking status and smoking behaviours and more extensive measurement and adjustment for confounders which differ by sex (such as exposure to environmental tobacco smoke) is also important for future work, as well as examination of histological subtypes of lung cancer which was not possible in this review.

In conclusion, this meta-analysis, summarising all available literature to date, shows that the effect of smoking on risk of lung cancer is similar in women and men. However, these data may yet underestimate the true relative risk of smoking in women, given later uptake and lower intensity of smoking in women. Although strides have been made in reducing smoking rates particularly in high-income countries, continuing efforts to measure the effects of smoking on disease outcomes are

For peer review only - http://bmjopen.bmj.com/site/about/guidelines.xhtml

required, as the smoking epidemic has not yet reached its global peak, particularly among women. In addition, tobacco control programmes that dissuade smokers of both sexes from smoking but which also encourage individuals to quit remain a priority.

For peer review only

For peer review only - http://bmjopen.bmj.com/site/about/guidelines.xhtml

Figure 1: Flow chart of study selection

Figure 2: Multiple-adjusted relative risk for incident lung cancer in women and men, comparing current smokers to non-smokers

Legend: APCSC, Asia Pacific Cohort Studies Collaboration; ARIC, Atherosclerosis Risk in Communities study; CPS, Cancer Prevention Study; EHS, Elderly Health Services; EPIC, European Prospective Investigation into Cancer; JACC, Japan Collaborative Cohort Study; JPHC, Japan Public Health Centre study; NHANES III, National Health And Nutrition Examination Survey III; NHIS, National Health Interview Survey; NIH-AARP, NIH-AARP Diet and Health Study ; SHHEC, Scottish Heart Health Extended Cohort Study; TPCS, Three-Prefecture Cohort Study. Multiple-adjusted includes anything that adjusted for more than just age. These covariates are listed in Table 1.Figures may contain less than 29 studies because we report age- and multiple-adjusted results separately. Some studies only contributed age-adjusted results whereas others only provided multiple-adjusted results. However, the count of unique studies that contributed to at least one of these analyses is 29.

Figure 3: Multiple-adjusted women-to-men ratio of relative risks for incident lung cancer, comparing current smokers to non-smokers

Legend: APCSC, Asia Pacific Cohort Studies Collaboration; ARIC, Atherosclerosis Risk in Communities study; CPS, Cancer Prevention Study; EHS, Elderly Health Services; EPIC, European Prospective Investigation into Cancer; JACC, Japan Collaborative Cohort Study; JPHC, Japan Public Health Centre study; NHANES III, National Health And Nutrition Examination Survey III; NHIS, National Health Interview Survey; NIH-AARP, NIH-AARP Diet and Health Study ; SHHEC, Scottish Heart Health Extended Cohort Study; TPCS, Three-Prefecture Cohort Study. Multiple-adjusted includes anything that adjusted for more than just age. These covariates are listed in Table 1.Figures may contain less than 29 studies because we report age- and multiple-adjusted results separately. Some studies only contributed age-adjusted results whereas others only provided multiple-adjusted results. However, the count of unique studies that contributed to at least one of these analyses is 29.

For peer review only

For peer review only - http://bmjopen.bmj.com/site/about/guidelines.xhtml

Table 1: Characteristics of included studies

Study name *	Location	Age range, (y)	Baseline year	Follow-up, (years)	NOS score (points)	N (%) women	N (%) lung cancer in women	Fatal/ non-fatal	Current smoker, % W/M		Former smoker, % W/M		Maximum available adjustment
APCSC – ANZ[31] (8 cohorts)	ANZ	20-107	1961-93	10	6	87,130 (52)	501 (28)	F	7	11	11	18	Age, education, BMI
APCSC - Asia[31] (33 cohorts)	Asia	20-104	1989-96	7	6	476,755 (35)	1,275 (19)	F	2	59	<1	7	Age, education, BMI
ARIC[32] (1 cohort)	USA	45-64	1987-89	20	8	14,610 (54)	470 (37)	F/NF	25	44	22	28	Age, race
China Kadoorie Biobank[33 34] (1 cohort)	China	35-74	2004-08	7	7	512,891 (59)	1,953 (37)	F	3	61	1	15	Age, area, education, alcohol
China National Hypertension Survey[35] (1 cohort)	China	15+	1991	8	7	155,131 (51)	NR	F	14	63	NR	NR	Age, education, region, HT, overweight/obesity, alcohol, PA, DM
Copenhagen Cohort Studies[36] (3 cohorts)	Denmark	20+	1964-92	14	6	30,874 (44)	867 (23)	F/NF	NR	NR	NR	NR	Age
CPS I (1 cohort)	USA	55+	1959	6	5	518,982 (65)	1,293 (21)	F	15	40	4	17	Age, education, race
CPS II[37] (1 cohort)	USA	55+	1982	6	5	746,485 (61)	4,957 (36)	F	18	24	21	43	Age, education, race
EHS[38 39] (1 cohort)	China	65+	1998-00	11	7	65,510 (66)	1,096 (27)	F	4	10	8	20	Age, education, alcohol, depression, health status, social security assistance, housing type, expenditure
EPIC[40] (23 cohorts)	Europe	30-70	1992-00	11	8	441,211 (70)	2,995 (49)	F/NF	21	27	25	38	Age, education, BMI, alcohol, PA, energy intake, diet
JPHC[41], JACC, TPCS[37 42 43] (3 cohorts)	Japan	40-79	1983-94	10	6	296,836 (53)	NR	F	8	54	2	25	Age
Korean Cancer Prevention Study[44] (1 cohort)	Korea	30-95	1993-95	9	6	1,212,906 (32)	4,238 (14)	F	5	57	3	23	Age
Korean National Health Insurance Service[8] (1 cohort)	Korea	20+	1998-99	10	7	1,355,891 (31)	6,491 (15)	F/NF	1	56	1	13	Age, BMI, alcohol, PA
Malmo Preventive Project[45] (1 cohort)	Sweden	27-61	1974-92	24	5	33,346 (33)	436 (21)	F	36	49	19	27	Age, FEV, SES, marital status
Migrant Study[46] (1 cohort)	Norway	33-72	1964-65	21	7	26,126 (55)	435 (23)	F	26	46	8	28	Age
New Zealand Census 1981[47] (1 cohort)	New Zealand	25+	1981	5	7	NR	4,188 (28)	F/NF	NR	NR	NR	NR	Age, race
New Zealand Census 1996[47] (1 cohort)	New Zealand	25+	1996	5	7	NR	4,467 (44)	F/NF	NR	NR	NR	NR	Age, race
NHANES III (1 cohort)	USA	18-90	1988-94	13	7	20,006 (53)	320 (36)	F	21	30	17	32	Age, education, BMI
NHIS[48] (1 cohort)	USA	25+	1997-04	7	7	202,248 (56)	1,223 (44)	F	21	26	20	29	Age, education, BMI, alcohol
NIH-AARP[7 49] (1 cohort)	USA	50-71	1995-96	11	7	452,131 (41)	9,381 (37)	F/NF	17	13	39	61	Age, education, alcohol, ethnicity
Norwegian Counties Study[50-52] (1 cohort)	Norway	20-49	1974-78	23	6	48,682 (48)	686 (35)	F	43	50	11	18	Age, SBP, TC, TG, PA, BMI, height, sickness leave, disability pension, family history of CHD
Renfrew/Paisley Study[53] (1 cohort)	Scotland	45-64	1972-76	20	6	15,393 (54)	NR	F	47	59	7	25	Age
Reykjavik Study[54] (1 cohort)	Iceland	32-60	1967	27	7	22,946 (50)	472 (42)	F/NF	39	30	15	24	Age
Shanghai Health Study[55] (2 cohorts)	China	40-74	1997-06	10	7	134,335 (54)	839 (49)	F	3	70	NR	NR	Age, education, BMI, alcohol, PA, income

For peer review only - http://bmjopen.bmj.com/site/about/guidelines.xhtml

**SHHEC (1 cohort)**	Scotland	25-75	1984-87	23	6	17,731 (51)	558 (38)	F	40	51	21	27	Age, education, BMI
**Singapore Chinese Health Study[56] (1 cohort)**	Singapore	45-74	1993-98	12	8	61,320 (55)	905 (31)	F/NF	6	36	3	21	Age, dialect, year of recruitment, education, alcohol, PA
**Swedish Smoking Habit Survey[57 58] (1 cohort)**	Sweden	18-69	1963	33	8	41,544 (60)	442 (45)	F	18	27	5	23	Age, area
**Swiss National Cohort [59] (4 cohorts)**	Switzerland	14-99	1977-93	19	5	35,703 (53)	426 (29)	F	27	43	13	24	Age, survey, education, alcohol, PA, civil status, nationality, diet
**Wen[60] (2 cohorts)**	Taiwan	35+	1982-92	20	5	86,580 (39)	247 (2)	F	1	41	<1	11	Age

APCSC, Asia Pacific Cohort Studies Collaboration; ANZ, Australia and New-Zealand, ARIC, Atherosclerosis Risk in Communities study; BMI, body mass index; CPS, Cancer Prevention Study; CHD, coronary heart disease; DM, diabetes mellitus; EHS, Elderly Health Services; EPIC, European Prospective Investigation into Cancer; F, fatal; FEV, forced expiratory volume, JACC, Japan Collaborative Cohort study; JPHC, Japan Public Health Centre study; NF, non-fatal; NHANES III, National Health And Nutrition Examination Survey III; NHIS, National Health Interview Survey ; NIH-AARP, NIH-AARP Diet and Health Study ; NOS, Newcastle Ottawa Scale; NR, not reported; PA, physical activity; SES, socioeconomic status; SHHEC, Scottish Heart Health Extended Cohort Study; SBP, systolic blood pressure; TC, total cholesterol; TG, triglycerides; TPCS, Three-Prefecture Cohort Study

*Note that where several studies are cited for a single cohort, data may be extracted from multiple studies if all data required is not available in the most up-to-date and relevant study.

.

For peer review only

For peer review only - http://bmjopen.bmj.com/site/about/guidelines.xhtml

Table 2: Sex-specific pooled relative risks and ratio of relative risks for lung cancer associated with smoking

		RR in women	RR in men	Ratio of RR
Age-adjusted	Former vs. never	2.82 (2.25, 3.54)	3.01 (2.23, 4.08)	0.88 (0.69, 1.14)
	Current vs. not	7.48 (5.29. 10.60)	8.78 (6.13, 12.57)	0.81 (0.62, 1.04)
Multiple-adjusted	Former vs. never	3.14 (2.45, 4.03)	3.13 (2.06, 4.76)	0.89 (0.69, 1.13)
	Current vs. not	6.99 (5.09, 9.59)	7.33 (4.90, 10.96)	0.92 (0.72, 1.16)
Maximum-adjusted	Former vs. never	2.92 (2.35, 3.63)	3.08 (2.31, 4.11)	0.86 (0.71, 1.05)
	Current vs. not	7.32 (5.58, 9.61)	8.05 (5.90, 10.98)	0.89 (0.73, 1.08)
**Cigarettes per day among current smokers vs. never (maximum available-adjusted)**				
	10 or less	5.30 (3.52, 7.97)	4.97 (2.74, 9.03)	0.99 (0.65, 1.52)
	10 to 19	10.67 (7.43, 15.33)	8.93 (4.90, 16.28)	1.11 (0.75, 1.64)
	20 or more	17.09 (12.11, 24.11)	14.61 (8.33, 25.59)	0.94 (0.69, 1.30)

Multiple-adjusted includes anything that adjusted for more than just age. Maximum available adjustment refers to the most adjustments provided in the study. For some studies, this would have been age-adjusted whereas other studies adjusted for more factors than age only (i.e. multiple-adjusted). These covariates are listed in Table 1.

For peer review only

For peer review only - http://bmjopen.bmj.com/site/about/guidelines.xhtml

Table 3: Maximally adjusted sex difference in the association of smoking with lung cancer, in subgroup analyses

	N studies	Former vs. never	P for interaction*	N studies	Current vs. not	P for interaction*
**Study region**						
Asia	40	1.07 (0.83, 1.37)		46	0.93 (0.72, 1.20)	
Non-Asia	49	0.73 (0.58, 0.93)	0.06	53	0.87 (0.66, 1.14)	0.73
**Study region**						
Asia	40	1.07 (0.83, 1.37)		46	0.93 (0.72, 1.20)	
USA	5	0.60 (0.42, 0.84)		6	0.58 (0.37, 0.91)	
Europe	36	0.81 (0.60, 1.10)		37	0.99 (0.63, 1.57)	
ANZ	8	1.41 (0.65, 3.04)	0.55	10	1.11 (0.97, 1.28)	0.69
**Year of study baseline**						
1985 or before	23	0.79 (0.56, 1.12)		25	0.96 (0.66, 1.40)	
After 1985	66	0.92 (0.73, 1.16)	0.43	74	0.85 (0.68, 1.06)	0.66
**Women to men smoking prevalence**						
>67% lower in women	39	1.16 (0.83, 1.62)		43	0.96 (0.72, 1.28)	
33-67% lower in women	19	0.72 (0.51, 1.03)		20	0.75 (0.50, 1.14)	
0-33% lower in women	28	0.78 (0.59, 1.03)	0.26	29	0.97 (0.59, 1.58)	0.90
**Women to men lung cancer rate**						
>= 50% lower in women	80	0.85 (0.65, 1.10)		83	0.85 (0.62, 1.17)	
0- 50% lower in women	6	0.83 (0.61, 1.13)	0.79	9	0.94 (0.68, 1.31)	0.64
**Study endpoint**						
Fatal lung cancer only	57	0.94 (0.68, 1.29)		67	0.97 (0.77, 1.21)	
Fatal and non-fatal lung cancer	32	0.80 (0.64, 1.01)	0.57	32	0.72 (0.48, 1.06)	0.21
**Duration of follow-up**						
≤ 10 yrs	48	0.90 (0.60, 1.35)		53	0.91(0.68, 1.24)	
>10 yrs	41	0.85 (0.71, 1.05)	0.92	46	0.87 (0.67, 1.12)	0.83
**Study quality**						
≤ 6 points	58	0.85 (0.64, 1.13)		61	0.84 (0.53, 1.12)	
>6 points	31	0.89 (0.66, 1.20)	0.81	38	0.93 (0.72, 1.20)	0.69

Random effects meta-analysis were used for all subgroup analyses and differences between subgroups were examined using meta-regression. *P for interaction assessed using meta-regression.

For peer review only - http://bmjopen.bmj.com/site/about/guidelines.xhtml

References

1. Institute for Health Metrics and Evaluation. Global Burden of Disease 2015 2015 [Available from: http://vizhub.healthdata.org/gbd-compare/#
2. Reitsma MB, Fullman N, Ng M, et al. Smoking prevalence and attributable disease burden in 195 countries and territories, 1990–2015: a systematic analysis from the Global Burden of Disease Study 2015. *The Lancet* 2017
3. Nelson DE, Davis RM, Chrismon JH, et al. Pipe smoking in the United States, 1965–1991: prevalence and attributable mortality. *Preventive medicine* 1996;25(2):91-99.
4. Melikian AA, Djordjevic MV, Hosey J, et al. Gender differences relative to smoking behavior and emissions of toxins from mainstream cigarette smoke. *Nicotine & Tobacco Research* 2007;9(3):377-87.
5. Becklake MR, Kauffmann F. Gender differences in airway behaviour over the human life span. *Thorax* 1999;54(12):1119-38.
6. Huxley RR, Woodward M. Cigarette smoking as a risk factor for coronary heart disease in women compared with men: a systematic review and meta-analysis of prospective cohort studies. *The Lancet* 2011;378(9799):1297-305.
7. Thun MJ, Carter BD, Feskanich D, et al. 50-year trends in smoking-related mortality in the United States. *New England Journal of Medicine* 2013;368(4):351-64.
8. Yun YD, Back JH, Ghang H, et al. Hazard ratio of smoking on lung cancer in Korea according to histological type and gender. *Lung* 2016;194(2):281-89.
9. Powell HA, Iyen-Omofoman B, Hubbard RB, et al. The association between smoking quantity and lung cancer in men and women. *CHEST Journal* 2013;143(1):123-29.
10. Yu Y, Liu H, Zheng S, et al. Gender susceptibility for cigarette smoking-attributable lung cancer: A systematic review and meta-analysis. *Lung cancer* 2014;85(3):351-60.
11. Lee PN, Forey BA, Coombs KJ. Systematic review with meta-analysis of the epidemiological evidence in the 1900s relating smoking to lung cancer. *BMC cancer* 2012;12(1):385.
12. G W, B S, D OC, et al. The Newcastle-Ottawa Scale (NOS) for Assessing the Quality of Nonrandomised Studies in Meta-analyses Ottawa Ottawa Hospital Research Institute; 2012 [
13. Pirie K, Peto R, Reeves GK, et al. The 21st century hazards of smoking and benefits of stopping: a prospective study of one million women in the UK. *The Lancet* 2013;381(9861):133-41.
14. Doll R, Peto R, Boreham J, et al. Mortality in relation to smoking: 50 years' observations on male British doctors. *BMJ (Clinical research ed)* 2004;328(7455):1519.
15. Olsson AC, Gustavsson P, Zaridze D, et al. Lung cancer risk attributable to occupational exposures in a multicenter case-control study in Central and Eastern Europe. *Journal of occupational and environmental medicine* 2011;53(11):1262-67.
16. Ockene JK, Kuller LH, Svendsen KH, et al. The relationship of smoking cessation to coronary heart disease and lung cancer in the Multiple Risk Factor Intervention Trial (MRFIT). *American journal of public health* 1990;80(8):954-58.
17. Zang EA, Wynder EL. Differences in lung cancer risk between men and women: examination of the evidence. *Journal of the National Cancer Institute* 1996;88(3-4):183-92.
18. Thun MJ, Hannan LM, Adams-Campbell LL, et al. Lung cancer occurrence in never-smokers: an analysis of 13 cohorts and 22 cancer registry studies. *PLoS medicine* 2008;5(9):e185.
19. Freedman ND, Leitzmann MF, Hollenbeck AR, et al. Cigarette smoking and subsequent risk of lung cancer in men and women: analysis of a prospective cohort study. *The lancet oncology* 2008;9(7):649-56.
20. Mitchell P, Thursfield VJ, Ball DL, et al. Lung cancer in Victoria: are we making progress. *The Medical journal of Australia* 2013;199(10):674-9.
21. Sisti J, Boffetta P. What proportion of lung cancer in never-smokers can be attributed to known risk factors? *International journal of cancer* 2012;131(2):265-75.

For peer review only - http://bmjopen.bmj.com/site/about/guidelines.xhtml

22. Hosgood III HD, Boffetta P, Greenland S, et al. In-home coal and wood use and lung cancer risk: a pooled analysis of the International Lung Cancer Consortium. *Environmental health perspectives* 2010;118(12):1743.
23. Peters SA, Huxley RR, Woodward M. Do smoking habits differ between women and men in contemporary Western populations? Evidence from half a million people in the UK Biobank study. *BMJ open* 2014;4(12):e005663.
24. Patrick DL, Cheadle A, Thompson DC, et al. The validity of self-reported smoking: a review and meta-analysis. *American journal of public health* 1994;84(7):1086-93.
25. Jung-Choi K-H, Khang Y-H, Cho H-J. Hidden female smokers in Asia: a comparison of self-reported with cotinine-verified smoking prevalence rates in representative national data from an Asian population. *Tobacco Control* 2012;21(6):536-42.
26. Huxley RR, Peters SA, Mishra GD, et al. Risk of all-cause mortality and vascular events in women versus men with type 1 diabetes: a systematic review and meta-analysis. *The Lancet Diabetes & Endocrinology* 2015;3(3):198-206.
27. Katikireddi SV, Egan M, Petticrew M. How do systematic reviews incorporate risk of bias assessments into the synthesis of evidence? A methodological study. *Journal of epidemiology and community health* 2014:jech-2014-204711.
28. Viswanathan M, Berkman ND, Dryden DM, et al. Assessing risk of bias and confounding in observational studies of interventions or exposures: further development of the RTI item bank. 2013
29. Sanderson S, Tatt ID, Higgins J. Tools for assessing quality and susceptibility to bias in observational studies in epidemiology: a systematic review and annotated bibliography. *International journal of epidemiology* 2007;36(3):666-76.
30. Devesa SS, Bray F, Vizcaino AP, et al. International lung cancer trends by histologic type: male: female differences diminishing and adenocarcinoma rates rising. *International journal of cancer* 2005;117(2):294-99.
31. Huxley R, Jamrozik K, Lam TH, et al. Impact of smoking and smoking cessation on lung cancer mortality in the Asia-Pacific region. *American journal of epidemiology* 2007;165(11):1280-86.
32. Prizment AE, Yatsuya H, Lutsey PL, et al. Smoking behavior and lung cancer in a biracial cohort: The atherosclerosis risk in communities study. *American journal of preventive medicine* 2014;46(6):624-32.
33. Chen Z, Peto R, Zhou M, et al. Contrasting male and female trends in tobacco-attributed mortality in China: evidence from successive nationwide prospective cohort studies. *The Lancet* 2015;386(10002):1447-56.
34. Chen ZM, Peto R, Iona A, et al. Emerging tobacco-related cancer risks in China: A nationwide, prospective study of 0.5 million adults. *Cancer* 2015;121(S17):3097-106.
35. Gu D, Kelly TN, Wu X, et al. Mortality attributable to smoking in China. *New England Journal of Medicine* 2009;360(2):150-59.
36. Prescott E, Osier M, Rein HO, et al. Gender and smoking-related risk of lung cancer. *Epidemiology (Cambridge, Mass)* 1998;9(1):79-83.
37. Marugame T, Sobue T, Satoh H, et al. Lung cancer death rates by smoking status: Comparison of the Three-Prefecture Cohort study in Japan to the Cancer Prevention Study II in the USA. *Cancer science* 2005;96(2):120-26.
38. Lam TH, Li ZB, Ho SY, et al. Smoking, quitting and mortality in an elderly cohort of 56 000 Hong Kong Chinese. *Tobacco control* 2007;16(3):182-89.
39. Lam TH, Xu L, Schooling CM, et al. Smoking and mortality in a prospective cohort study of elderly Chinese in Hong Kong. *Addiction* 2015;110(3):502-10.
40. Agudo A, Bonet C, Travier N, et al. Impact of cigarette smoking on cancer risk in the European prospective investigation into cancer and nutrition study. *Journal of Clinical Oncology* 2012;30(36):4550-57.

For peer review only - http://bmjopen.bmj.com/site/about/guidelines.xhtml

41. Sobue T, Yamamoto S, Hara M, et al. Cigarette smoking and subsequent risk of lung cancer by histologic type in middle-aged Japanese men and women: The JPHC study. *International journal of cancer* 2002;99(2):245-51.
42. Akiba S, Hirayama T. Cigarette smoking and cancer mortality risk in Japanese men and women--results from reanalysis of the six-prefecture cohort study data. *Environmental health perspectives* 1990;87:19.
43. Katanoda K, Marugame T, Saika K, et al. Population attributable fraction of mortality associated with tobacco smoking in Japan: a pooled analysis of three large-scale cohort studies. *Journal of epidemiology* 2008;18(6):251-64.
44. Jee SH, Samet JM, Ohrr H, et al. Smoking and cancer risk in Korean men and women. *Cancer Causes & Control* 2004;15(4):341-48.
45. Ekberg-Aronsson M, Nilsson PM, Nilsson J-Å, et al. Mortality risks among heavy-smokers with special reference to women: a long-term follow-up of an urban population. *European journal of epidemiology* 2007;22(5):301-09.
46. Engeland A, Haldorsen T, Andersen A, et al. The impact of smoking habits on lung cancer risk: 28 years' observation of 26,000 Norwegian men and women. *Cancer Causes and Control* 1996;7(3):366-76.
47. Blakely T, Barendregt JJ, Foster RH, et al. The association of active smoking with multiple cancers: national census-cancer registry cohorts with quantitative bias analysis. *Cancer Causes & Control* 2013;24(6):1243-55.
48. Jha P, Ramasundarahettige C, Landsman V, et al. 21st-century hazards of smoking and benefits of cessation in the United States. *New England Journal of Medicine* 2013;368(4):341-50.
49. Freedman ND, Abnet CC, Caporaso NE, et al. Impact of changing US cigarette smoking patterns on incident cancer: risks of 20 smoking-related cancers among the women and men of the NIH-AARP cohort. *International journal of epidemiology* 2016;45(3):846-56.
50. Bjartveit K, Tverdal A. Health consequences of sustained smoking cessation. *Tobacco control* 2009;18(3):197-205.
51. Bjartveit K, Tverdal A. Health consequences of smoking 1–4 cigarettes per day. *Tobacco control* 2005;14(5):315-20.
52. Vollset SE, Tverdal A, Gjessing HkK. Smoking and deaths between 40 and 70 years of age in women and men. *Annals of internal medicine* 2006;144(6):381-89.
53. Marang-van de Mheen PJ, Smith GD, Hart CL, et al. Are women more sensitive to smoking than men? Findings from the Renfrew and Paisley study. *International journal of epidemiology* 2001;30(4):787-92.
54. Tulinius H, Sigfusson N, Sigvaldason H, et al. Risk factors for malignant diseases: a cohort study on a population of 22,946 Icelanders. *Cancer epidemiology, biomarkers & prevention : a publication of the American Association for Cancer Research, cosponsored by the American Society of Preventive Oncology* 1997;6(11):863-73. [published Online First: 1997/11/21]
55. Wang Y-Y, Zhang W, Li H-L, et al. Population attributable risks of cigarette smoking for deaths of all causes, all cancers and other chronic diseases among adults aged 40-74 years in urban Shanghai, China. *Chinese Journal of Cancer Research* 2015;27(1):59.
56. Shankar A, Yuan J-M, Koh W-P, et al. Morbidity and mortality in relation to smoking among women and men of Chinese ethnicity: the Singapore Chinese Health Study. *European journal of cancer* 2008;44(1):100-09.
57. Nilsson S, Carstensen J, Pershagen G. Mortality among male and female smokers in Sweden: a 33 year follow up. *Journal of epidemiology and community health* 2001;55(11):825-30.
58. Nordlund LA, Carstensen JM, Pershagen G. Are male and female smokers at equal risk of smoking-related cancer: evidence from a Swedish prospective study. *Scandinavian journal of public health* 1999;27(1):56-62.

For peer review only - http://bmjopen.bmj.com/site/about/guidelines.xhtml

59. Meyer J, Rohrmann S, Bopp M, et al. Impact of smoking and excess body weight on overall and site-specific cancer mortality risk. *Cancer Epidemiology and Prevention Biomarkers* 2015;24(10):1516-22.
60. Wen C-P, Tsai SP, Chen C-J, et al. The mortality risks of smokers in Taiwan: Part I: cause-specific mortality. *Preventive medicine* 2004;39(3):528-35.

For peer review only

For peer review only - http://bmjopen.bmj.com/site/about/guidelines.xhtml

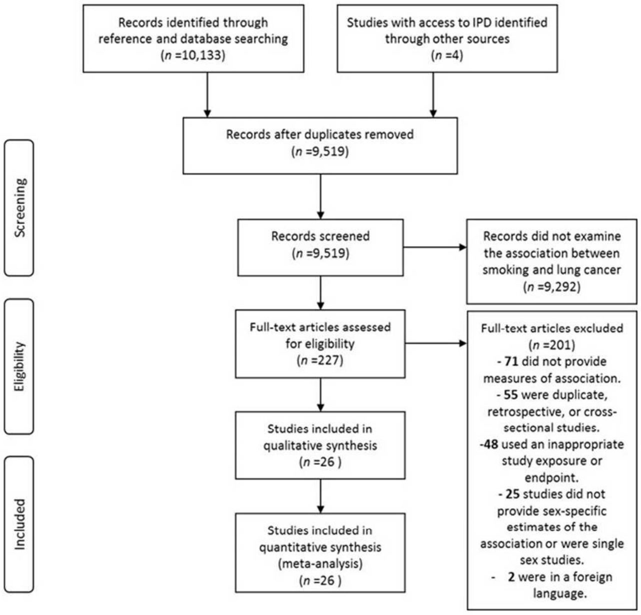

Figure 1: Flow diagram of study selection

90x90mm (300 x 300 DPI)

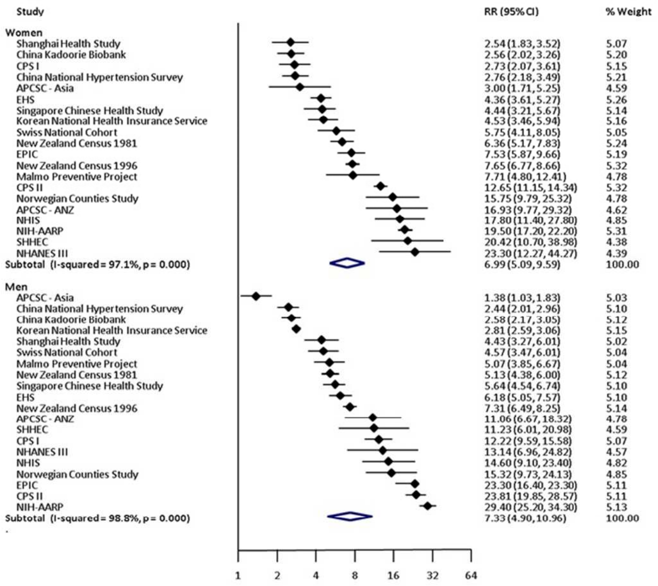

Figure 2: Multiple-adjusted relative risk for incident lung cancer in women and men, comparing current smokers to non-smokers

90x90mm (300 x 300 DPI)

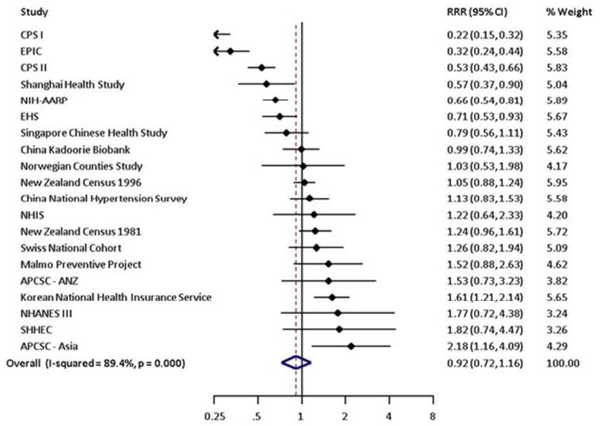

Figure 3: Multiple-adjusted women-to-men ratio of relative risks for incident lung cancer, comparing current smokers to non-smokers

90x90mm (300 x 300 DPI)

Supplementary Material

Smoking as a risk factor for lung cancer in women and men: a systematic review and meta-analysis

eAppendix 1: MOOSE checklist

eAppendix 2: Details of search strategy

eAppendix3: Quality assessment checklist

eTable 1: Quality assessment of the included studies

eFigure 1: Age-adjusted relative risk for incident lung cancer in women and men, comparing current smokers to non-smokers

eFigure 2: Age-adjusted women-to-men ratio of relative risks for incident lung cancer, comparing current smokers to non-smokers

eFigure 3: Contour funnel plots with pseudo 95% confidence limits for the ratio of relative risks associated with current and former smoking

eFigure 4: Age-adjusted relative risk for incident lung cancer in women and men, comparing former smokers to never smokers

eFigure 5: Age-adjusted women-to-men ratio of relative risks for incident lung cancer, comparing former smokers to never smokers

eFigure 6: Multiple-adjusted relative risk for incident lung cancer in women and men, comparing former smokers to never smokers

eFigure 7: Multiple-adjusted women-to-men ratio of relative risks for incident lung cancer, comparing former smokers to never smokers

For peer review only

For peer review only - http://bmjopen.bmj.com/site/about/guidelines.xhtml

eAppendix 1: MOOSE checklist

Item No	Recommendation	Reported on Page No
Reporting of background should include		
1	Problem definition	4
2	Hypothesis statement	4
3	Description of study outcome(s)	4
4	Type of exposure or intervention used	4
5	Type of study designs used	4
6	Study population	4
Reporting of search strategy should include		
7	Qualifications of searchers (eg, librarians and investigators)	6
8	Search strategy, including time period included in the synthesis and key words	6
9	Effort to include all available studies, including contact with authors	6
10	Databases and registries searched	6
11	Search software used, name and version, including special features used (eg, explosion)	6
12	Use of hand searching (eg, reference lists of obtained articles)	6
13	List of citations located and those excluded, including justification	13
14	Method of addressing articles published in languages other than English	6
15	Method of handling abstracts and unpublished studies	6
16	Description of any contact with authors	6
Reporting of methods should include		
17	Description of relevance or appropriateness of studies assembled for assessing the hypothesis to be tested	6&7
18	Rationale for the selection and coding of data (eg, sound clinical principles or convenience)	6&7
19	Documentation of how data were classified and coded (eg, multiple raters, blinding and interrater reliability)	6&7

For peer review only

For peer review only - http://bmjopen.bmj.com/site/about/guidelines.xhtml

20	Assessment of confounding (eg, comparability of cases and controls in studies where appropriate)	6&7
21	Assessment of study quality, including blinding of quality assessors, stratification or regression on possible predictors of study results	6
22	Assessment of heterogeneity	7
23	Description of statistical methods (eg, complete description of fixed or random effects models, justification of whether the chosen models account for predictors of study results, dose-response models, or cumulative meta-analysis) in sufficient detail to be replicated	7
24	Provision of appropriate tables and graphics	13-18
Reporting of results should include		
25	Graphic summarizing individual study estimates and overall estimate	14&15
26	Table giving descriptive information for each study included	12
27	Results of sensitivity testing (eg, subgroup analysis)	18
28	Indication of statistical uncertainty of findings	8&9

For peer review only

For peer review only - http://bmjopen.bmj.com/site/about/guidelines.xhtml

Item No	Recommendation	Reported on Page No
Reporting of discussion should include		
29	Quantitative assessment of bias (eg, publication bias)	eTable7
30	Justification for exclusion (eg, exclusion of non-English language citations)	6
31	Assessment of quality of included studies	eTable1
Reporting of conclusions should include		
32	Consideration of alternative explanations for observed results	10
33	Generalization of the conclusions (ie, appropriate for the data presented and within the domain of the literature review)	10
34	Guidelines for future research	11
35	Disclosure of funding source	3

From: Stroup DF, Berlin JA, Morton SC, et al, for the Meta-analysis Of Observational Studies in Epidemiology (MOOSE) Group. Meta-analysis of Observational Studies in Epidemiology. A Proposal for Reporting. *JAMA*. 2000;283(15):2008-2012. doi: 10.1001/jama.283.15.2008.

Transcribed from the original paper within the NEUROSURGERY® Editorial Office, Atlanta, GA, United Sates. August 2012.

For peer review only

eAppendix 1: MOOSE checklist

Item No	Recommendation	Reported on Page No
Reporting of background should include		
1	Problem definition	4
2	Hypothesis statement	4
3	Description of study outcome(s)	4
4	Type of exposure or intervention used	4
5	Type of study designs used	4
6	Study population	4
Reporting of search strategy should include		
7	Qualifications of searchers (eg, librarians and investigators)	6
8	Search strategy, including time period included in the synthesis and key words	6
9	Effort to include all available studies, including contact with authors	6
10	Databases and registries searched	6
11	Search software used, name and version, including special features used (eg, explosion)	6
12	Use of hand searching (eg, reference lists of obtained articles)	6
13	List of citations located and those excluded, including justification	13
14	Method of addressing articles published in languages other than English	6
15	Method of handling abstracts and unpublished studies	6
16	Description of any contact with authors	6
Reporting of methods should include		
17	Description of relevance or appropriateness of studies assembled for assessing the hypothesis to be tested	6&7
18	Rationale for the selection and coding of data (eg, sound clinical principles or convenience)	6&7
19	Documentation of how data were classified and coded (eg, multiple raters, blinding and interrater reliability)	6&7
20	Assessment of confounding (eg, comparability of cases and controls in studies	6&7

For peer review only - http://bmjopen.bmj.com/site/about/guidelines.xhtml

	where appropriate)	
21	Assessment of study quality, including blinding of quality assessors, stratification or regression on possible predictors of study results	6
22	Assessment of heterogeneity	7
23	Description of statistical methods (eg, complete description of fixed or random effects models, justification of whether the chosen models account for predictors of study results, dose-response models, or cumulative meta-analysis) in sufficient detail to be replicated	7
24	Provision of appropriate tables and graphics	13-18
Reporting of results should include		
25	Graphic summarizing individual study estimates and overall estimate	14&15
26	Table giving descriptive information for each study included	12
27	Results of sensitivity testing (eg, subgroup analysis)	18
28	Indication of statistical uncertainty of findings	8&9

For peer review only

For peer review only - http://bmjopen.bmj.com/site/about/guidelines.xhtml

Item No	Recommendation	Reported on Page No
Reporting of discussion should include		
29	Quantitative assessment of bias (eg, publication bias)	eTable7
30	Justification for exclusion (eg, exclusion of non-English language citations)	6
31	Assessment of quality of included studies	eTable1
Reporting of conclusions should include		
32	Consideration of alternative explanations for observed results	10
33	Generalization of the conclusions (ie, appropriate for the data presented and within the domain of the literature review)	10
34	Guidelines for future research	11
35	Disclosure of funding source	3

From: Stroup DF, Berlin JA, Morton SC, et al, for the Meta-analysis Of Observational Studies in Epidemiology (MOOSE) Group. Meta-analysis of Observational Studies in Epidemiology. A Proposal for Reporting. *JAMA*. 2000;283(15):2008-2012. doi: 10.1001/jama.283.15.2008.

Transcribed from the original paper within the NEUROSURGERY® Editorial Office, Atlanta, GA, United Sates. August 2012.

For peer review only

For peer review only

For peer review only - http://bmjopen.bmj.com/site/about/guidelines.xhtml
